# Rethinking solvent regeneration pathways for maritime carbon capture

**DOI:** 10.1038/s41467-026-74909-w

**Published:** 2026-06-30

**Authors:** Zhenrong Shi, Xiang Cao, Yongxin Hu, Teng Zhou

**Affiliations:** 1 Sustainable Energy and Environment Thrust, The Hong Kong University of Science and Technology (Guangzhou), Nansha, Guangzhou, China; 2 Department of Chemical and Biological Engineering, The Hong Kong University of Science and Technology, Hong Kong SAR, China

**Keywords:** Carbon capture and storage, Chemical engineering

## Abstract

Maritime transport is difficult to decarbonize, and onboard carbon capture has emerged as a promising option. However, adapting land-based capture systems may not suit the space, energy, and weight constraints of ships. Here we show that shifting solvent regeneration from ship to shore can improve the practicality and cost-effectiveness of maritime carbon capture. We compare onboard and onshore regeneration across 16 shipping routes using four capture solvents under different onboard load limits. The results show that adopting practical load limits (~20%), rather than fixed capture rates, provides a more reasonable basis for system design. Solvents with low regeneration energy are favored for onboard regeneration, whereas solvents with high carbon dioxide absorption capacity are preferred for onshore regeneration. With carbon tax included, onshore regeneration is generally more cost-effective in 2030 and 2050, and an extended analysis of 357 routes indicates it becomes economically viable by 2040 and broadly applicable by 2050.

## Abstract (web summary)

This study shows that shifting solvent regeneration from ships to shore makes maritime carbon capture more cost-effective. High-absorption-capacity solvents and a reasonable additional onboard weight make this strategy broadly applicable.

## Introduction

The continuous emission of greenhouse gases (GHGs) from human activities dramatically increases the likelihood of severe and potentially irreversible damage to ecosystems. To effectively address this issue, it is essential to achieve substantial and sustained reductions in these emissions^1^. In 2018, the shipping industry contributed to 2.89% of the global anthropogenic carbon emissions^2^. As part of the broader effort to combat climate change, the maritime sector is obligated to take its fair share of responsibility. The International Maritime Organization (IMO) has set comprehensive plans and targets to achieve net-zero GHG emissions from international shipping by approximately 2050, aligning with the long-term temperature objectives outlined in the Paris Agreement^3^. Additionally, initiatives like the European Union’s 2023 “Fit for 55” package and the IMO’s upcoming GHG standards and pricing mechanisms (set for 2027) all reflect global regulatory momentum toward emission reduction^4–7^.

Green shipping using alternative fuels, such as liquefied natural gas (LNG) and green methanol, can help decarbonize the maritime industry^8–14^. However, compared to conventional fuels, their lower energy density and, in many cases, higher costs currently make them less competitive^5,15^. Conventional oil-based fossil fuels still power 98.8% of the active fleet and account for 78.9% of new ship orders^16^. Battery-electric ships provide an additional decarbonization pathway; however, techno-economic assessments show that they are economically attractive mainly on short-distance routes^17^, and their competitiveness declines substantially on long-haul voyages, even when optimal offshore charging stations are considered^18^.

Given the above limitations, the carbon capture and storage (CCS) technology emerges as a viable low-carbon option for the shipping industry. CCS involves capturing CO_2_ from emissions for reuse or permanent storage^19–21^. The 81st session of the IMO’s Marine Environment Protection Committee has committed to developing a regulatory framework for onboard carbon capture and storage (OCCS) systems^3^. For maritime applications, chemical absorption-based post-combustion capture is most promising^22^. Chemical absorption uses amine solvents to capture CO_2_ in an absorber, followed by solvent regeneration in a stripper. The separated CO_2_ is then compressed, cooled, and stored in liquid form in specially designed tanks onboard. This approach offers a flexible and viable solution for emission reduction across various ship types and routes, presenting an immediate and potentially more effective solution^23^.

Previous research has confirmed the technical viability of OCCS using chemical absorption, while also highlighting its substantial energy and cost challenges. For instance, monoethanolamine (MEA)-based systems have achieved 90% CO_2_ capture on cargo ships, with reported regeneration energies of 3.85 GJ tonne^−1^ CO_2_ and avoidance costs of approximately 189 United States dollars (US$) tonne^−1^ CO_2_, converted using 1 euro (€) = US$1.16^24^. Another system-level optimization study reduced capital expenditure by 19% and lowered the CO_2_ avoidance cost to ~US$274 tonne^−1^ CO_2_^25^. Beyond MEA, advanced solvents have also been investigated. Piperazine (PZ) enables 90% capture on a 1280 kW vessel at a cost of ~US$240 tonne^−1^ CO_2_^26^. Blended amines based on methyldiethanolamine (MDEA) and PZ can achieve ~3.3 GJ tonne^−1^ CO_2_ for the same capture rate (CR)^27^, while an optimized MDEA-PZ configuration could increase capture efficiency to 94.7%^28^. Similarly, tetraethylenepentamine-MEA blends increase CO_2_ loading by 7% and reduce desorption heat duty by 18.1% compared to MEA^29^. Collectively, these studies indicate that solvent selection and process design are critical factors in OCCS performance.

In parallel, many companies are actively promoting research and development in this area^30–42^. In January 2024, the EVER TOP container ship completed a trial voyage equipped with an OCCS system^43^, and Seabound demonstrated onboard mineralization with CRs of ~1 t CO_2_ per day on a 3237 twenty-foot equivalent unit (TEU) vessel^44^. These demonstrations provide important proof-of-concept.

Despite being promising, OCCS is still in its early stages^6,45^. A key limitation in many studies is the assumption that exhaust-gas waste heat can be made available for solvent regeneration^24–27,46,47^. In practice, unfortunately, most recovered exhaust heat on ocean-going vessels is already committed to mandatory onboard heating demands, such as fuel heating and hoteling-related heating, leaving only limited residual heat for OCCS use^48,49^. Therefore, additional fuel must be consumed to support solvent regeneration. For example, a recent study on a commercial tanker reported that the additional fuel required to supply OCCS utilities would increase the ship’s CO_2_ emissions by 43%^50^. Tavakoli et al. further showed that achieving 70–90% emission reductions can increase the total fuel consumption by 70–100% relative to a baseline vessel without carbon capture^21^. Capture efficiency under real operating conditions is another concern. For example, the EVER TOP demonstration captures approximately 44,000 tonnes of CO_2_ per year, corresponding to an estimated annual CR of only 27%^51^. This issue is exacerbated by the low CO_2_ concentration in ship exhaust (~4.8%)^26^, which lies at the lower end of post-combustion capture applications (3–20 vol%)^52^ and leads to a reduced thermodynamic driving force and higher specific energy consumption^53^. Finally, the added weight of captured CO_2_ and storage tanks diminishes the cargo-carrying capacity, which is mentioned by previous studies, but the actual operation and economic impact have never been quantitatively evaluated^21,24^.

To address these challenges and enhance the OCCS practicability, an onshore regeneration configuration is evaluated (Supplementary Fig. 1). Unlike the traditional onboard regeneration systems, which involve absorption, desorption, compression, and conditioning, this scheme requires only absorption onboard. The CO_2_-rich solvent is stored on the ship and transported to ports for regeneration and conditioning using much cheaper or cleaner energy. This approach reduces ship fuel consumption and simplifies the onboard carbon capture system, while also minimizing the need for CO_2_ storage tanks and consequently improving safety. Ultimately, this method streamlines operations and leverages port infrastructure for more sustainable maritime practices. Similar concepts have also been explored in industrial practice, such as Value Maritime’s Filtree system^54^.

While the onshore regeneration scheme presents potential advantages, it also raises concerns due to its anticipated higher solvent demand compared to traditional methods. This increased demand could lead to higher costs, added weight, and reduced cargo capacity. These challenges necessitate a thorough evaluation to assess the overall impact of the scheme. In other words, a comprehensive analysis of its economic and environmental viability is essential before it can be recommended for implementation.

**Fig. 1:**
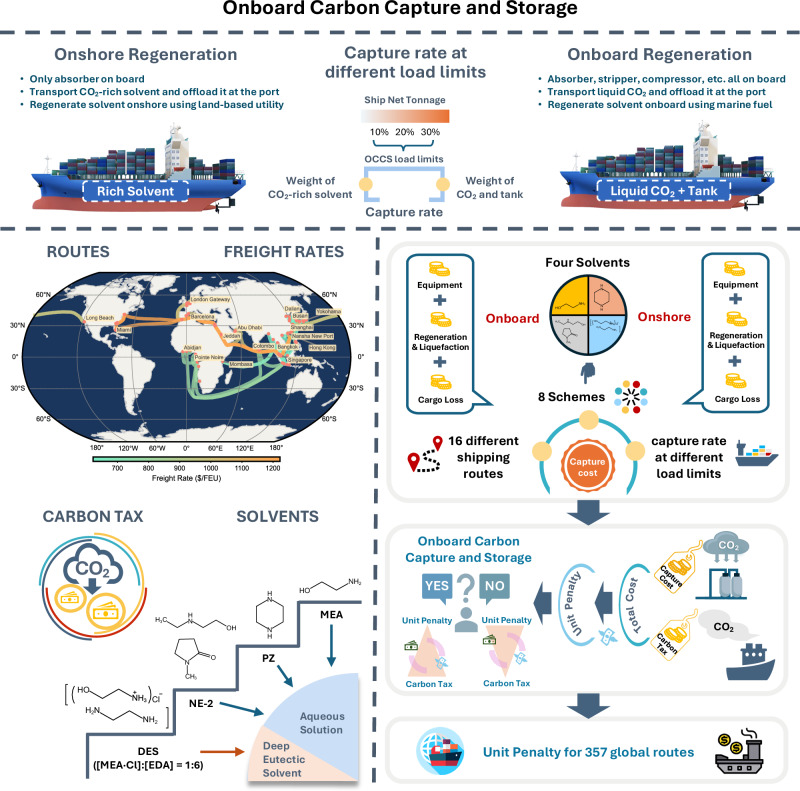
Research framework for evaluating onboard and onshore solvent regeneration in maritime carbon capture. The framework compares onshore regeneration, in which CO_2_-rich solvent is stored onboard and regenerated at port, with onboard regeneration, where CO_2_ absorption, regeneration, and conditioning occur on the vessel. The analysis considers 16 shipping routes, four solvents, different load limits, and future carbon taxes, yielding eight regeneration–solvent combinatorial schemes. These schemes are evaluated using capture cost, total cost with carbon tax and unit penalty, and the best-performing scheme is then extended to 357 global shipping routes. Some graphical elements and icons in this figure were adapted from resources designed by macrovector via Freepik.

In this study, we evaluate the technical feasibility and economic viability of onshore and onboard regeneration schemes for 16 selected Maersk container shipping routes (Table 1), each represented by the most frequently operating vessel class using conventional marine fuels^55^. Four different types of CO_2_ capture solvents are assessed. First, we analyze the overall load impact of the OCCS system to ensure its operational feasibility (to avoid exceeding the ship’s net tonnage). We then compare three key cost metrics—capture cost, total cost with carbon tax (TCT), and unit penalty—to provide a comprehensive assessment of both onshore and onboard regeneration schemes under different carbon tax scenarios. To improve robustness, we implement two methodological extensions that add policy and operational realism: an emissions trading system (ETS) equivalent, a route-specific carbon-pricing framework, and a flexible solvent offloading strategy. Finally, to evaluate the global applicability of our finding, we compute the unit penalty across an expanded dataset of 357 Maersk routes using the most effective OCCS technology identified from the study of the 16 selected routes (Fig. 1). This study advances OCCS techno-economic analysis by explicitly integrating cargo-capacity loss and carbon-pricing impacts across a global shipping network, moving beyond route-specific or case-by-case assessments.

**Table 1:** Route and Ship Specifications

Routes	Ship name	Container capacity (TEU)	$\eta \bullet {MCR}$ (kW)	Propulsion fuel consumption (tonne day^−1^)	${m}_{{{{\rm{CO}}}}_{2},{{\rm{prop}}}}$ (tonne day^−1^)
FEW 3	MAERSK ENSHI	13,676	58,344	224.0	694.5
FEW 6	COSCO KOREA	8501	56,287	216.1	670.0
TP17 Eastbound	NORTHERN JAGUAR	8814	48,535	186.4	577.8
AE11 Westbound	MSC FEBE	24,034	64,235	246.7	764.6
AE7 Eastbound	MAERSK CINCINNATI	15,516	46,716	179.4	556.1
AE7 Westbound	MAERSK HAMBURG	15,282	46,716	179.4	556.1
TP17 Westbound	MAERSK TAIKUNG	8112	58,344	224.0	694.5
TP2 Eastbound	MSC PALOMA	14,000	61,404	235.8	731.0
TP2 Westbound	MSC PALOMA	14,000	61,404	235.8	731.0
IA7	MAERSK QINZHOU	2806	13,668	52.5	162.7
IA8	GSL VINIA	5782	48,535	186.4	577.8
ME Eastbound	XIN HUANG PU	4250	31,076	119.3	369.9
ME Westbound	XIN HUANG PU	4250	31,076	119.3	369.9
IA1	REN JIAN 26	4395	31,076	119.3	369.9
IA88	MAERSK NORESUND	2086	11,475	44.1	136.6
PH5	MAERSK NINGBO	2546	18,411	70.7	219.2

## Results

### Onboard carbon capture and storage load restrictions

**Fig. 2:**
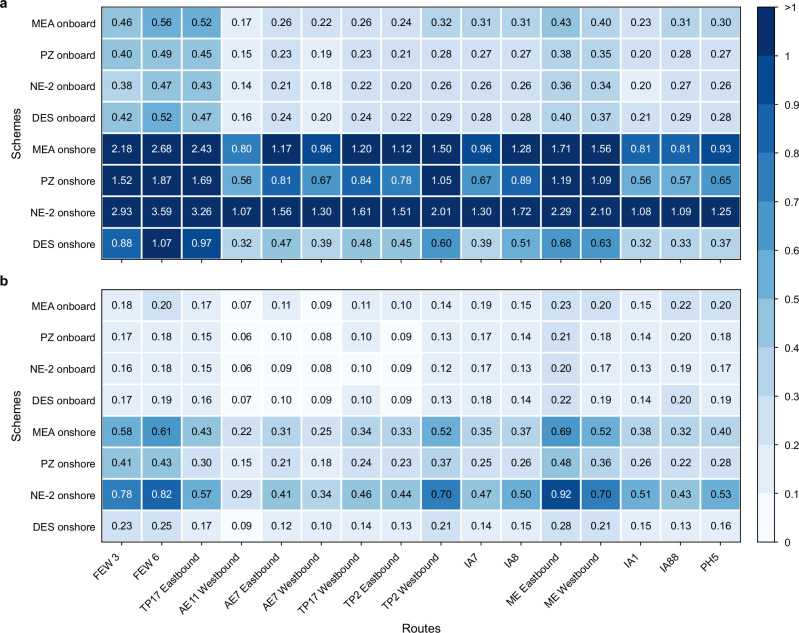
Ratio of onboard carbon capture and storage (OCCS) load to ship net tonnage at 90% capture rate. Heatmaps show these ratios for four solvents: monoethanolamine (MEA), piperazine (PZ), the water-lean solvent NE-2 and deep eutectic solvent (DES). Results are shown for onboard regeneration (top four rows) and onshore regeneration (bottom four rows) across 16 voyage routes. **a** Maximum ratio encountered in any voyage segment. **b** Average ratio across all segments of each voyage. Numeric labels give the exact ratio values. Values > 1 indicate that the OCCS load exceeds the vessel’s net tonnage, whereas values < 1 denote feasible installation.

Four solvents are considered: MEA, PZ, the water-lean solvent NE-2, and the deep eutectic solvent (DES). In total, eight carbon capture schemes—MEA onboard, PZ onboard, NE-2 onboard, DES onboard, MEA onshore, PZ onshore, NE-2 onshore, and DES onshore—are available for each route. Their feasibility is evaluated using the maximum OCCS-load-To-ship-net-tonnage ratio at a 90% CR, corresponding to the longest voyage segment. Theoretically, schemes with an OCCS-load-To-net-tonnage ratio below 1.0 are feasible. As shown in Fig. 2a, all the onboard regeneration schemes exhibit a ratio below 0.56, indicating that the load of liquefied CO_2_ and storage tanks has a relatively small impact on cargo capacity. This may explain why current OCCS demonstration projects prefer onboard regeneration.

Onshore regeneration schemes show significantly higher load ratios, with some exceeding 1.0, making them operationally impractical. A primary reason is that onshore regeneration requires the CO_2_-rich solvent to remain onboard before landing, which leads to a substantially higher load. For instance, in the case of 30 wt% MEA, capturing 1 tonne of CO_2_ in the onshore regeneration scheme necessitates storing 10.3 tonnes of rich solvent (Table 2), whereas onboard regeneration requires only storing 1 tonne of liquefied CO_2_. Another important factor is the maximum transportation time (Table 3). For example, the MEA onshore scheme for Route FEW 6 has a high ratio of 2.68, primarily due to its long maximum transportation period (24 days), which requires carrying an overweight solvent for this voyage segment. Therefore, it is more appropriate to apply a fixed load limit across all voyage segments $\left({\omega }_{{{\rm{limit}}}}\right)$.

**Table 2:** Solvent Information

Solvent type	Solvent abbreviation	Molecular weight	Rich-solvent weight ratio (t rich solvent t^−1^ CO_2_)	Regeneration heat (GJ t^−1^ CO_2_)
Amine solvent	MEA^68^	61.1	10.3	4.38
Amine solvent	PZ^68^	86.1	7.2	3.12
Water-lean solvent	NE-2^63^	106.4	13.8	2.54
Deep eutectic solvent	DES^64^	65.5	4.11	3.57

**Table 3:** Transportation time for various shipping routes

Routes	Total transportation days	Maximum transportation days ${t}_{{{\rm{s}}},\max }$
FEW 3	88	22
FEW 6	55	24
TP17 Eastbound	44	25
AE11 Westbound	49	15
AE7 Eastbound	57	18
AE7 Westbound	47	15
TP17 Westbound	50	16
TP2 Eastbound	22	15
TP2 Westbound	42	20
IA7	35	8
IA8	42	9
ME Eastbound	29	12
ME Westbound	22	11
IA1	42	6
IA88	35	5
PH5	33	6

Subsequently, the average OCCS-load-To-ship-net-tonnage ratio $\left({\omega }_{s}\right)$ at a 90% CR is shown in Fig. 2b. This route-averaged ratio varies substantially across routes, reflecting differences in solvent property and regeneration schemes, ship net tonnage, and inter-port transportation time. For example, the ME Eastbound route using NE-2 onshore scheme exhibits an average ratio as high as 0.92, largely attributable to a relatively small vessel (4250 TEU) and a long average transportation time (5.8 days), which leads to large rich-solvent accumulation on the ship. In contrast, routes such as AE11 westbound, operated by larger vessels (24,034 TEU) with comparable sailing durations (4.5 days), exhibit much lower load ratios. While this average ratio does not reflect any physical reality, as captured CO_2_ cannot be redistributed across voyage segments, it serves as a valuable indicator for selecting an appropriate load limit. Overall, most routes cluster at relatively low load limits, averaging around 0.25, which aligns with IMO reports indicating that ships typically utilize only about 70% of their capacity^56^. Based on these observations, setting an OCCS load limit of 20% is reasonable to maximize the CO_2_ CR without significantly reducing cargo capacity. For comprehensive analysis, we additionally evaluate scenarios with 10% and 30% load limits.

### Capture cost and capture rate for onboard and onshore schemes

The capture cost (CC) is calculated at 10%, 20%, and 30% load limits. The results indicate that the CR rises as the load limit increases. Moreover, the optimal solvent for each regeneration method becomes clear.

**Fig. 3:**
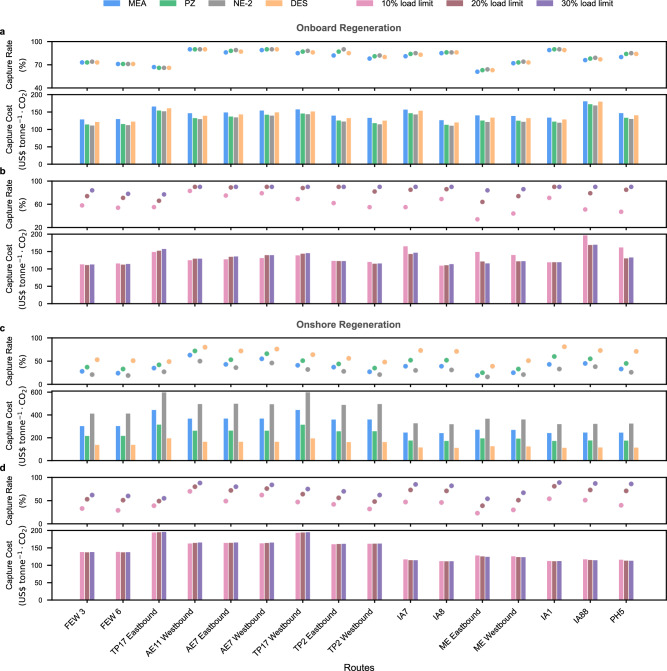
Capture cost and capture rate for different routes and regeneration schemes. Sixteen routes are shown on the x axis for all subfigures. Bars represent capture cost (US$ tonne^−1^ CO_2_); and circles indicate the corresponding capture rate. **a** Carbon capture rate and cost for onboard regeneration at a 20% load limit. Colors denote solvent: monoethanolamine (MEA, blue), piperazine (PZ, green), the water-lean solvent NE-2 (gray), and deep eutectic solvent (DES, orange). **b** Load limit sensitivity of onboard regeneration using NE-2. Bars and circles are rendered in three colors, representing a load limit of 10% (pink), 20% (red), and 30% (purple). **c** Carbon capture rate and cost for onshore regeneration at a 20% load limit. **d** Load limit sensitivity of onshore regeneration using DES.

Figure 3a shows the CC and rate for onboard regeneration schemes under a 20% CO_2_ storage OCCS load limit. The CCs range from US$100–185 tonne^−1^ CO_2_, with corresponding CRs of 60–90%. Among all the solvents evaluated, NE-2 demonstrates superior performance, achieving both the lowest CC and the highest CR across all routes. This advantage stems primarily from NE-2’s low regeneration heat requirement, which substantially reduces energy consumption during operation. As described in the Supplementary Method 13, the segment CR is fixed at 90% when the load limit $$({\omega }_{{{\rm{limit}}}})$$ is not reached but must be recalculated once the segment load exceeds $${\omega }_{{{\rm{limit}}}}$$. Under these conditions, the lower regeneration energy of NE-2 results in lower additional CO_2_ emissions $$({m}_{{{{\rm{CO}}}}_{2},{{\rm{add}}}})$$, thereby yielding a higher effective CR than the other solvents.

The results for the 10% and 30% CO_2_ storage load limits, presented in Supplementary Fig. 2a, b, show trends consistent with those observed for the 20% load limit. A detailed comparison of NE-2 performance is shown in Fig. 3b, demonstrating that higher load limits lead to increased CRs. In most cases, CCs also increase with the load limit, as the greater volume of CO_2_ captured requires larger onboard storage tanks and increases cargo loss, ultimately driving up expenses. However, in some cases, the CC at a 10% load limit exceeds that at 20%. This anomaly occurs in ships with capacities below 4300 TEU. For these relatively small ships operating at a low (10%) load limit, a large portion of the OCCS’s load capacity is occupied by CO_2_ storage tanks, thereby restricting the amount of CO_2_ that can be captured. As a result, the CC per tonne of CO_2_ becomes disproportionately high. In summary, NE-2 is identified as the optimal solvent, highlighting the advantages of low-regeneration-heat solvents (Table 2) in onboard regeneration systems.

For onshore regeneration schemes under a 20% rich-solvent storage load limit, CCs vary significantly depending on the solvent used (Fig. 3c). For example, using NE-2 can reach up to US$600 tonne^−1^ CO_2_ in some cases, while DES remains at around US$150 tonne^−1^ CO_2_. CRs range from 16% to 82%. CRs at the lower end of the distribution occur on routes characterized by small vessel sizes and long inter-port transportation times (e.g., the ME Eastbound route discussed in Fig. 2b), which also tend to exhibit elevated average OCCS load ratios, causing the solvent storage limit to be reached early in the voyage segment. Clearly, the solvent absorption performance follows the order: DES > PZ > MEA > NE-2. This trend is consistent across 10% and 30% rich-solvent storage load limits (Supplementary Fig. 2c, d). As shown in Fig. 2d (using DES as the solvent), the per-tonne CC is largely insensitive to the load limit, while the CR increases proportionally with increasing load. These results highlight the benefits of solvents with high absorption capacity (Table 2) in onshore regeneration systems, with DES demonstrating particularly strong performance.

**Fig. 4:**
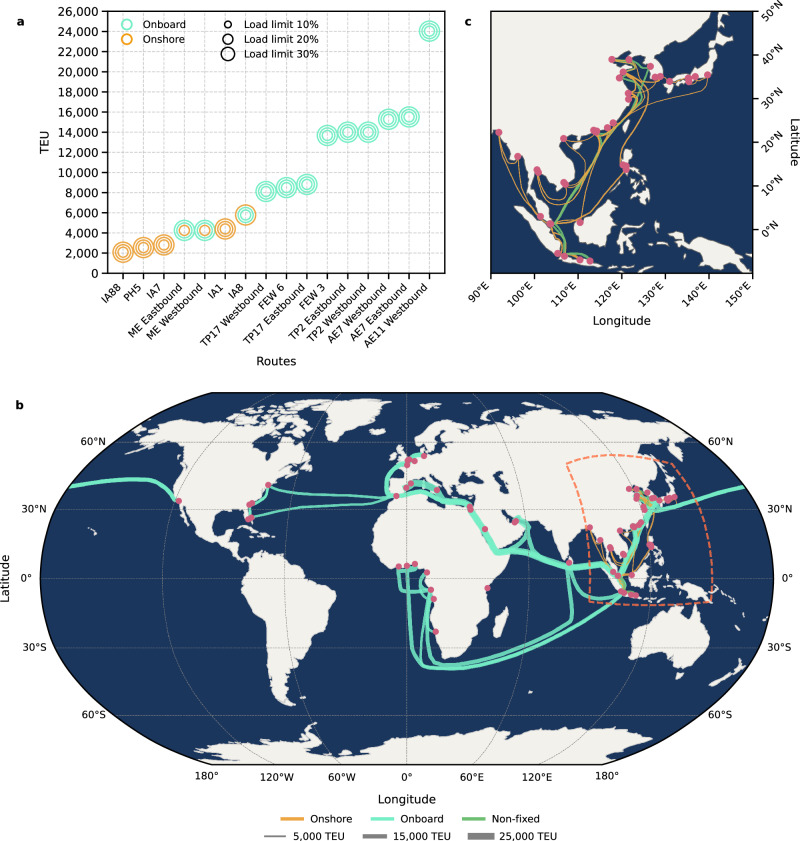
Regeneration scheme selections according to capture cost at 10%, 20%, and 30% load limits. **a** Route-level comparison of regeneration schemes with the lowest capture cost. Routes are ordered by ascending vessel capacity in twenty-foot equivalent unit (TEU). Marker color indicates the cost-optimal scheme (orange = onshore; mint = onboard), while marker size denotes the applied load limit. **b** Global Mollweide projection of the 16 shipping routes. Line color indicates the capture-cost-optimal regeneration scheme (orange = onshore; mint = onboard; muted leaf green = non-fixed) and line thickness (thin, medium, thick) denotes vessel capacity (5000, 15,000, 25,000 TEU, respectively). Pink circles mark intermediate ports of call. **c** Regional inset of the dotted area in subfigure (**b**), only highlighting routes where onshore regeneration is optimal.

Accordingly, NE-2 and DES, representing the optimal choices for onboard and onshore regeneration schemes, respectively, are compared across all 16 routes. Routes are sorted by ship TEU capacity to highlight size-dependent trends. As shown in Fig. 4, onboard regeneration proves more economical for larger vessels (TEU > 6000), whereas onshore regeneration is favored for smaller vessels (TEU < 6000). For a limited number of routes (three in this dataset), the preferred scheme switches due to marginal differences in CC between the two configurations.

Here, it is particularly noteworthy that CO_2_ emissions and CRs vary substantially between onboard and onshore regeneration schemes. Therefore, relying solely on per-tonne CCs provides an incomplete basis for equitable decision-making.

### Total cost with carbon tax and unit penalty

To fully assess the carbon reduction potential of the proposed OCCS schemes, it is essential to account for the CR, particularly under a carbon tax regime in the shipping industry. Calculating the TCT offers a comprehensive evaluation of each scheme’s economic viability. Furthermore, determining the carbon tax threshold that incentivizes OCCS adoption is crucial—a value that can be derived by converting total costs into a per-tonne CO_2_ unit penalty (UP).

**Fig. 5:**
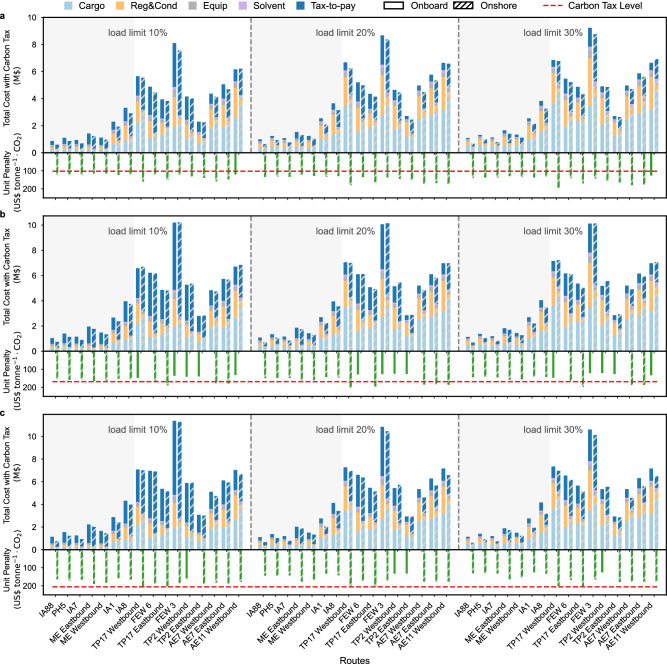
Total cost and unit penalty across 16 routes under future carbon‑tax scenarios. Subfigures **a**–**c** present results for the policy years 2030, 2040, and 2050, respectively. Within every subfigure, the upper chart shows the total cost with carbon tax (TCT) at three load limit constraints—10%, 20%, and 30%. Stacked bars partition the TCT into cargo loss cost (sky blue), regeneration plus conditioning cost (orange), fixed equipment cost (gray), solvent cost (purple), and carbon tax expenditure (navy blue). Solid bars correspond to the onboard regeneration scheme using the water-lean solvent NE-2, whereas diagonally hatched bars represent the onshore regeneration scheme using deep eutectic solvent (DES). Routes are ordered by vessel size; those shaded in light gray are ships with capacities below 6000 twenty-foot equivalent unit (TEU). The lower chart in each subfigure compares the prevailing carbon tax value (red dashed line) with the unit penalty imposed by the more economical regeneration scheme.

TCT of OCCS schemes is influenced by multiple factors, such as CC, CR, carbon tax, and emission factors. Figure 5 shows that for routes involving small ships, the onshore regeneration scheme with DES solvent consistently outperforms the onboard scheme using NE-2. For routes with large vessels, the onshore scheme remains the preferred choice at the 2030 carbon tax level of US$102.2, with the AE11 Westbound route as the only exception (Fig. 5a). This preference stems from the onshore scheme’s lower total OCCS cost $$({C}_{{{\rm{OCCS}}}})$$ in this context. Although the onboard scheme has a lower unit CC (Fig. 4), its higher CR and additional CO_2_ emissions $$({m}_{{{{\rm{CO}}}}_{2},{{\rm{add}}}})$$ drive up $${C}_{{{\rm{OCCS}}}}$$, making it comparatively more expensive. While the onboard scheme’s higher CR reduces carbon tax liabilities, this benefit is insufficient to offset its high $${C}_{{{\rm{OCCS}}}}$$ at the low 2030 carbon tax level. In fact, under the 2030 carbon tax, both schemes are less cost-effective than paying fines directly—a conclusion obtained from the UP analysis (lower chart of Fig. 5a).

At the projected 2040 carbon tax level of US$168.8, onboard regeneration becomes favorable in more scenarios (Fig. 5b). This transition results from the increased carbon tax, which reduces the TCT difference between the two approaches. Notably, OCCS emerges as a more cost-effective option than paying carbon taxes for many shipping routes at the 2040 tax level (lower chart of Fig. 5b).

By 2050, as the carbon tax rises further to US$205.9, onboard regeneration schemes might intuitively be expected to become more competitive compared to 2040; however, the results in Fig. 5c reveal the opposite trend. This outcome primarily stems from the decline in the onshore-regeneration emission factor (OEF) over time, which amplifies the advantage of onshore regeneration processes powered by renewable energy. From 2030 to 2040, the OEF is projected to decrease by roughly half (see Supplementary Table 30), yet it remains high enough to sustain the outcomes discussed earlier. By 2050, however, an additional order-of-magnitude reduction in OEF hedges the impact of rising carbon tax, especially at higher load limits with elevated CRs. This transition underscores the intrinsic economic and environmental advantages of onshore regeneration schemes by substantially reducing reliance on marine fuel for solvent regeneration and CO_2_ compression. Most notably, at the 2050 carbon tax level, OCCS achieves economic viability across all examined routes (lower chart of Fig. 5c), offering strong justification for its widespread adoption. Given the multidimensional nature of the cost evaluation, an online interactive visualization tool is provided to allow readers to quickly examine the CC and TCT results under specified parameters, such as sailing distance and transportation time.

To assess policy relevance under realistic regulatory conditions, we further evaluate the cost metrics under a route-specific ETS-equivalent carbon tax system. The resulting trends (Supplementary Fig. 3) closely mirror those obtained under the former uniform carbon-tax scenario, demonstrating the robustness of our conclusions across different carbon-pricing frameworks. Accordingly, the uniform carbon tax formulation is retained in the subsequent analysis for clarity.

### Flexible solvent offloading for the deep eutectic solvent-based onshore scheme

**Fig. 6:**
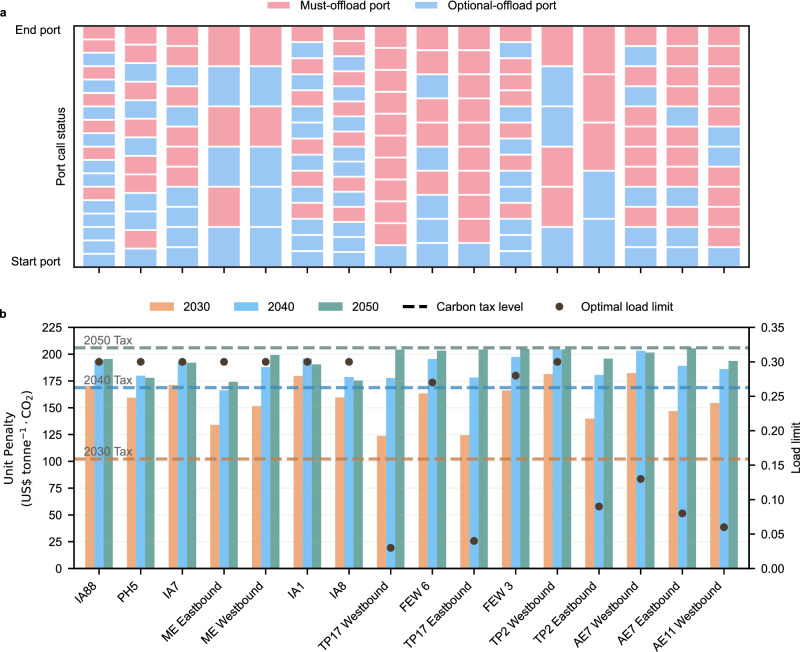
Flexible rich-solvent offloading results for the deep eutectic solvent-based onshore regeneration scheme. **a** Route-specific port offloading status under the flexible offloading strategy, where ports are classified as must-offload (red) or optional-offload (blue) based on minimization of the unit penalty. **b** Unit penalty under the flexible offloading strategy for different routes in 2030, 2040, and 2050. Bars indicate the unit penalty, dashed horizontal lines denote the carbon tax, and black dots represent the corresponding optimal load limit.

Based on the preceding analysis, the DES-based onshore regeneration scheme emerges as the most cost-effective option under most conditions. In practice, however, solvent offloading infrastructure may not be available at every port. To account for this constraint, we introduce another flexible offloading strategy, in which a subset of “must-offload” ports is identified for each route by minimizing its unit penalty (Fig. 6a and Supplementary Method 19). As shown, most routes can reduce offloading frequency substantially, whereas TP17 Westbound and Eastbound remain exceptions. These routes feature high freight rates and consequently need more frequent rich-solvent offloading to save cargo-loss cost.

As shown in Fig. 6b, OCCS remains uneconomical in 2030, consistent with the baseline results where rich solvent can be offloaded at every port. In 2040, only one route achieves a unit penalty below the corresponding carbon tax, in contrast to the multiple routes identified under the baseline scenario (Fig. 5b). This performance decline stems mainly from the increased onboard accumulation of CO_2_-rich solvent, which results in a higher cargo-loss penalty and reduces the effective CR. In 2050, all routes become economically viable, indicating that the DES-based onshore regeneration scheme remains promising even when offloading infrastructure is only available at a limited number of ports.

### Unit penalty across 357 routes using the deep eutectic solvent-based onshore scheme

To evaluate the global applicability of our findings, we compute the UP across 357 Maersk routes (Supplementary Data 3) using the DES onshore regeneration scheme with fixed load limits. Principal component analysis confirms that our initially studied 16 routes appropriately capture the characteristics of the full 357 routes (Supplementary Fig. 4). Due to data limitations, we apply the average freight rate from the 16 sample routes to estimate the cargo-loss costs for all routes.

**Fig. 7:**
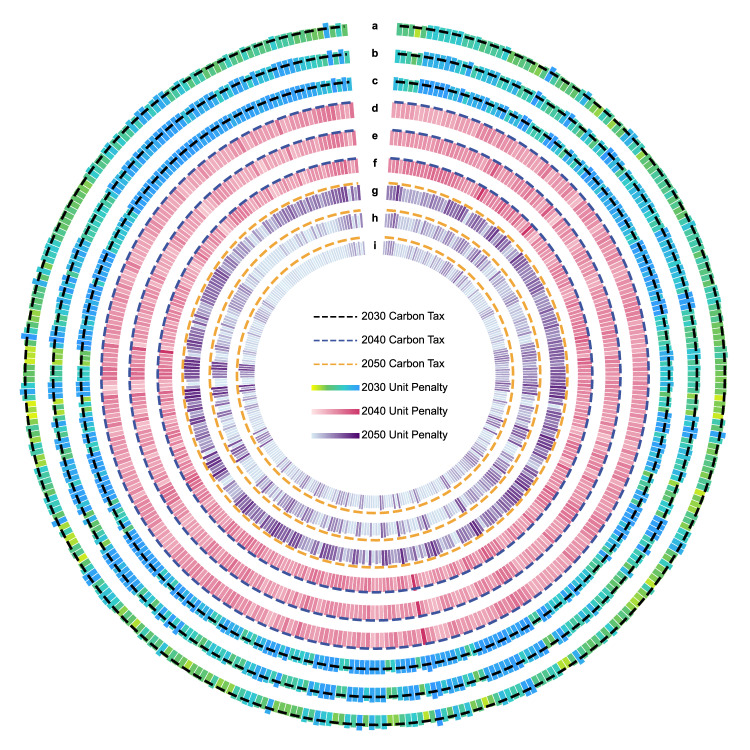
Economic viability of the deep eutectic solvent-based onshore regeneration scheme across 357 routes. Radial bars show the unit penalty (US$ tonne^−1^ CO_2_) for each of 357 routes, compared with projected carbon tax levels (dashed circles) for nine scenarios. Rings, from outer to inner, correspond to: **a** 2030 10%, **b** 2030 20%, **c** 2030 30%, **d** 2040 10%, **e** 2040 20%, **f** 2040 30%, **g** 2050 10%, **h** 2050 20%, **i** 2050 30% load limits. Bar height and color intensity jointly indicate the unit penalty magnitude; black, navy blue, and orange dashed circles mark the carbon taxes for 2030, 2040, and 2050, respectively. Bars extending below the tax circle indicate the economic advantage of the scheme in that scenario.

Figure 7 scales our analysis to 357 routes and replicates the trajectory observed in the initial 16 sample routes. In 2030, UP exceeds the carbon tax on every route, demonstrating that OCCS remains globally non-viable in the near term. In 2040, UP falls slightly below the carbon tax across all routes, indicating the potential for OCCS to offset carbon liabilities. By 2050, UP lies well below the tax in every case—especially at higher load limits—revealing a clear economic advantage.

These results highlight the growing competitiveness of OCCS with onshore regeneration as carbon pricing rises. While currently uneconomical, the onshore scheme is projected to become a key solution for decarbonizing international shipping by mid-century.

## Discussion

The central finding of this study is that the techno-economic viability of OCCS depends not only on solvent properties and capture performance, but also on where solvent regeneration is located within the broader ship–port system. Across the routes evaluated here, shifting regeneration location from ship to shore removes the most energy-intensive steps from the vessel, thereby reducing onboard fuel use, simplifying shipboard equipment, and alleviating the cost and safety constraints associated with onboard liquid-CO_2_ storage. The advantage of onshore regeneration is therefore not merely a change in process layout, but a system-level redistribution of energy demand and operational burden between ship and port. This perspective is important because much of the existing OCCS literature has focused on ship-level carbon capture performance, whereas route-scale deployment depends on the complex interactions between ship characteristics, voyage structure, carbon tax, and offloading logistics^57^.

A key implication of the present results is that practical OCCS design should be guided by feasible onboard load limits rather than capture-rate targets. Although high CRs are attractive from an emissions perspective, they can conflict with cargo-carrying capacity and shipping operations by increasing onboard storage requirements. Our results suggest that a load limit of around 20% of ship net tonnage provides a more realistic basis for evaluating different OCCS deployments across heterogeneous routes. This constraint also changes the decision-making for solvent selection. For onboard regeneration, NE-2 performs best because its low regeneration heat reduces the fuel and emissions penalty associated with shipboard desorption and conditioning. This preference is consistent with the general trend found in aqueous amine-based OCCS studies, where solvents with improved regeneration performance over the benchmark MEA are often favored^27,29^. By contrast, for onshore regeneration, DES performs best because its high CO_2_ absorption capacity reduces the mass of rich solvent that must be stored and transported before offloading. Solvent preference is therefore configuration-dependent, with onboard regeneration constrained mainly by energy demand and onshore regeneration by the storage burden of CO_2_-rich solvent.

The route-level analysis further shows that the relative attractiveness of OCCS configurations cannot be explained by any single route descriptor, but instead reflects the combined effects of ship size, inter-port sailing duration, fuel consumption, load limit, emission factor, and carbon tax. Accordingly, using a single cost metric, such as the cost per tonne of CO_2_ captured, is insufficient to represent OCCS performance across heterogeneous routes. OCCS should instead be assessed as a route-dependent operational strategy whose viability varies across vessel classes, port-call patterns, and emission regulations.

These findings are also instructive when compared with previous OCCS studies. Earlier research has primarily examined OCCS performance under overly idealized scenarios, in which the recovered heat from engine exhaust is assumed to be fully available for solvent regeneration^58^. In contrast, the present work emphasizes the importance of considering operational constraints and the additional fuel consumption associated with onboard regeneration. A narrow assessment boundary that excludes the ship’s existing energy utilization system may overestimate the competitiveness of onboard regeneration, whereas adopting a broader ship–port perspective reveals a greater potential for onshore regeneration, especially as shore-side energy becomes cleaner and the carbon tax continues to increase.

The robustness of the onshore regeneration configuration under both an ETS-equivalent carbon tax framework and a flexible solvent offloading strategy, together with the extended analysis of 357 Maersk routes, indicates that this conclusion is not limited to a few demonstration cases. From a practical deployment perspective, the onshore regeneration scheme should be better understood as a decarbonization strategy requiring ship–port coordination rather than as a purely onboard emissions control technology. In other words, its scalability depends on both advances in onboard CO_2_ capture and port-side regeneration and logistics infrastructure.

Several limitations of this study should be acknowledged. First, although absorber sizes are determined using standard industrial packed-column design methods, the capital costs for onboard OCCS equipment are not derived from detailed process simulations. This may lead to an under- or over-estimation of actual capital expenditures. Additionally, the economic evaluations fix fuel prices and freight rates and exclude investments in port-side supporting infrastructure, which may potentially change the evaluated scheme preferences. Uncertainties also persist regarding the production cost and long-term operational stability of novel solvents, particularly NE-2 and DES. Issues such as solvent corrosivity, degradation, and evaporative losses remain uncharacterized as well. Finally, projections of future carbon tax rates and emission factors introduce further uncertainty.

Future work should therefore combine detailed process simulation, life-cycle assessment, and port infrastructure planning to evaluate how and when OCCS can be integrated into broader maritime decarbonization pathways. Further extensions of the framework should consider alternative fuels, time–varying port availability for solvent offloading and regeneration, regional infrastructure development, and the evolving regulatory requirements to identify where onshore regeneration can provide the greatest practical advantage.

## Methods

The operational frameworks of onboard and onshore regeneration schemes reveal considerable differences in carbon emissions and costs. While a ship’s CO_2_ emissions are primarily determined by fuel consumption, the onboard regeneration scheme entails CO_2_ regeneration and conditioning processes that require additional fuel, leading to increased emissions. In contrast, the onshore regeneration scheme moves these processes to land-based facilities, thereby excluding the associated emissions from the ship’s direct profile. However, under carbon tax regimes, it is essential to include emissions from onshore regeneration facilities in cost analyses to ensure a comprehensive and equitable assessment of the economic and environmental trade-offs.

Additionally, the storage requirements for the two schemes differ significantly. For instance, capturing 1 tonne of CO_2_ onboard requires storage for an equivalent 1 tonne of liquefied CO_2_, whereas capturing the same amount of CO_2_ in an onshore regeneration scheme necessitates the storage of 10.3 tonnes of CO_2_-rich solvent when using 30 wt% MEA. Furthermore, the CO_2_ storage tanks used onboard are significantly heavier than the solvent storage systems employed in the onshore regeneration scheme. These disparities directly affect the achievable CRs under the same OCCS load limitation, further differentiating the CCs of the two schemes. In summary, all the factors mentioned above require thorough analysis to accurately assess the economic and operational viability of each OCCS scheme. Such evaluations are essential for guiding informed decision-making and advancing effective decarbonization strategies in the maritime sector under different carbon tax regimes.

### Onboard carbon capture and storage

#### Route information and emission calculation

We concentrate on 16 Maersk container shipping routes that pass through Nansha Port. For each route, we select the most prevalent type of ship operating on that route (refer to Table 1). The daily CO_2_ emissions from ship propulsion are estimated based on the specific route and ship specifications, as outlined in the Eq. (1).

1 $${m}_{{{{\rm{CO}}}}_{2},{{\rm{prop}}}}={MCR}\cdot \eta \cdot {SFOC}\cdot {EF}$$

where:

${m}_{{{{\rm{CO}}}}_{2},{{\rm{prop}}}}$ (tonne day^−1^) is the daily CO_2_ emission from the ship propulsion system.

${MCR}$ (kW) is the ship engine’s maximum continuous rating of each route.

η (%) is the engine load factor, assumed to be 85% of the full load capacity^24,59^.

${SFOC}$ (g kWh^−1^) is the specific fuel oil consumption, assumed 160 g kWh^−1^ for IFO 380 fuel^60^.

${EF}$ (tonne CO_2_ tonne^−1^ fuel) is the emission factor. For 1 tonne of fuel consumed, 3.1 tonnes of CO_2_ are produced^23^.

The implementation of OCCS can lead to additional CO_2_ emissions. The total daily emission (tonne day^−1^) for a ship is defined as Eq. (2):

2 $${m}_{{{{\rm{CO}}}}_{2}}={m}_{{{{\rm{CO}}}}_{2},{{\rm{prop}}}}+{m}_{{{{\rm{CO}}}}_{2},{{\rm{add}}}}$$

where ${m}_{{{{\rm{CO}}}}_{2},{{\rm{add}}}}$ (tonne day^−1^) represents the daily additional CO_2_ emissions generated from the implementation of the OCCS system. (i.e., ${m}_{{{{\rm{CO}}}}_{2},{{\rm{add}}}}=0$ for onshore regeneration). Detailed calculations are provided in Supplementary Methods 2 and 5.

A voyage typically comprises several segments, each with different sailing durations. The total CO_2_ emissions (in tonnes) for an entire voyage are calculated as Eq. (3).

3 $${M}_{{{{\rm{CO}}}}_{2}}={\sum}_{s}{t}_{s}\cdot {m}_{{{{\rm{CO}}}}_{2}}$$

where ${t}_{s}$ represents the transportation days for segments of the route and ${m}_{{{{\rm{CO}}}}_{2}}$ (Eq. (2)) is the daily CO_2_ emission of the ship. The Results are presented in Supplementary Data 1 and 2. It is clear that the emissions associated with onshore regeneration are independent of the durations of each segment. In contrast, the emissions for onboard regeneration are highly dependent on these conditions.

#### Solvent information

We evaluated four solvents with distinct physicochemical properties to span a broad and most relevant performance space for OCCS applications. Monoethanolamine (30 wt% MEA) is included due to its extensive industrial deployment^61^. Piperazine (PZ) is selected as a representative second-generation aqueous amine, offering faster kinetics and a lower reported regeneration heat compared to MEA^62^. Beyond conventional aqueous amines, advanced solvent classes are considered to reflect the specific constraints of OCCS. Water-lean solvents have attracted increasing interest due to their reduced regeneration duty arising from lower water content and higher cyclic CO_2_ capacity. In this context, the water-lean solvent NE-2, composed of 2-(ethylamino)ethanol (EMEA), 1-methyl-2-pyrrolidinone (NMP), and water, has demonstrated a regeneration heat as low as 2.2 GJ t^−1^ CO_2_ in pilot-scale studies, and is therefore included as a representative low-regeneration-energy solvent^63^. Notably, to keep consistency with ship-exhaust conditions, the regeneration heat is adjusted in this study, as detailed in Supplementary Method 4. Deep eutectic solvents (DES) are considered a distinct solvent class characterized by high mass absorption capacity, which can be potentially beneficial for our proposed onshore regeneration strategy involving long-time solvent storage prior to offloading. In this study, 60 wt% aqueous [MEA·Cl]:[EDA] DES (1:6) (denoted as DES) is selected as a representative system, exhibiting a reported CO_2_ mass loading of 32.15 wt%^64^.

Table 2 provides detailed information on each solvent with the calculations in Supplementary Method 4.

#### Ratio of onboard carbon capture and storage load to ship net tonnage

A typical carbon capture system achieves a CO_2_ recovery rate of 90%^65^. However, the implementation of OCCS technology increases a ship’s overall weight, and due to load capacity constraints, capturing all CO_2_ is impractical, resulting in a total CR lower than the theoretical maximum^66^. To assess the impact of the additional weight from OCCS systems on cargo capacity, it is assumed that the captured CO_2_ is unloaded at each port along the voyage. Given that a ship’s cargo capacity is limited by its net tonnage, the OCCS-load to ship-net-tonnage ratio for each voyage segment (*ω*_*s*_) is used to evaluate the feasibility of achieving the overall 90% CR.

For onboard regeneration, the primary concern is the weight of captured CO_2_ and associated high-pressure storage tanks (others are negligible). The OCCS load for onboard regeneration per segment (tonne) is defined as Eq. (4):

4 $${m}_{{{\rm{s}}},{{\rm{onboard}}}}={m}_{{{\rm{tank}}}}+{m}_{{{{\rm{CO}}}}_{2}}\cdot C{R}_{s}\cdot {t}_{s}$$

where ${t}_{s}$ represents the transportation days of segment *s*, $C{R}_{s}$ (%) is the segment CR and ${m}_{{{\rm{tank}}}}$ (tonne) is the weight of the storage tanks (Supplementary Data 1).

In contrast, for onshore regeneration, the weight of the CO_2_-rich solvent per segment (tonne) is primarily considered, with other weights neglected. This OCCS load is defined as Eq. (5):

5 $${m}_{{{\rm{s}}},{{\rm{onshore}}}}={m}_{{{{\rm{CO}}}}_{2}}\cdot C{R}_{s}\cdot \lambda \cdot {t}_{s}$$

where λ (*t* solvent t^−1^ CO_2_) is the rich-solvent weight ratio, as detailed in Supplementary Method 6 (see Table 2).

The OCCS-load-To-ship-net-tonnage ratio per segment ${\omega }_{s}$ is given by Eq. (6):

6 $${\omega }_{s}=\frac{{m}_{s}}{{net}\,{tonnage}}$$

Notably, ${t}_{s}$ varies significantly between different ports, as shown in Table 3, with some segments requiring considerably longer traveling time. Consequently, the segment with the maximum transportation days determines the maximum OCCS-load-To-ship-net-tonnage ratio and serves as a key parameter in assessing the economic feasibility of OCCS implementation.

The maximum OCCS-load-To-ship-net-tonnage ratio ${\omega }_{\max }$ is given by Eq. (7):

7 $${\omega }_{\max }=\frac{{m}_{{{\rm{s}}},\max }}{{net}\,{tonnage}}$$

where ${m}_{{{\rm{s}}},\max }$ (tonne) refers to the OCCS load of the segment with the maximum transportation days (Table 3) across the voyage. Detailed calculations of OCCS load and ${\omega }_{s}$ (at 90% CR) are provided in Supplementary Methods 7 and 8 and the results are in Supplementary Data 1 and 2.

#### Load limit and capture rate upper bound

To ensure that the additional load from OCCS remains manageable and does not interfere with the ship’s daily operations, a load limit, denoted as ${\omega }_{{{\rm{limit}}}}$, is established. Unlike ${\omega }_{s}$, which is based on a specific CR, ${\omega }_{{{\rm{limit}}}}$ is set arbitrarily and applied uniformly to each segment of the voyage.

When ${\omega }_{s}$ at a CR of 90% is smaller than ${\omega }_{{{\rm{limit}}}}$, CO_2_ is captured at 90% rate. However, when ${\omega }_{s}$ reaches the designated ${\omega }_{{{\rm{limit}}}}$, the OCCS system will halt, allowing excess emissions to be released directly into the atmosphere. Consequently, the CRs for that segment drop below 90% and must be recalculated (Supplementary Data 1 and 2). The overall capture rate (OCR) for the entire voyage is then given by Eq. (8):

8 $${OCR}=\frac{{\sum}_{s}{t}_{s}\cdot {m}_{{{{\rm{CO}}}}_{2}}\cdot {{CR}}_{s}}{{M}_{{{{\rm{CO}}}}_{2}}}$$

where ${{CR}}_{s}$ (%) represents the adjusted CR for segment *s*. The results of OCR are in Supplementary Data 1 and 2.

#### Solvent cost

Solvent cost is an important component of the economic assessment for OCCS. In this study, solvent cost is considered in two parts: (i) the initial solvent inventory required for system operation, and (ii) make-up solvent to account for degradation and loss during operation. All related assumptions, pricing data, and calculation procedures are provided in Supplementary Method 12.

#### Cost of cargo loss

The previously described OCCS load translates into a reduction in cargo capacity, which leads to economic losses determined by transport costs. To calculate the transportation cost per tonne of cargo, we use the freight rate per forty-foot equivalent unit (FEU) container and assume a total weight of 30.48 tonnes per FEU container^67^. Freight rates from the Shanghai Shipping Exchange are highly volatile, often influenced by global events such as wars or pandemics. To ensure more reliable results, freight rates impacted by the pandemic (2020–2022) are excluded, and the analysis is based on the average rates from the past decade (2014–2024). Detailed freight rate information is provided in Supplementary Table 20 in Supplementary Method 11, with an exchange rate of US$1 = 7.2 Chinese yuan.

#### Cost for onboard and onshore regeneration schemes

The total OCCS cost $${C}_{{{\rm{OCCS}}}}$$ (US$) for both onboard and onshore regeneration schemes include several key components: fixed equipment, regeneration, conditioning, solvent and cargo loss, as shown in Eq. (9).

9 $${C}_{{{\rm{OCCS}}}}={C}_{{{\rm{equip}}}}+{C}_{{{\rm{regeneration}}}}+{C}_{{{\rm{conditioning}}}}+{C}_{{{\rm{solvent}}}}{+C}_{{{\rm{cargo\; loss}}}}$$

Onboard regeneration relies on marine fuel, with costs calculated from fuel price and consumption. This study assumes the use of IFO 380 fuel at US$500 per tonne. The CO_2_ product concentration is assumed to be 99% by weight, with conditioning energy consumption modeled using Aspen Plus (Supplementary Method 3). Fixed equipment costs are estimated based on the designed absorber (Supplementary Method 9), while cargo loss costs (Supplementary Method 13) are determined according to the OCCS load, $${m}_{{{\rm{s}}},{{\rm{onboard}}}}$$.

Onshore regeneration is powered by low-pressure steam, while conditioning relies on plant electricity. The absorber cost is calculated based on the designed absorber and packing. Costs for other equipment are estimated using data from land-based carbon capture plants (Supplementary Method 10). Cargo loss costs are determined by ${m}_{{{\rm{s}}},{{\rm{onshore}}}}$, primarily weight of the CO_2_-rich solvent (Supplementary Method 14).

#### Carbon tax

Two different carbon taxes are used in this paper. First, a global carbon tax level is calculated using a weighted average based on each region’s share of global emissions (Supplementary Table 22) and their respective carbon tax rates (Supplementary Table 23). This approach captures regional disparities in emissions, yielding an estimate tailored to the global shipping industry. As a result, the projected carbon tax rates for 2030, 2040, and 2050 are US$102.2, US$168.8, and US$205.9, respectively. Second, to account for geographically varied carbon pricing regimes, including emissions trading systems (ETS), we introduce a route-specific ETS-equivalent carbon tax system. Specifically, each port is assigned a CO_2_ price taken from the IEA 2050-Net-Zero-Emission scenario according to its geographic location. Segment-level ETS-equivalent carbon taxes are then derived based on the carbon prices of the two connected ports, and these values are incorporated directly into the route-level total cost calculations for both onboard and onshore regeneration schemes. Meanwhile, a sailing-time-weighted average of all segment taxes is computed to obtain a single route-level ETS-equivalent carbon tax (Supplementary Table 25), which is used for comparison with a unit penalty to judge the economic viability of OCCS for each route. Detailed calculations are presented in Supplementary Method 15.

### Cost metrics

Three key cost metrics are calculated to comprehensively assess the potential of OCCS: CC (in US$ tonne^−1^ CO_2_), TCT (in US$), and Unit Penalty (UP, in US$ tonne^−1^ CO_2_). These metrics enable a comparison of different capture schemes and help identify the most suitable options under varying conditions.

#### Capture cost

The CC is calculated by dividing the total cost of the OCCS system by the mass of CO_2_ captured as shown in Eq. (10).

10 $${CC}=\frac{{C}_{{{\rm{OCCS}}}}}{{OCR}\cdot {M}_{{{{\rm{CO}}}}_{2}}}$$

The results are presented in Supplementary Data 6.

#### Total cost with carbon tax

Incorporating the CC, CR, and carbon tax, the total cost per voyage can be calculated for both onboard and onshore schemes.

The total cost for onboard regeneration scheme is defined as Eq. (11):

11 $${{TCT}}_{{{\rm{onboard}}}}={C}_{{{\rm{OCCS}}}}+\left(1-{OCR}\right)\cdot {M}_{{{{\rm{CO}}}}_{2}}\cdot {C}_{t}$$

where $${C}_{t}$$ (US$ tonne^−1^ CO_2_) is the carbon tax.

For onshore regeneration, emissions from the solvent regeneration and CO_2_ conditioning processes should be accounted for. Thus, the total cost for the onboard regeneration scheme is defined as Eq. (12):

12 $${{TCT}}_{{{\rm{onshore}}}}={C}_{{{\rm{OCCS}}}}+\left(1-{OCR}\right)\cdot {M}_{{{{\rm{CO}}}}_{2}}\cdot {C}_{t}+{OEF}\cdot {OCR}\cdot {M}_{{{{\rm{CO}}}}_{2}}\cdot {C}_{t}$$

where OEF (tonne CO_2_ emitted tonne^−1^ CO_2_ captured) represents the onshore regeneration scheme emission factor, generated as Eq. (13):

13 $${OEF}={RE}\times {SEF}+CE\times {EEF}$$

${RE}$ (GJ tonne^−1^ CO_2_) is the regeneration energy, ${SEF}$ (g CO_2_ kwh^−1^) is the emission factor of steam,${CE}$ (GJ tonne^−1^ CO_2_) is the conditioning energy, and ${EEF}$ (g CO_2_ kwh^−1^) is the emission factor of electricity. ${EEF}$ and ${SEF}$ are presented in Supplementary Tables 26 and 27, and the TCT results are provided in Supplementary Data 6 and 7.

#### Unit penalty

The unit penalty is calculated by dividing the total cost by the quantity of CO_2_ emitted. This metric allows for a direct comparison with the carbon tax, helping to determine whether implementing OCCS is more favorable than paying penalties for carbon emissions as Eq. (14):

14 $${UP}=\frac{{TCT}}{{M}_{{{{\rm{CO}}}}_{2}}}$$

The results are outlined in Supplementary Data 6 and 7.

## Supplementary information

Supplementary Information

Description of Additional Supplementary Files

Supplementary Data 1–8

Transparent Peer Review File

## Source data

Source data File

## Data Availability

Additional information supporting the findings of this study is available in Supplementary Information. Numerical data generated in this study and the source data underlying all main-text figures have been deposited in Zenodo under DOI: 10.5281/zenodo.20208567. An interactive visualization tool related to this study is publicly available at: https://occs-cost-explorer.onrender.com/. Source data are provided with this paper.
