# Supplementary material for: Rethinking solvent regeneration pathways for maritime carbon capture
Source: Nat Commun. 2026 Jun 30;17:8157. doi: 10.1038/s41467-026-74909-w (PMC13458838; doi:10.1038/s41467-026-74909-w)
Supplement: Supplementary file 1 [file 41467_2026_74909_MOESM1_ESM.pdf]

Supplementary Information

Rethinking Solvent Regeneration Pathways for Maritime Carbon Capture

Zhenrong Shi[1], Xiang Cao[1], Yongxin Hu[1], Teng Zhou[1,2,*]

[1] Sustainable Energy and Environment Thrust, The Hong Kong University of Science and Technology (Guangzhou), Nansha, Guangzhou 511400, China

[2] Department of Chemical and Biological Engineering, The Hong Kong University of Science and Technology, Hong Kong SAR, China

* Corresponding author: tengzhou@hkust-gz.edu.cn

Supplementary Items List

Supplementary Figures 3

Supplementary Figure 1. Conceptual diagram of onboard carbon capture and storage using onboard and onshore regeneration strategies. 3

Supplementary Figure 2. Capture cost and capture rate for different routes and regeneration schemes at 10% and 30% load limits. 4

Supplementary Figure 3. Total cost and unit penalty across 16 routes under route-specific emissions-trading-system-equivalent carbon tax scenarios. 5

Supplementary Figure 4. Principal-component projection of 357 container-shipping routes. 6

Supplementary Figure 5. Fitting curve for the coefficient of performance parameter as a function of temperature. 7

Supplementary Methods 8

Supplementary Methods 1. Routes and container vessels information 8

Supplementary Methods 2. Propulsion-related CO_2 emissions 11

Supplementary Methods 3. Energy requirement for CO_2 conditioning 13

Supplementary Methods 4. Regeneration heat estimation for different solvents 15

Supplementary Methods 5. Cost of regeneration and conditioning for different regeneration schemes 18

Supplementary Methods 6. Rich-solvent weight ratio 21

Supplementary Methods 7. Ratio of onboard carbon capture and storage load to ship net tonnage for the onboard regeneration scheme at 90% capture rate 22

Supplementary Methods 8. Ratio of onboard carbon capture and storage load to ship net tonnage for the onshore regeneration scheme at 90% capture rate 25

Supplementary Methods 9. Fixed equipment cost for the onboard regeneration scheme 26

Supplementary Methods 10. Fixed equipment cost for the onshore regeneration scheme 37

Supplementary Methods 11. Transportation cost 40

Supplementary Methods 12. Solvent cost 41

Supplementary Methods 13. Capture cost for the onboard regeneration scheme 44

Supplementary Methods 14. Capture cost for the onshore regeneration scheme 46

Supplementary Methods 15. 2030–2050 Carbon taxes and emission factors 48

Supplementary Methods 16. Total cost with carbon tax for onboard regeneration 52

Supplementary Methods 17. Total cost with carbon tax for onshore regeneration 53

Supplementary Methods 18. Unit penalty 54

Supplementary Methods 19. Flexible offloading strategy for onshore regeneration scheme 55

Supplementary Methods 20. Parameter specifications and nomenclature 58

Supplementary References 66

Supplementary Figures

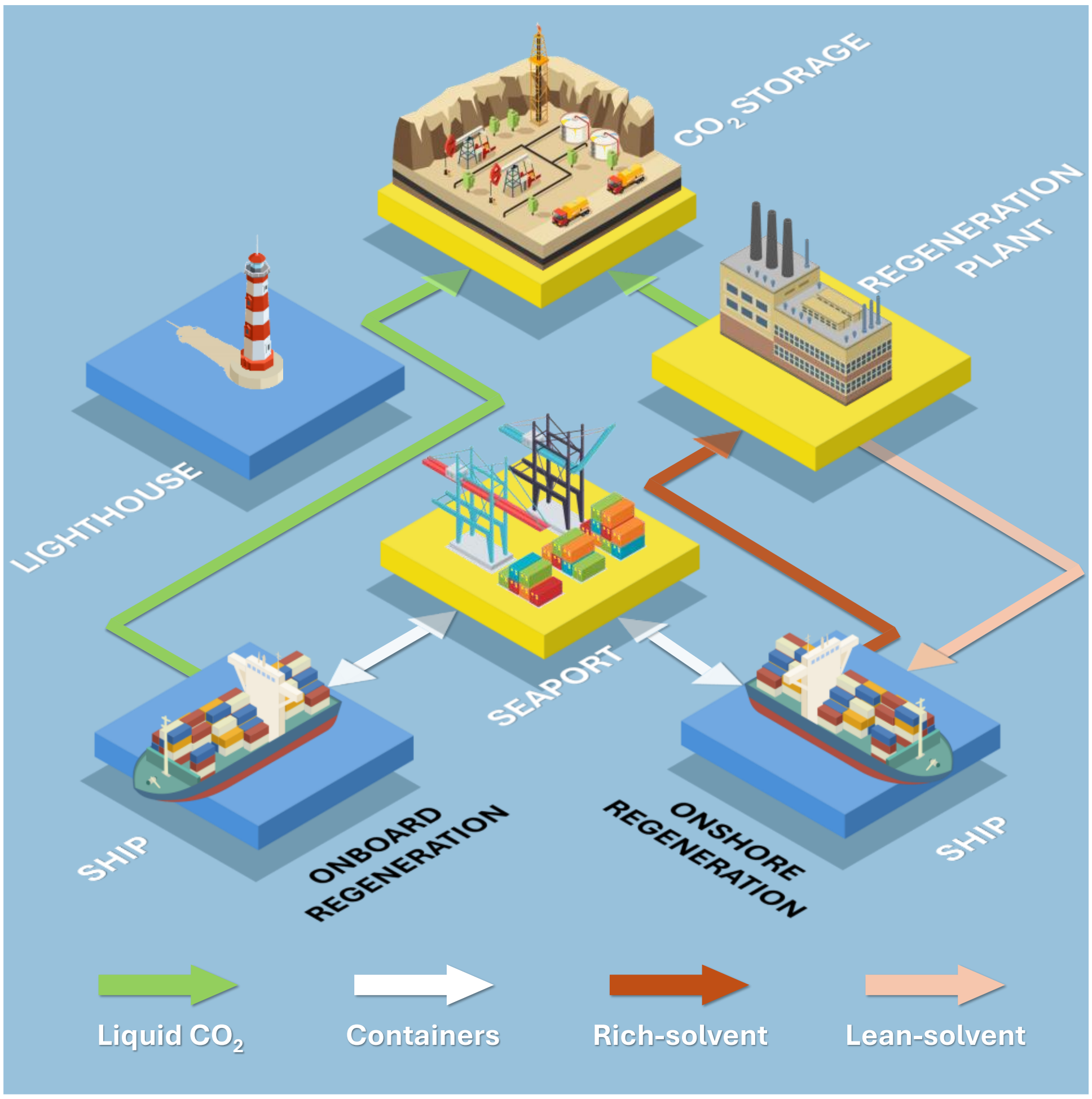

Supplementary Figure 1. Conceptual diagram of onboard carbon capture and storage using onboard and onshore regeneration strategies. This schematic compares two regeneration configurations for onboard carbon capture and storage (OCCS) systems in maritime transport. In the onboard regeneration strategy, captured CO_2 is regenerated, liquefied, and stored directly on the vessel before being transported to port for offloading and permanent storage. In the onshore regeneration strategy, carbon-rich solvents are transported from the ship to a port-based regeneration plant, where solvent regeneration and carbon dioxide conditioning are performed using land-based infrastructure. The regenerated lean solvent is subsequently returned to the vessel for reuse. The diagram highlights the material transportation pathways associated with the two strategies, including liquid CO_2 transport, solvent circulation, and container-based logistics between ships, ports, regeneration facilities, and CO_2 storage sites. Some graphical elements and icons in this figure were adapted from resources designed by macrovector via Freepik.

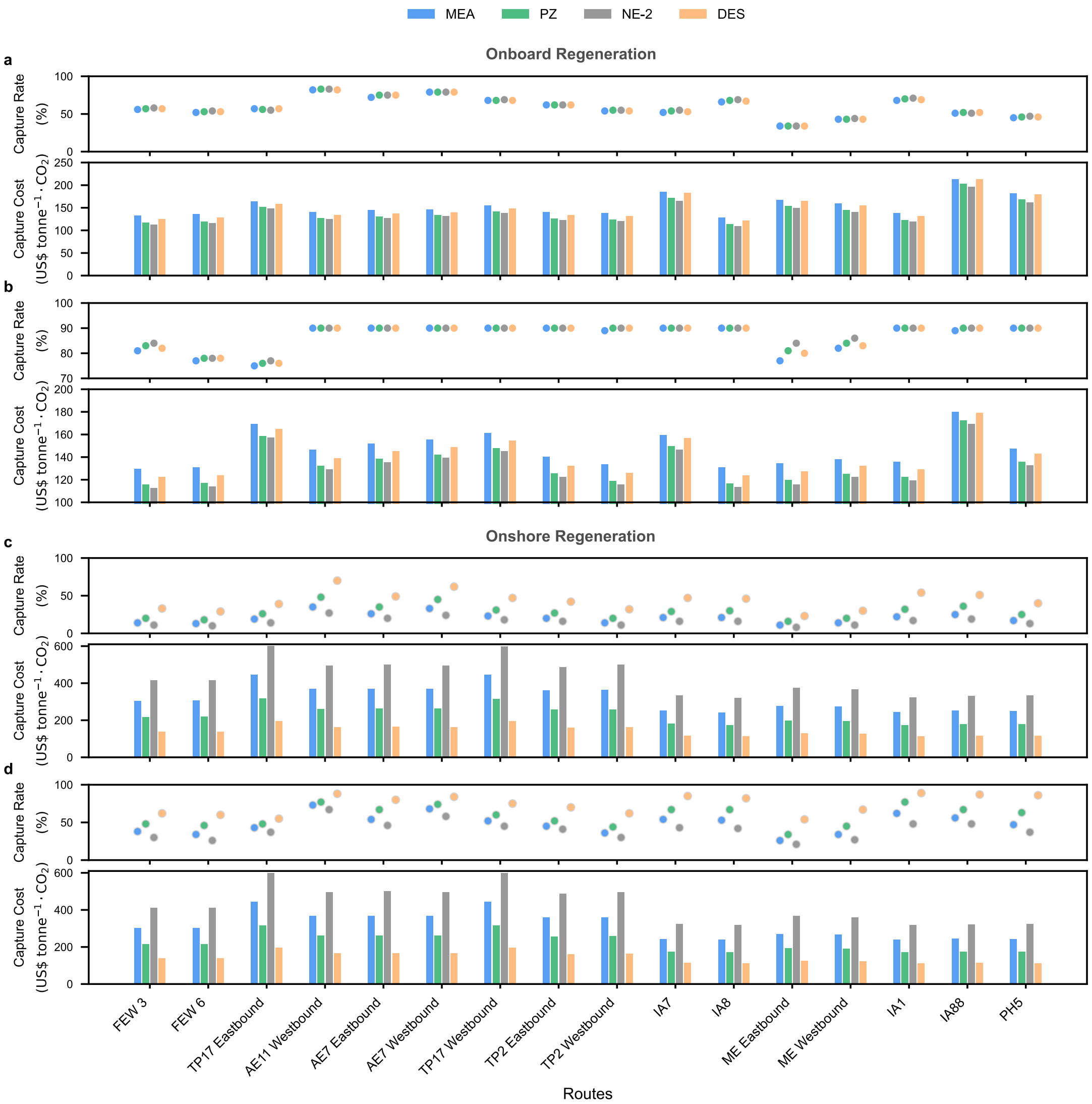

Supplementary Figure 2. Capture cost and capture rate for different routes and regeneration schemes at 10% and 30% load limits. Sixteen routes are shown on the *x*-axis for all subfigures. Bars represent capture cost (US\$ tonne$^{-1}\cdot CO_2$), and circles indicate the corresponding capture rate. Colors denote solvent: monoethanolamine (MEA, blue), piperazine (PZ, green), the water-lean solvent NE-2 (grey) and deep eutectic solvent (DES, orange). a, Carbon capture rate and cost for onboard regeneration at a 10% load limit. b, Capture rate and cost for onboard regeneration at a 30% load limit. c, Capture rate and cost for onshore regeneration at a 10% load limit. d, Capture rate and cost for onshore regeneration at a 30% load limit.

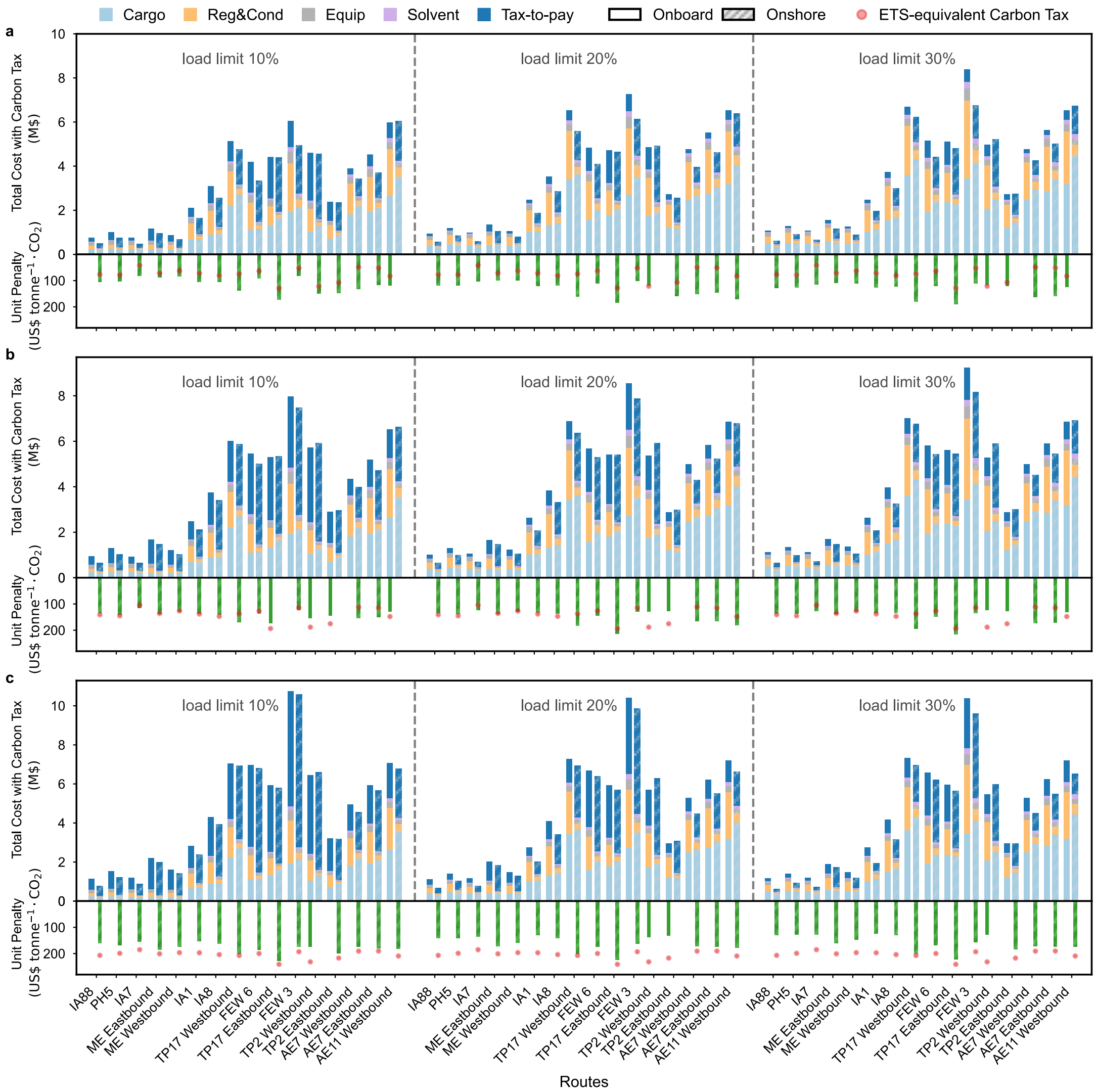

Supplementary Figure 3. Total cost and unit penalty across 16 routes under route-specific emissions-trading-system-equivalent carbon tax scenarios. Subfigures (a–c) present results for the policy years 2030, 2040 and 2050, respectively. Within every subfigure, the upper chart shows the total cost with carbon tax (TCT) at three load-limit constraints—10%, 20% and 30%. Stacked bars partition the TCT into cargo-loss cost (sky blue), regeneration plus conditioning cost (orange), fixed equipment cost (grey), solvent cost (purple) and carbon-tax expenditure (navy blue). Solid bars correspond to the onboard-regeneration scheme using the water-lean solvent NE-2, whereas diagonally hatched bars represent the onshore-regeneration scheme using deep eutectic solvent (DES). Routes are ordered by vessel size. The lower chart in each subfigure compares the emissions-trading-system-equivalent carbon-tax value (red dot) with the unit penalty imposed by the more economical regeneration scheme.

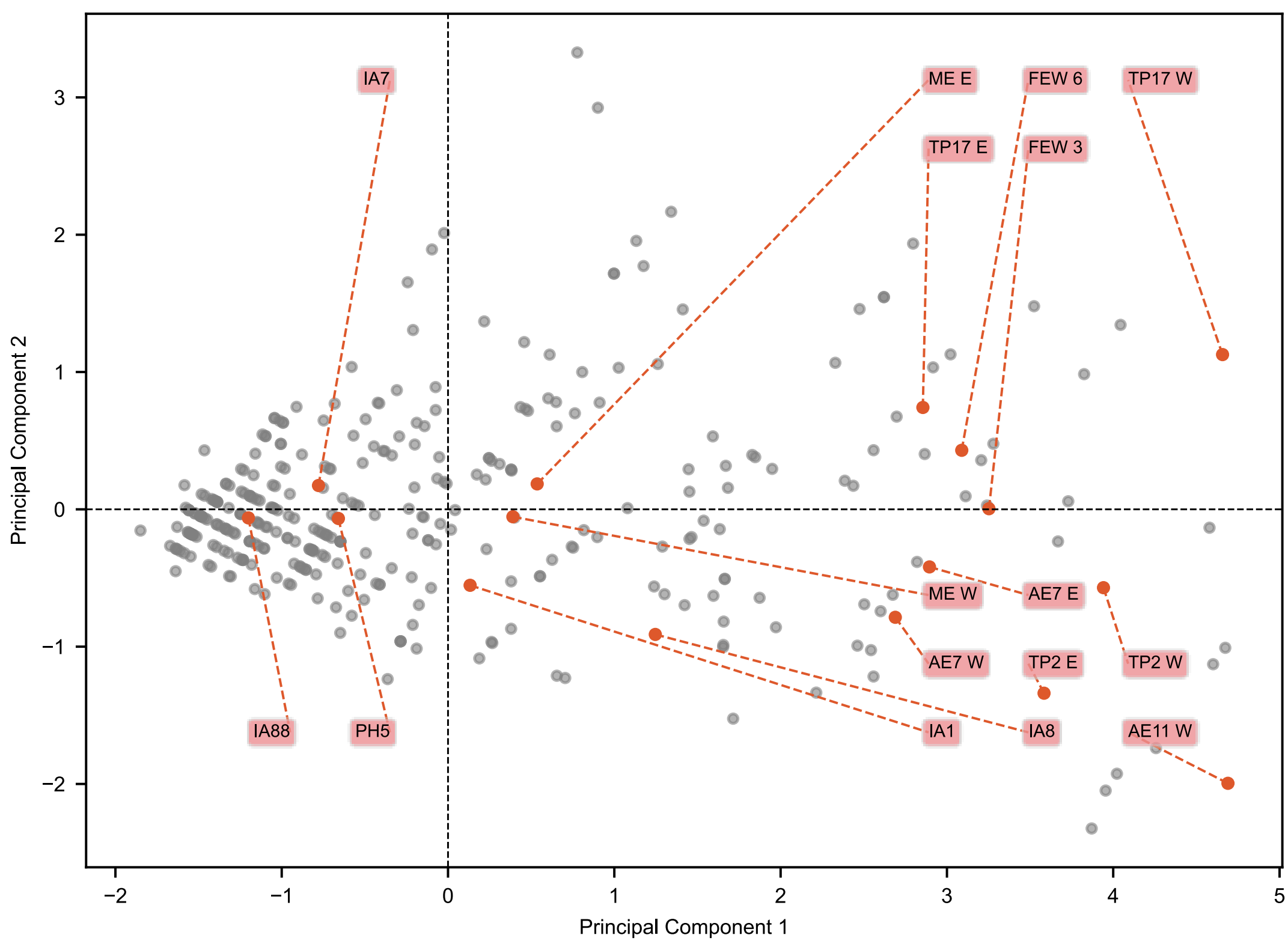

Supplementary Figure 4. Principal-component projection of 357 container-shipping routes. Grey points represent all routes, and the 16 routes selected for detailed onboard carbon capture and storage (OCCS) analysis are highlighted as red circles. Four operational attributes—maximum transportation time, total transportation time, main-engine power and vessel capacity—were z-score standardized and projected onto the first two principal components, explaining 95.66% of the total variance. The wide dispersion of the 16 routes across the biplot shows that they span the full operational envelope of the global fleet. The cluster results are provided in the Source Data file.

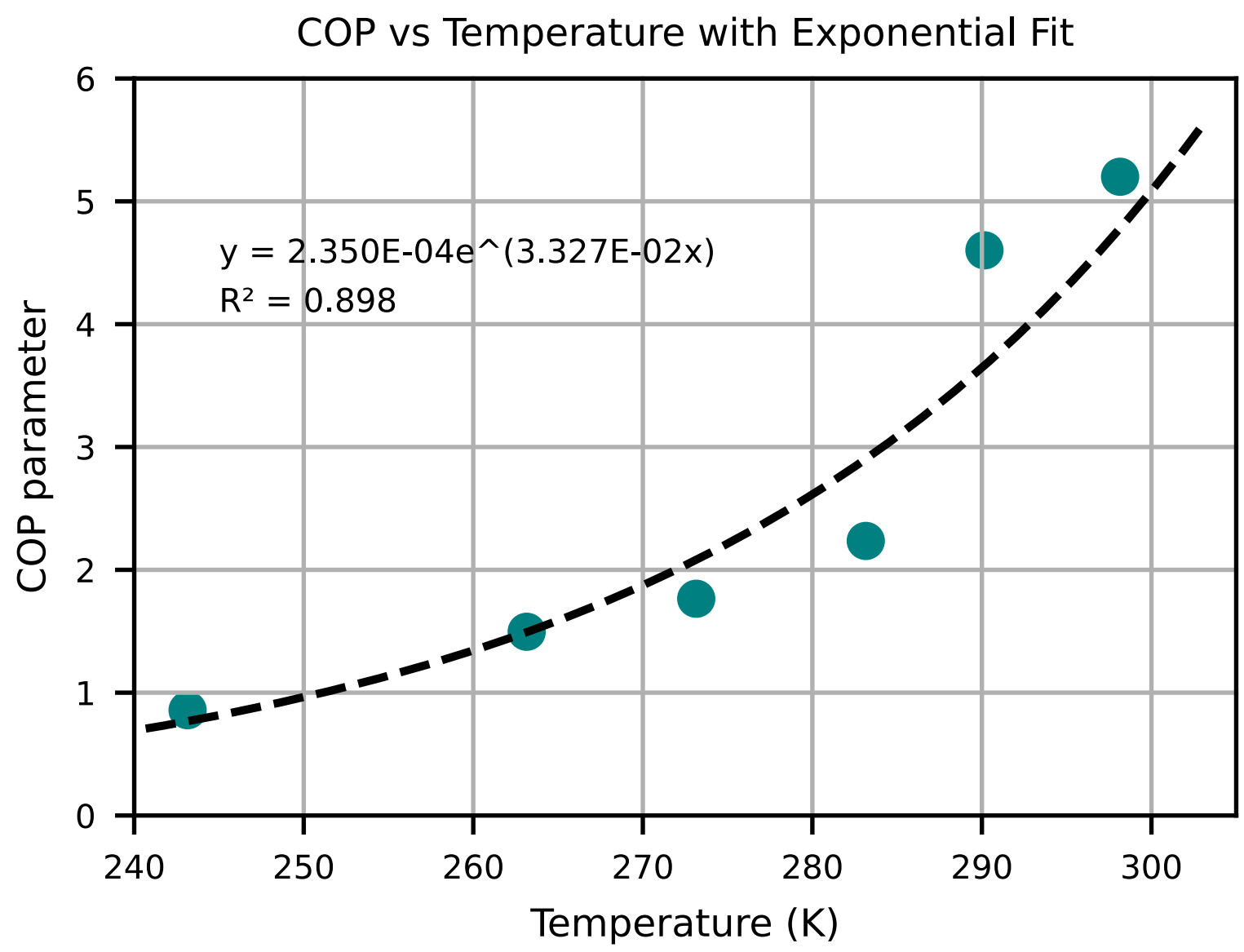

Supplementary Figure 5. Fitting curve for the coefficient of performance parameter as a function of temperature. Experimental coefficient of performance (COP) data at different temperatures are fitted with an exponential function. The fitted relationship is used to estimate the refrigeration electricity demand for onboard CO_2 conditioning.

Supplementary Methods

Supplementary Methods 1. Routes and container vessels information

Supplementary Tables 1-3 present the basic information of the 16 container shipping routes passing through the Nansha port. The data includes vessel registration details (vessel name, IMO number, classification society, and flag state), vessel dimensions (length, width, deadweight tonnage, net tonnage, and container capacity), as well as the propulsion engine model for each corresponding ship.

Supplementary Table 1. Registration details of container ships corresponding to the routes

Routes	Ship name	IMO number	Registered society	Flag	Distance (km)
FEW 3	MAERSK ENSHI[1]	9502946	DNV[2]	Panama (PA)	48949
FEW 6	COSCO KOREA[3]	9448750	DNV[2]	Hong Kong China (HK)	25861
TP17 Eastbound	NORTHERN JAGUAR[4]	9466972	DNV[2]	Portugal (PT)	24878
AE11 Westbound	MSC FEBE[5]	9839478	DNV[2]	Panama (PA)	20013
AE7 Eastbound	MAERSK CINCINNATI[6]	9936410	DNV[2]	Hong Kong China (HK)	26959
AE7 Westbound	MAERSK HAMBURG[7]	9784312	ABS[8]	Singapore (SG)	23017
TP17 Westbound	MAERSK TAIKUNG[9]	9334662	ABS[8]	Hong Kong China (HK)	28275
TP2 Eastbound	MSC PALOMA[10]	9441001	DNV[2]	Panama (PA)	13122
TP2 Westbound	MSC PALOMA[10]	9441001	DNV[2]	Panama (PA)	15184
IA7	MAERSK QINZHOU[11]	9858709	NKK[12]	Singapore (SG)	13054
IA8	GSL VINIA[13]	9280653	BV[14]	Liberia (LR)	15550
ME Eastbound	XIN HUANG PU[15]	9310044	CCS[16]	China (CN)	14135
ME Westbound	XIN HUANG PU[15]	9310044	CCS[16]	China (CN)	12860
IA1	REN JIAN 26[17]	9450947	CCS[16]	China (CN)	14459
IA88	MAERSK NORESUND[18]	9894686	NKK[12]	Panama (PA)	15459
PH5	MAERSK NINGBO[19]	9402639	ABS[8]	Hong Kong China (HK)	15050

Supplementary Table 2. Container ship dimensions for the routes investigated

Routes	Length (m)	Width (m)	Deadweight Tonnage (tonne)	Net Tonnage (tonne)	Container capacity (TEU[a])
FEW 3	366	48.27	147340	64587	13676
FEW 6	334	42.86	102710	55451	8501
TP17 Eastbound	333.59	43.28	108731	54884	8814
AE11 Westbound	399.9	61.54	254042	132544	24034
AE7 Eastbound	365.98	51.09	172053	79275	15516
AE7 Westbound	353.02	53.5	178257.3	79806	15282
TP17 Westbound	332	43.2	113478.4	85378	8112
TP2 Eastbound	365.49	51.28	190343	90033	14000
TP2 Westbound	365.49	51.28	190343	90033	14000
IA7	185.99	35.6	37500	12,452	2806
IA8	277.28	40	73260	37549	5782
ME Eastbound	251.4	32.2	52247	24,001	4250
ME Westbound	251.4	32.2	52247	24,001	4250
IA1	260.25	32.2	54370	25,378	4395
IA88	171.93	32.2	28,697	7,742	2086
PH5	208	29	34194	12996	2546

[a] TEU: Twenty-foot Equivalent Unit

Supplementary Table 3. Container ship engine model corresponding to the routes

Routes	Propulsion engines
FEW 3	Winterthur Gas & Diesel Ltd. 12RT-flex96C-B
FEW 6	MAN Energy Solutions, branch of MAN Energy Solutions SE, Germany 11K98ME-C
TP17 Eastbound	MAN Diesel & Turbo, Filial of MAN Diesel & Turbo SE, Tyskland 10K98ME-C
AE11 Westbound	MAN Diesel & Turbo, Filial of MAN Diesel & Turbo SE, Tyskland 11G95ME-C
AE7 Eastbound	MAN Diesel 8G95ME-C10.6-EGRTC
AE7 Westbound	HHI-MAN 8G95ME-C9.5
TP17 Westbound	DOOSAN ENGINE CO.LTD. 12RT-FLEX96C
TP2 Eastbound	12K98 MC-C
TP2 Westbound	12K98 MC-C
IA7	Mitsui E&S Machinery Co., Ltd. Tamano Machinery Factory 1DE: 2 SA 6 CY
IA8	(1) 2T – 10 cyl – 98.00 cm × 240.00 cm at 104 rpm
ME Eastbound	MAN B&W 8K90MC-C
ME Westbound	MAN B&W 8K90MC-C
IA1	MAN B&W 8K90MC-C
IA88	Mitsui E&S Machinery Co., Ltd. Tamano Machinery Factory 1DE: 2 SA 6 CY
PH5	CSSC-MES DIESEL CO., LTD. 6K80MCC

Supplementary Methods 2. Propulsion-related CO_2 emissions

The calculation of propulsion-related CO_2 emissions $m_{\mathrm{CO_2,prop}}$ begins with estimating the ship's fuel consumption. The daily fuel consumption for propulsion FC_{prop} is calculated as Supplementary Equation (1):

$$FC_{\mathrm{prop}} = \eta \cdot SFOC \cdot MCR \tag{1}$$

where:

η is the engine load factor, assumed to be 85% of full load capacity.[20,21]

$SFOC$ is the specific fuel oil consumption, assumed to be 160 $\mathrm{g \cdot kWh^{-1}}$ for IFO 380 fuel.[22,23]

MCR is the ship engine's maximum continuous rating of each route.

To convert fuel consumption to CO_2 emissions, the emission factor (EF) is applied. This factor assumes 3.1 tonnes of CO_2 are emitted per tonne of fuel consumed.[24] Thus, the propulsion CO_2 emissions per day are given by Supplementary Equation (2):

$$m_{\mathrm{CO_2,prop}} = FC_{\mathrm{prop}} \cdot EF \tag{2}$$

To determine the propulsion-related CO_2 emissions over a segment ($m_{\mathrm{CO_2,prop,s}}$), the transportation time for each route segment t_s is incorporated as Supplementary Equation (3):

$$m_{\mathrm{CO_2,prop,s}} = m_{\mathrm{CO_2,prop}} \cdot t_s \tag{3}$$

The total propulsion CO_2 emissions for the entire voyage ($m_{\mathrm{CO_2,prop,v}}$) is obtained by summing over all segments in Supplementary Equation (4):

$$m_{\mathrm{CO_2,prop,v}} = m_{\mathrm{CO_2,prop}} \cdot \sum_{s} t_s \tag{4}$$

The results are shown in Supplementary Table 4, and segment- and voyage-level values of t_s are presented in Supplementary Data 1 (excel file).

Supplementary Table 4. Propulsion-related CO_2 emissions

Routes	MCR (kW)	$\sum_s t_s$ (days)	FC_{prop} (tonne·day^{-1})	$m_{\mathrm{CO_2,prop}}$ (tonne·day^{-1})	$m_{\mathrm{CO_2,prop,v}}$ (tonne)
FEW 3	68640	88	224.0	694.5	61118.4
FEW 6	66220	55	216.1	670.0	36852.2
TP17 Eastbound	57100	44	186.4	577.8	25421.5
AE11 Westbound	75570	49	246.7	764.6	37467.7
AE7 Eastbound	54960	57	179.4	556.1	31698.1
AE7 Westbound	54960	47	179.4	556.1	26137.0
TP17 Westbound	68640	50	224.0	694.5	34726.3
TP2 Eastbound	72240	22	235.8	731.0	16081.0
TP2 Westbound	72240	42	235.8	731.0	30700.0
IA7	16080	35	52.5	162.7	5694.6
IA8	57100	42	186.4	577.8	24265.9
ME Eastbound	36560	29	119.3	369.9	10727.9
ME Westbound	36560	22	119.3	369.9	8138.4
IA1	36560	42	119.3	369.9	15537.0
IA88	13500	35	44.1	136.6	4780.9
PH5	21660	33	70.7	219.2	7232.4

Supplementary Methods 3. Energy requirement for CO_2 conditioning

Conditioning pathways considered

In this study, the energy requirement associated with preparing captured CO_2 for downstream handling is collectively referred to as CO_2 conditioning. Depending on the regeneration configuration, conditioning leads to different thermodynamic end states, reflecting the distinct storage and transport requirements of onboard and onshore regeneration schemes.

Two representative CO_2 conditioning pathways are considered. For onboard regeneration, CO_2 is conditioned to a liquid state at 20.4 bar and −28.9 ℃ and stored in dedicated onboard tanks. This condition ensures high volumetric density and safe, compact storage during ship operation.[24] For onshore regeneration, CO_2 is conditioned to a compressed liquid state at 120 bar and 25 ℃, suitable for pipeline transport. At this pressure–temperature combination, CO_2 remains in a single liquid phase throughout transportation, ensuring stable flow and avoiding two-phase operation.[25]

Conditioning energy for onboard regeneration (20.4 bar, −28.9 °C)

For the onboard regeneration scheme, CO_2 conditioning consists of multistage compression followed by low-temperature cooling, resulting in fully liquefied CO_2. The desorbed CO_2 stream (99 wt% CO_2) from the stripper overhead is compressed to 20.4 bar using multistage compression with intercooling. Aspen Plus simulations yield a specific compression energy of 73.9 kWh per tonne of CO_2. To reach the target storage temperature of −28.9 °C at 20.4 bar, an additional refrigeration step is required. Aspen Plus simulations give a cooling duty of −95.98 kW for the CO_2 stream entering the refrigeration system.

The refrigeration electricity requirement is calculated using the coefficient of performance (COP) formula, which is estimated as a function of temperature using Supplementary Fig. 5 and Supplementary Equation (5):

$$COP = 2.35 \times 10^{-4} e^{3.327\times 10^{-2} T} \tag{5}$$

At −28.9°C, the estimated COP is 0.79. The refrigeration energy requirement is then calculated using Supplementary Equation (6):

$$\text{Electricity required for refrigeration } = \frac{95.98}{(COP \times 99\ wt\%)} \tag{6}$$

The resulting refrigeration electricity requirement is 122.6 kWh per tonne of CO_2. Therefore, the total electricity required for onboard CO_2 conditioning is 196.5 kWh·tonne^{-1} CO_2, equivalent to 0.71 GJ·tonne^{-1} CO_2.

Conditioning energy for onshore regeneration (120 bar, 25 °C)

For the onshore regeneration scheme, CO_2 conditioning is performed at the onshore facility. The desorbed CO_2 is compressed to 120 bar at 25 °C, where it exists as a single-phase compressed liquid suitable for pipeline transport. The multistage compression is simulated in Aspen Plus, giving a specific electricity consumption of 119.8 kWh·tonne^{-1} of CO_2, equivalent to 0.43 GJ·tonne^{-1} CO_2. No low-temperature refrigeration is required for this pathway.

Comparison of conditioning energy requirements

Although both pathways produce liquid-phase CO_2, their energy requirements differ. Onboard conditioning requires both compression and refrigeration, whereas onshore conditioning requires compression only. In addition, onshore conditioning can use land-based electricity, which is assumed to have lower cost and carbon intensity than onboard power generation. These differences contribute to the energy and cost advantages of the onshore regeneration scheme.

Supplementary Methods 4. Regeneration heat estimation for different solvents

The regeneration heat (RE) represents the thermal energy required to release absorbed CO_2 from the solvent during solvent regeneration. In this study, RE values are estimated for four solvents: monoethanolamine (MEA), piperazine (PZ), the water-lean solvent NE-2 and a deep eutectic solvent (DES). The estimation procedures differ among solvents depending on the availability of literature data and the level of process information reported.

Regeneration heat of aqueous amine solvents

For aqueous MEA and PZ solutions, the RE values are estimated using a surrogate model adapted for post-combustion CO_2 capture. The model accounts for feed pressure, CO_2 molar fraction in the feed gas, capture rate, and has been widely applied in techno-economic assessments of solvent-based carbon capture systems.[26] In this study, the model is applied to ship exhaust gas containing 4.8 mol% CO_2 at a feed pressure of 1 bar and a capture rate of 90%. The model parameters—including the sensitivity coefficient (α), weighting factor (β) and base value of steam consumption ($f_{STM,0}$)—are listed in Supplementary Table 5.

Supplementary Table 5. Parameter of surrogate model for regeneration heat of monoethanolamine and piperazine

Solvent	Parameters					
	P_{FEED} (bar)	$x_{\mathrm{CO_2}}$	r_{CAP}	α	β	$f_{STM,0}$ (GJ·tonne^{-1} CO_2)
MEA	1.0	4.8 mol%	90%	−0.5659	0.16	2.45
PZ	1.0	4.8 mol%	90%	−0.0672	0.40	2.86

The regeneration heat is calculated according to Supplementary Equation (7):

$$RE = \alpha \ln[P_{\mathrm{FEED}} \cdot x_{\mathrm{CO_2}} \cdot (1 - r_{\mathrm{CAP}})^{\beta}] + f_{\mathrm{STM,0}} \tag{7}$$

where P_{FEED} is the feed pressure, $x_{\mathrm{CO_2}}$ is the CO_2 molar fraction in the feed gas, r_{CAP} is the capture rate, α is the sensitivity coefficient, β is the weighting factor and $f_{\mathrm{STM,0}}$ is the base steam consumption. The resulting RE values for MEA and PZ under ship-exhaust conditions are summarized in Supplementary Table 6.

Supplementary Table 6. Regeneration heat values of monoethanolamine and piperazine under ship-exhaust conditions

Solvent	RE (GJ·tonne^{-1} CO_2)
MEA	4.38
PZ	3.12

Regeneration heat of the water-lean solvent

For the water-lean solvent NE-2, ship-specific regeneration heat data are not available. A literature-reported minimum regeneration heat of 2.2 GJ·tonne^{-1} CO_2 is therefore used as the reference value. In the same reference, the regeneration heat of MEA is reported as 3.8 GJ·tonne^{-1} CO_2.[27] To account for the lower CO_2 partial pressure in ship exhaust relative, we apply a first-order correction using MEA as the benchmark solvent. Specifically, the ratio between the regeneration heat of MEA under ship-exhaust conditions in this study and that under the reference conditions is used as a penalty factor. This correction gives an estimated regeneration heat of 2.54 GJ·tonne^{-1} CO_2 for NE-2 under ship-exhaust conditions.

Regeneration heat of the deep eutectic solvent

The deep eutectic solvent (DES) considered in this study is 60wt% aqueous [MEACl][EDA] with a molar ratio of 1:6. This solvent is denoted as DES in the following sections. Because complete pilot-scale data and total regeneration heat are not available for this solvent, a literature-informed estimation framework is used. The total regeneration energy is estimated as the sum of the sensible heat, reaction heat, and latent heat contributions, as shown in Supplementary Equation (8):[28]

$$Q_{\text{reg}} \approx Q_{\text{sens}} + Q_{\text{reac}} + Q_{\text{lat}} \tag{8}$$

The sensible heat is determined from the specific solvent requirement and the solvent heat capacity. The heat capacity is estimated using pseudo-critical properties calculated with Lee-Kesler mixing rules based on the mole fractions and critical properties of each component in the aqueous DES.[29] This gives an estimated capacity of 6.9 kJ kg^{-1} K^{-1} and a sensible heat contribution of 1.298 GJ·tonne^{-1} CO_2.

The reaction heat is evaluated from the CO_2 absorption enthalpy. Based on the reported correlations between CO_2 uptake and heat of absorption for comparable deep eutectic solvent systems compositions, a CO_2 uptake of 32.15 wt% corresponds to an absorption enthalpy of approximately −57 kJ·mol^{-1}.[30] This gives a reaction heat contribution of 1.295 GJ·tonne^{-1} CO_2.

The latent heat contribution is estimated from the ratio of water vapor to CO_2 partial pressures at the stripper top,[30] as shown in Supplementary Equation (9):

$$Q_{\mathrm{lat}} = \frac{h_w}{M_{\mathrm{CO_2}}} \cdot \frac{P_w}{P_{\mathrm{CO_2}}} \tag{9}$$

where h_w is the latent heat of water, $M_{\mathrm{CO_2}}$ is the molar mass of CO_2, P_w is the water vapour pressure and $P_{\mathrm{CO_2}}$ is the CO_2 patrial pressure. The water vapor pressure is calculated from the water mole fraction and the saturated vapor pressure at the regeneration temperature, giving a latent heat contribution of about 0.51 GJ·tonne^{-1} CO_2.

The total regeneration energy of DES is therefore estimated to be approximately 3.10 GJ·tonne^{-1} CO_2. Applying the same ship-exhaust correction for NE-2 gives a corrected regeneration heat of 3.57 GJ·tonne^{-1} CO_2. The final RE values for NE-2 and DES under ship-exhaust conditions are summarized in Supplementary Table 7.

Supplementary Table 7. Regeneration heat values of water-lean solvent and the deep eutectic solvent under ship-exhaust conditions

Solvent	RE (GJ·tonne^{-1} CO_2)
NE-2	2.54
DES	3.57

Supplementary Methods 5. Cost of regeneration and conditioning for different regeneration schemes

The costs of solvent regeneration and CO_2 conditioning are calculated separately for the onboard and onshore regeneration schemes because their energy sources and emission-accounting boundaries differ.

For onboard regeneration, the total daily CO_2 emissions consist of emissions from ship propulsion and additional emissions generated by the regeneration and conditioning processes. This relationship is expressed as Supplementary Equation (10):

$$m_{\mathrm{CO_2}} = m_{\mathrm{CO_2,prop}} + m_{\mathrm{CO_2,add}} \tag{10}$$

where $m_{\mathrm{CO_2}}$ is the total daily CO_2 emission from ship, $m_{\mathrm{CO_2,prop}}$ is the daily CO_2 emission from ship propulsion, and $m_{\mathrm{CO_2,add}}$ is the additional daily CO_2 emissions caused by onboard solvent regeneration and CO_2 conditioning.

For onshore regeneration, all regeneration and conditioning processes occur on land. These processes still consume energy and generate CO_2 emissions, but the associated emissions are attributed to the on-land system. Therefore, no additional CO_2 emissions are counted onboard, and $m_{\mathrm{CO_2,add}} = 0$ for the onshore regeneration scheme. These land-based emissions are not included in the capture cost per tonne of CO_2, but they are included in the carbon tax and total cost calculations.

Onboard regeneration and conditioning cost

For the onboard regeneration scheme, the additional CO_2 emissions associated with solvent regeneration and conditioning are calculated using Supplementary Equation (11):

$$m_{\mathrm{CO_2,add}} = (RE \times BFC + CE \times EFC) \times CR_s \cdot \left(m_{\mathrm{CO_2,prop}} + m_{\mathrm{CO_2,add}}\right) \times EF \tag{11}$$

where RE and CE represent the regeneration and conditioning energy requirements per tonne of CO_2, CR_s is the segment level CO_2 capture rate, BFC is the boiler fuel consumption per unit energy output, EFC is the specific fuel oil consumption (SFOC) of the marine diesel generator ($183 \mathrm{g \cdot kWh^{-1}}$)[22], and EF is the fuel's CO_2 emission factor. BFC is estimated based on the lower heating value (LHV) of the fuel ($42.7\ \mathrm{MJ \cdot kg^{-1}}$) and a boiler thermal efficiency of 90%, resulting in $0.026\ \mathrm{tonne \cdot GJ^{-1}}$.

To simplify the calculation of additional CO_2 emissions, a solvent-specific coefficient for additional carbon emissions (CACE) is defined as Supplementary Equation (12):

$$CACE = (RE \times BFC + CE \times EFC) \times EF \tag{12}$$

The CACE values for different solvents are summarized in Supplementary Table 8.

Supplementary Table 8. Coefficients for additional carbon emissions for different solvents

Solvent	RE (GJ·tonne^{-1} CO_2)	CE (GJ·tonne^{-1} CO_2)	$CACE$
MEA	4.38	0.71	0.465
PZ	3.12	0.71	0.363
NE-2	2.54	0.71	0.316
DES	3.57	0.71	0.400

Combining Supplementary Equation (11) and Supplementary Equation (12) gives Supplementary Equation (13):

$$m_{\mathrm{CO_2,add}} = \frac{CACE \times CR_s \times m_{\mathrm{CO_2,prop}}}{1 - CACE \times CR_s} \tag{13}$$

Taking MEA as an example, the additional CO_2 emissions is calculated using Supplementary Equation (14):

$$m_{\mathrm{CO_2,add,MEA}} = \frac{0.465 \cdot CR_s \cdot m_{\mathrm{CO_2,prop}}}{1 - 0.465 \cdot CR_s} \tag{14}$$

Given the carbon emission coefficients, the corresponding additional daily fuel consumption can be determined as Supplementary Equation (15).

$$FC_{\mathrm{add}} = \frac{m_{\mathrm{CO_2,add}}}{EF} \tag{15}$$

The cost of onboard regeneration and conditioning per tonne of captured CO_2 is then calculated as Supplementary Equation (16):

$$C_{\mathrm{regeneration,onboard}} + C_{\mathrm{conditioning,onboard}} = \frac{FC_{\mathrm{add}} \times Fuel\ price}{m_{\mathrm{CO_2}} \cdot CR_s} \tag{16}$$

Heavy fuel oil (HFO), commonly used in shipping, includes various fuel types, making it difficult to determine a single representative price. Thus, in this study, the price of HFO is set to US$500 per tonne, estimated based on the price of IFO 380—a widely used variant of HFO.[31]

By combining Supplementary Equation (11), (12), (15), and (16), the cost of onboard regeneration and conditioning per tonne of captured CO_2 can be expressed as Supplementary Equation (17):

$$C_{\mathrm{regeneration,onboard}} + C_{\mathrm{conditioning,onboard}} = \frac{CACE \times Fuel\ price}{EF} \tag{17}$$

This expression shows that the onboard regeneration and conditioning cost per tonne of captured CO_2 is independent of the total daily CO_2 emission and the segment-level capture rate. Instead, it is determined by the solvent-specific CACE value, the fuel price and the fuel emission factor. The resulting costs are summarized in Supplementary Table 9.

Supplementary Table 9. Cost of onboard regeneration and conditioning per tonne of CO_2 captured (US\$ tonne^{-1} CO_2)

Solvent	$C_{\text{regeneration,onboard}} + C_{\text{conditioning,onboard}}$ (US\$ tonne^{-1} CO_2)
MEA	74.92
PZ	58.59
NE-2	50.95
DES	64.41

Onshore regeneration and conditioning cost

The cost of onshore solvent regeneration is estimated based on the standard price of low-pressure steam, and the cost of CO_2 conditioning is calculated using the electricity price of a typical onshore plant. The electricity price is assumed to be US\$ 0.0775 kWh^{-1}, equivalent to US\$ 21.5 GJ^{-1}. The low-pressure steam price is taken as US\$ 1.9 GJ^{-1}.

The cost of solvent regeneration and CO_2 conditioning per tonne of captured CO_2 for onshore regeneration scheme is calculated as Supplementary Equation (18):

$$C_{\text{regeneration,onshore}} + C_{\text{conditioning,onshore}} = RE \times LP + CE \times EP \qquad (18)$$

where RE is the regeneration heat requirement, CE is the CO_2 conditioning energy requirement, LP is the low-pressure steam price, and EP is the electricity price. The resulting costs are summarized in Supplementary Table 10.

Supplementary Table 10. Cost of onshore regeneration and conditioning per tonne of CO_2 captured (US\$ · tonne^{-1} CO_2)

Solvent	RE (GJ·t^{-1} CO_2)	CE (GJ·t^{-1} CO_2)	$C_{\text{regeneration,onshore}} + C_{\text{conditioning,onshore}}$ (US\$ tonne^{-1} CO_2)
MEA	4.38	0.43	17.57
PZ	3.12	0.43	15.18
NE-2	2.54	0.43	14.06
DES	3.57	0.43	16.03

Supplementary Methods 6. Rich-solvent weight ratio

The rich-solvent weight ratio (λ) is used to estimate the rich-solvent weight contributing to the OCCS load and cargo loss cost for the onshore regeneration scheme.

Amine solutions (MEA and PZ) are prepared as 30 wt% aqueous solutions. Based on the CO_2 loading (mol CO_2 mol^{-1} amine) and the molecular weights of the solvent and CO_2, the rich-solvent weight ratio for MEA and PZ is calculated as Supplementary Equation (19):

$$\lambda_{\text{amine}} = \frac{\frac{Molecular\ weight\ of\ amine}{CO_2\ loading \times 44}}{Solvent\ concentration\ wt\%} + 1 \tag{19}$$

For NE-2, the highest reported CO_2 absorption capacity (γ) is 1.78 mol CO_2 L^{-1}.[27] Since the exact density of the NE-2 solution (containing water) is unknown, it is assumed to be approximately equal to that of water. Using this assumption, the rich-solvent weight ratio is estimated as Supplementary Equation (20):

$$\lambda_{\text{NE-2}} = \frac{1000}{\gamma \times 44} + 1 \tag{20}$$

For DES, the calculation is based on CO_2 uptake in weight percentage (wt%). The rich-solvent weight ratio for DES is calculated using Supplementary Equation (21):

$$\lambda_{\text{DES}} = \frac{1}{CO_2\ uptake(wt\%)} + 1 \tag{21}$$

The resulting values of λ for the four different solvents evaluated in this study are summarized in Supplementary Table 11.

Supplementary Table 11. Rich-solvent weight ratios of different solvents

Solvent	Molecular weight (g·mol^{-1})	CO_2 loading	λ (tonne rich-solvent tonne^{-1} CO_2)
MEA[32]	61.1	0.5 mol CO_2 mol^{-1} amine	10.3
PZ[32]	86.1	1.06 mol CO_2 mol^{-1} amine	7.2
NE-2[27]	106.4	1.78 mol CO_2 liter^{-1} solution	13.8
DES[33]	65.5	32.15 wt% CO_2 uptake	4.11

Supplementary Methods 7. Ratio of onboard carbon capture and storage load to ship net tonnage for the onboard regeneration scheme at 90% capture rate

The OCCS load for onboard regeneration is calculated by considering the weight of captured CO_2 and the weight of the CO_2 storage tank. The segment-level OCCS load and the maximum segment-level OCCS load are calculated using the Supplementary Equation (22) and (23):

$$m_{\mathrm{s,onboard}} = m_{\mathrm{tank}} + m_{\mathrm{CO_2}} \cdot t_s \cdot CR_s \quad (22)$$

$$m_{\mathrm{s,max}} = m_{\mathrm{tank}} + m_{\mathrm{CO_2}} \cdot t_{\mathrm{s,max}} \cdot CR_s \quad (23)$$

where $m_{\mathrm{s,onboard}}$ is the OCCS load for segment s, $m_{\mathrm{s,max}}$ is the maximum segment-level OCCS load across the voyage, m_{tank} is the CO_2 storage tank weight, $m_{\mathrm{CO_2}}$ is the total daily CO_2 emission from the ship, t_s is the duration of segment s, $t_{\mathrm{s,max}}$ is the duration of the longest segment in the voyage, and CR_s is the segment-level CO_2 capture rate.

Weight of captured CO_2

At a 90% capture rate (CR_s = 90%), the additional CO_2 emissions ($m_{\mathrm{CO_2,add}}$) are calculated using Supplementary Equation (13), and the results are provided in Supplementary Data 1. By combining these values with the propulsion-related CO_2 emissions ($m_{\mathrm{CO_2,prop}}$) from Supplementary Table 4, the total daily CO_2 emissions onboard ($m_{\mathrm{CO_2}}$) can be obtained, as shown in Supplementary Data 1. Based on the daily value, segment-level results for each route can be calculated. Taking the AE11 Westbound route as an example, with a 90% system capture rate using MEA, the CO_2 emissions are summarized in Supplementary Table 12.

Supplementary Table 12. CO_2 emissions for route AE11 Westbound at 90% capture rate using monoethanolamine for the onboard regeneration scheme

Port	t_s (days)	$m_{\mathrm{CO_2,prop,s}}$ (tonne)	$m_{\mathrm{CO_2,add}} \cdot t_s$ (tonne)	$m_{\mathrm{CO_2}} \cdot t_s$ (tonne)
Qingdao	-	0	0	0
Busan	3	2293.9	1648.0	3942.0
Ningbo	5	3823.2	2746.7	6569.9
Shanghai	3	2293.9	1648.0	3942.0
Xiamen	4	3058.6	2197.4	5256.0
Nansha New Port	1	764.6	549.3	1314.0
Yantian	1	764.6	549.3	1314.0
Singapore	5	3823.2	2746.7	6569.9
Port Said East	15	11469.7	8240.1	19709.8
Gioia Tauro	4	3058.6	2197.4	5256.0
Barcelona	4	3058.6	2197.4	5256.0
Valencia	4	3058.6	2197.4	5256.0
Total	49	37467.7	26917.7	64385.4

The corresponding data for all routes using different solvents under the onboard regeneration scheme are provided in Supplementary Data 1.

Weight of the CO_2 storage tank

The tank size is determined by the maximum amount of CO_2 captured between two ports—that is the longest segment within a single voyage. For example, on the AE11 Westbound route, the longest segment spans 15 days—Singapore to Port Said East. CO_2 is stored onboard in liquid form at −28.9°C and 20.4 bar, which requires a thick-walled storage tank. Given the density of liquefied CO_2 (1.073 tonne·m^{-3}), the required tank volume for each route can be calculated as Supplementary Equation (24):

$$V_{\mathrm{CO_2,max}} = \frac{m_{\mathrm{CO_2}} \cdot t_{\mathrm{s,max}} \cdot CR_s}{\rho_{\mathrm{CO_2}}} \tag{24}$$

To ensure a practical tank design that fits within the ship's structure, the CO_2 storage tank is assumed to be in the cargo area, with its longer side oriented perpendicular to the ship's longitudinal axis. Assuming that the tank's length is 2 meters shorter than the ship's width, the tank volume is calculated as Supplementary Equation (25):

$$V_{\mathrm{tank}} = \frac{\pi}{4} D^2 \cdot H + \left(\frac{\pi}{24} D^3 + \frac{\pi}{4} D^2 \cdot h\right) \times 2 \tag{25}$$

Let the tank volume equal to the required maximum CO_2 storage volume using Supplementary Equation (26):

$$V_{\mathrm{CO_2,max}} = V_{\mathrm{tank}} \tag{26}$$

D and H can be solved. Then, the tank weight is calculated by Supplementary Equation (27):

$$m_{\mathrm{tank}} = 7.9 \times d \times (0.69\pi D^2 + 2\pi D h + \pi D H) \tag{27}$$

where d is the tank wall thickness (m), D is the tank diameter (m), and h is the straight-edge height of the tank head (mm). When the tank diameter is greater than 2 meters, h is set to 40 mm; when the diameter is less than 2 meters, h is set to 25 mm. The tank is made of stainless steel with a density of 7.9 tonne·m^{-3}.

Taking the AE11 Westbound route as an example, under a 90% CO_2 capture rate using MEA at the onboard regeneration scheme, the ship has a width of 61.5 meters, and the tank length is assumed to be 59.5 meters (2 meters shorter than the ship's width). The storage tank is designed to accommodate a maximum CO_2 mass of 17738.8 tonnes, which corresponds to a required storage volume of 16532.0 m^3. Based on the above equations, the resulting tank diameter is 17.9 meters, and the total tank weight is calculated to be 5067.1 tonnes.

The corresponding storage tank specifications for different solvents at 90% capture rate are provided in Supplementary Data 1.

Now, the m_s and $m_{\mathrm{s,max}}$ can be determined by Supplementary Equation (22) and (23). The OCCS-load-To-ship-net-tonnage ratio ω_s per segment is given by Supplementary Equation (28):

$$\omega_s = \frac{m_s}{net\ tonnage} \tag{28}$$

The maximum OCCS-load-To-ship-net-tonnage ratio $\omega_{\max}$ across the voyage is Supplementary Equation (29):

$$\omega_{\max} = \frac{m_{\mathrm{s,max}}}{net\ tonnage} \tag{29}$$

For the AE11 Westbound route under a 90% capture rate using the MEA onboard regeneration scheme, $\omega_{\max}$ is 0.17, while the average ratio across the voyage is 0.07, as shown in Supplementary Table 13.

Supplementary Table 13. Ratio of onboard carbon capture and storage load to ship net tonnage for route AE11 Westbound at 90% capture rate using the monoethanolamine onboard regeneration scheme

Port	$m_{\mathrm{CO_2}} \cdot t_s \cdot CR_s$ per segment (tonne)	$m_{\mathrm{s,onboard}}$ per segment (tonne)	ω_s
Qingdao	0	0	0.00
Busan	3547.8	8614.9	0.06
Ningbo	5912.9	10980.1	0.08
Shanghai	3547.8	8614.9	0.06
Xiamen	4730.4	9797.5	0.07
Nansha New Port	1182.6	6249.7	0.05
Yantian	1182.6	6249.7	0.05
Singapore	5912.9	10980.1	0.08
Port Said East	17738.8	22806.0	0.17
Gioia Tauro	4730.4	9797.5	0.07
Barcelona	4730.4	9797.5	0.07
Valencia	4730.4	9797.5	0.07
Average/voyage	57946.9	113685.4	0.07

The data of ω_s for all routes are provided in Supplementary Data 1. The maximum and average OCCS-load to ship-net-tonnage ratios for onboard regeneration using different solvents are also summarized in Supplementary Data 1.

Supplementary Methods 8. Ratio of onboard carbon capture and storage load to ship net tonnage for the onshore regeneration scheme at 90% capture rate

For the onshore regeneration scheme, the OCCS is mainly determined by the mass of CO_2-rich solvent stored onboard before offloading at port. The segment-level OCCS is calculated as Supplementary Equation (30).

$$m_{\mathrm{s,onshore}} = m_{\mathrm{CO_2}} \cdot \lambda \cdot t_s \cdot CR_s \tag{30}$$

where $m_{\mathrm{CO_2}} = m_{\mathrm{CO_2,prop}}$ is the daily CO_2 emissions (Supplementary Table 4), and λ is the rich-solvent weight ratio (Supplementary Table 11).

Taking the IA88 route using MEA as an example, the ω_s and $\omega_{\max}$ is calculated using Eqs. 31 and 32 and shown in Supplementary Table 14. As shown, $\omega_{\max}$ reaches 0.81, while the average value is 0.32. Detailed results of ω_s for the onshore regeneration scheme at 90% capture rate are provided in Supplementary Data 2.

Supplementary Table 14. Ratio of onboard carbon capture and storage load to ship net tonnage for route IA88 at 90% capture rate using the monoethanolamine-based onshore regeneration scheme

Port	$m_{\mathrm{CO_2}}$ (tonne·day^{-1})	$m_{\mathrm{s,onshore}}$ (tonne)	ω_s
Yokohama		0	
Nagoya	136.6	1260.7	0.16
Osaka	136.6	1260.7	0.16
Kobe	136.6	1260.7	0.16
Moji	136.6	1260.7	0.16
Hibiki Nada	136.6	1260.7	0.16
Busan	136.6	1260.7	0.16
Gwangyang	136.6	1260.7	0.16
Shanghai	409.8	3782.0	0.49
Ningbo	136.6	1260.7	0.16
Ho Chi Minh City	683.0	6303.4	0.81
Laem Chabang	546.4	5042.7	0.65
Bangkok	273.2	2521.4	0.33
Laem Chabang	136.6	1260.7	0.16
Haiphong	683.0	6303.4	0.81
Hong Kong	273.2	2521.4	0.33
Nansha New Port	0.0	0.0	0.00
Yokohama	683.0	6303.4	0.81
Average/voyage	–	2595.5	0.32

The maximum and average OCCS-load to ship-net-tonnage ratios for the onshore regeneration scheme are summarized in Supplementary Data 2.

Supplementary Methods 9. Fixed equipment cost for the onboard regeneration scheme

The capital cost of the absorber and associated fixed equipment is primarily determined by the absorber design. The absorber is sized explicitly using solvent-specific kinetic and equilibrium properties. We first estimate the relevant physical and transport properties of each solvent (viscosity, density, diffusivity, surface tension). These properties are used with standard packed-column design correlations to calculate gas-liquid friction factors, mass-transfer coefficients, pressure drop, and the height of a transfer unit (HTU).

Given the flue-gas flow rate and target capture efficiency, we determine the required number of transfer units (NTU) and apply overall gas–liquid material balances to obtain the necessary absorber cross-sectional area and packed height. The absorber diameter and total height follow directly from these sizing steps. The absorber cost is then estimated using standard chemical-process equipment cost correlations based on the resulting dimensions.

The absorber cost is used as the primary sizing basis for estimating the total bare-equipment cost (BEC) of the onboard OCCS system. The BEC is converted to an installed inside-battery-limits (ISBL) cost using a standard installation factor and further adjusted to obtain the fixed-capital investment (FCI) by including indirect costs such as engineering, construction, and contingencies. The FCI is then annualised using a capital-recovery factor, and the annual equipment cost is divided by the annual captured CO_2 to yield the specific equipment cost in USD per tonne of CO_2 captured.

The detailed calculation methods are provided below.

Solvent viscosity and density

For each solvent (30 wt% MEA, 30 wt% PZ, NE-2 and 60wt% DES), the temperature-dependent liquid viscosity and density used in the absorber-sizing model are listed in Supplementary Table 15.

Supplementary Table 15. Temperature-dependent viscosity and density of the solvents

	Viscosity (mPa·s)			Density (kg·m^{-3})		
T(K)	298	313	323	298	313	323
			Before CO_2 capture			
MEA[34]	2.48	1.67	1.33	1010.6	1003.4	998.1
PZ[35]	5.336	3.346	2.028	1074.9	1065.5	1054.8
NE-2[36]	12	7	5	1018	1005	999
DES[33]	11.41	6.054	4.253	1022.4	1011.8	1004.6
			After CO_2 capture			
MEA[34]	3.9	2.7	2.1	1121.1	1114	1108
PZ[35]	6.010	3.751	2.502	1127.8	1118.8	1108.1
NE-2[36]	24	15	8	1070	1060	1050
DES[33]	200.6	92.3	57.8	1261.3	1253.9	1248.9

CO_2 diffusivity in the liquid phase

The diffusivity of CO_2 in the liquid phase, D_L, is calculated from solvent viscosity using the standard correlation[37], as shown in Supplementary Equation (31):

$$D_L = D_{CO_2,w(T)} \cdot \left(\frac{\mu_{w(T)}}{\mu_{mix(T)}}\right)^o \tag{31}$$

where $D_{CO_2,w(T)} = 2.35 \times 10^{-6} \cdot \exp\left(-\frac{2119}{T}\right)$ is the diffusivity of CO_2 in water, $\mu_{w(T)}$ is the viscosity of pure water,[38] $\mu_{mix(T)}$ is the viscosity of the solvent mixture (MEA, PZ, NE-2 or DES), and o = 0.6 is a commonly used exponent for aqueous amine systems.

Surface tension

Surface tension values for the four solvents are assigned based on representative data for similar solvent classes. For 30 wt% MEA and 30 wt% PZ, we assume a surface tension equal to that of water over the relevant temperature range, 0.07 $N \cdot m^{-1}$.[38] For NE-2 an intermediate value of 0.05 $N \cdot m^{-1}$ is used, reflecting its behaviour between aqueous solutions (0.07 $N \cdot m^{-1}$) and ionic-liquid-like solvents (0.03–0.04 $N \cdot m^{-1}$).[39] For DES, a surface tension of 0.04 $N \cdot m^{-1}$ is adopted, consistent with reported values of deep eutectic solvents.[39] The surface tensions of solvents are summarized in Supplementary Table 16.

Supplementary Table 16. Surface tension values used for the solvents

Surface tension	MEA	PZ	NE-2	DES
σ ($N \cdot m^{-1}$)	0.07	0.07	0.05	0.04

Gas-phase properties

The gas-phase diffusivity of CO_2 was taken as a representative constant for CO_2-air diffusion[38], as shown in Supplementary Equation (32):

$$D_G = 1.6 \times 10^{-5}\ m^2 \cdot s^{-1} \tag{32}$$

Gas-phase density and viscosity were set to values of air at atmospheric pressure and moderate temperature[38], as shown in Supplementary Equation (33) and (34):

$$\rho_G = 1.2\ kg \cdot m^{-3} \tag{33}$$

$$\mu_G = 1.8 \times 10^{-5}\ \text{Pa} \cdot \text{s} \tag{34}$$

Mass balance and solvent loading

In this section we establish the relationship between the route-specific flue-gas conditions and the design variables used in packed absorber sizing. Starting from the inlet flue-gas flow

rate, CO_2 load and target capture rate, we determine the required solvent circulation rate and the corresponding lean and rich loadings. These relations follow well-established absorber design methodology.[40]

Gas-phase CO_2 balance

For HTU/NTU analysis, the gas-phase composition is expressed as the molar ratio of CO_2 to-non-CO_2 components of the flue gas, as shown in Supplementary Equation (35) and (36):

$$Y_u = \frac{n_{\mathrm{CO_2,in}}}{n_{\mathrm{non\ CO_2}}} \tag{35}$$

$$Y_o = \frac{n_{\mathrm{CO_2,out}}}{n_{\mathrm{non\ CO_2}}} \tag{36}$$

where Y_u and Y_o are the inlet and outlet CO_2 ratios (kmol CO_2 per kmol non-CO_2 flue gas). Here (Supplementary Equation (37)),

$$n_{\mathrm{non\ CO_2}} = n_{\mathrm{gas,in}} - n_{\mathrm{CO_2,in}} \tag{37}$$

with $n_{\mathrm{gas,in}}$ being the total inlet flue-gas flow (kmol·h^{-1}).

The outlet CO_2 flow is determined from the column capture rate CR_{col} = 90%, as shown in Supplementary Equation (38):

$$n_{\mathrm{CO_2,out}} = n_{\mathrm{CO_2,in}} - C_{\mathrm{cap}} = (1 - CR_{\mathrm{col}}) \cdot n_{\mathrm{CO_2,in}} \tag{38}$$

The total outlet gas flow can be obtained as Supplementary Equation (39):

$$n_{\mathrm{gas,out}} = n_{\mathrm{non\ CO_2}} + n_{\mathrm{CO_2,out}} \tag{39}$$

Treatment of design flue-gas flow rate

The flue-gas flow rate strongly influences absorber diameter and capital cost. Under normal conditions, the absorber is designed to treat the full flue-gas flow so that the system achieves the maximum possible capture rate whenever the solvent load limit allows.

However, for certain routes, the maximum achievable capture rate for all segments along the voyage (due to load-limit constraints) is lower than the intrinsic capture capacity of the absorber (CR_{col} = 90%). Designing for the full flue-gas flow in such cases would oversize the absorber, because capture would stop once the load limit is reached, leaving unused capacity.

A more cost-effective strategy is to size the absorber for a reduced design flow rate and bypass the remainder of the flue gas during operation. This achieves the same overall voyage-level capture performance without unnecessary absorber oversizing. For routes where this applies, a reduced design flow of 88% of the full flue-gas flow is adopted.

Minimum solvent flow and rich loading $X_{u,m}$

A global CO_2 balance over the absorber gives the rich loading at the top of the column for operation at the minimum solvent circulation (Supplementary Equation (40)):

$$X_{u,m} = X_0 + \frac{n_{\text{non CO}_2}(Y_u - Y_o)}{L_m} = X_0 + \frac{n_{\text{non CO}_2}(Y_u - Y_o)\phi}{L_0} \tag{40}$$

where:

L_0 is the actual solvent circulation rate,

L_m is the minimum solvent flow, and

ϕ is the design solvent-to-minimum ratio.

The minimum flow is related to the design flow by Supplementary Equation (41):

$$L_m = \frac{L_0}{\phi} \tag{41}$$

A value of $\phi = 1.4$ (i.e., 40% above minimum) is used following conventional absorber design practice.

Approximate equilibrium slope at the lean end

To couple gas–liquid mass transfer in the HTU formulation, the equilibrium line near the lean end is approximated as a straight line through the origin. Its slope is determined from the lean-end point $(X_{u,m}, Y_u)$ (Supplementary Equation (42)):

$$m_{\text{eq}} = \frac{Y_u}{X_{u,m}} \tag{42}$$

This slope is used to compute the overall gas-phase height of a transfer unit (Supplementary Equation (43)):

$$\text{HTU}_{\text{OV}} = H_G + m_{\text{eq}} H_L \tag{43}$$

where H_G and H_L are the gas- and liquid-side HTUs derived from standard mass-transfer correlations.

Conversion to mass flows for hydraulics

For hydraulic calculations and pressure-drop estimation,[41] molar flows are converted to mass flows as shown in Supplementary Equation (44) and (45):

$$\dot{m}_G = n_{\text{non CO}_2} MW_{\text{air}} + n_{\text{CO}_2,\text{out}} MW_{\text{CO}_2} \tag{44}$$

$$\dot{m}_L = L_0 MW_{\text{liq}} \tag{45}$$

where MW_{air} and MW_{CO_2} are the molecular weights of air and CO_2, and MW_{liq} is the effective molecular weight of the solvent.

Absorber sizing procedure

The packed absorber is sized using a height-of-a-transfer-unit (HTU/NTU) framework combined with structured-packing hydraulic correlations. All sizing calculations are performed for each route and each solvent-temperature condition.

Packing properties

In this study, Mellapak 250Y is selected as the representative structured packing for the absorber.[21,42] The geometrical parameters required for hydraulic and mass-transfer calculations are listed in Supplementary Table 17.[43]

Supplementary Table 17. Mellapak 250Y constant geometrical parameters[43]

Parameter	Symbol	Value
Specific surface area[44]	a_t	250 ($m^2 \cdot m^{-3}$)
Void fraction[44]	ε	0.97
Packing-specific constant loading[43]	C_s	3.157
Packing-specific constant[43]	$C_{P,0}$	0.292
Corrugation base width	B	0.012 (m)
Corrugation height	h_p	0.0125 (m)
Corrugation spacing	S	0.020 (m)

Diameter from pressure-drop constraint

For each route, the overall gas and liquid mass flow rates $\dot{m}_G$ and $\dot{m}_L$ are obtained from the material balance section. For a trial column diameter D, the cross-sectional area is calculated by Supplementary Equation (46):

$$A_{\mathrm{col}} = \frac{\pi D^2}{4} \tag{46}$$

The superficial gas and liquid velocities are calculated by Supplementary Equation (47) and (48):

$$U_{gs} = \frac{\dot{m}_G}{\rho_G A_{\mathrm{col}}} \tag{47}$$

$$U_{ls} = \frac{\dot{m}_L}{\rho_L A_{\mathrm{col}}} \tag{48}$$

The effective gas and liquid velocities inside the corrugation channels are[40] calculated by Supplementary Equation (49) and (50):

$$U_{ge} = \frac{U_{gs}}{\varepsilon sin\theta} \tag{49}$$

$$U_{le} = \frac{3\Gamma}{2\rho_L}(\frac{{\rho_L}^2 g}{3\mu_L \Gamma})^{0.33} \tag{50}$$

where $\Gamma = \frac{L'}{P}$ is the liquid line load; $L' = \frac{\dot{m}_L}{A_{\text{col}}}$ is the line liquid flux; $P = \frac{4S+B}{Bh_p}$ is the effective perimeter.

Billet–Schultes hydraulic model

Using the effective velocities, the Billet–Schultes correlation[43] gives the liquid holdup and pressure drop, as shown in Supplementary Equation (51).

$$\frac{\Delta P}{L} = \frac{\psi_L a_t}{2K(\varepsilon - h_L)^3}(u_{ge}\sqrt{\rho_G})^2 \tag{51}$$

The packing parameter K is as 1 by $\frac{1}{K} = 1 + \frac{2}{3}(1-\varepsilon)\frac{(6\frac{(1-\varepsilon)}{a_t})}{D}$.

where the resistance coefficient of two-phase flow is calculated using Supplementary Equation (52):

$$\psi_L = \psi_{\text{dry}} \exp\left(C_1\sqrt{Fr_L}\right) \tag{52}$$

the resistance coefficient on the gas side is calculated as Supplementary Equation (53):

$$\psi_{\text{dry}} = C_{p,0}\left(\frac{64}{Re_g} + \frac{1.8}{Re_g^{0.08}}\right) \tag{53}$$

with the gas Reynolds number (Supplementary Equation (54)):

$$Re_g = \frac{\rho_G u_{ge} d_e}{\mu_G} \tag{54}$$

and liquid Froude number (Supplementary Equation (55)):

$$Fr_L = \frac{u_{le}^2 a_t}{g} \tag{55}$$

The packing constant (Supplementary Equation (56)):

$$C_1 = \frac{13300}{a_t^{1.5}} \tag{56}$$

where liquid holdup can be obtained from (Supplementary Equation (57), (58), (59) and (60)):

$$h_L \approx h_{\text{static}} + 0.20 Re_l^{0.10} Fr_l^{0.20} \tag{57}$$

$$h_{\text{static}} = 0.01 \tag{58}$$

$$Re_l = \frac{\rho_L U_{ls}}{a_t \mu_L} \tag{59}$$

$$Fr_l = \frac{u_{ls}^2 a_t}{g} \tag{60}$$

Diameter selection

A bisection search is used to determine the required absorber diameter D_{req} such that the calculated pressure drop satisfies the design limit: $\frac{\Delta P}{L}$ is just below the design limit (here 800 Pa·m^{-1}).[41] The resulting absorber diameter D_{req}, A_{col}, U_{gs} and U_{ls} are then used for the mass-transfer and HTU/NTU calculations.

Local mass-transfer coefficients and height of a transfer unit

With A_{col}, $\dot{m}_G$ and $\dot{m}_L$ determined, the local mass-transfer coefficients and height of a transfer unit (HTU) are computed as follows.:

Effective wetted area

The Onda correlation is used to estimate the effective wetted area[45] (Supplementary Equation (61)):

$$a_e = a_t \left[1 - \exp\left(-1.45 \left(\frac{\sigma_c}{\sigma}\right)^{0.75} {Re_l}^{*0.1} Fr_l^{*-0.05} We_l^{0.2}\right]\right. \tag{61}$$

where the dimensionless groups are (Supplementary Equation (62), (63) and (64)):

$$Re_l^{\,*} = \frac{L'}{a_t \mu_L} \tag{62}$$

$$Fr_l^{\,*} = \frac{L' a_t}{{\rho_L}^2 g} \tag{63}$$

$$We_l = \frac{L'}{\rho_L \sigma a_t} \tag{64}$$

Gas-film coefficient

An equivalent hydraulic diameter d_e (Supplementary Equation (65))is calculated from the packing geometry.[46]

$$d_e = Bh_p \left(\frac{1}{B + 2S} + \frac{1}{2S}\right) \tag{65}$$

The gas-film mass-transfer coefficient is then obtained from[40] (Supplementary Equation (66)):

$$k_g = 0.0338 \cdot \frac{D_G}{d_e} ({Re_g}^*)^{0.8} (Sc_g)^{0.33} \tag{66}$$

where ${Re_g}^*$ and Sc_g (Supplementary Equation (67) and (68)):

$$Re_g^{\,*} = \frac{d_e \rho_G}{\mu_G} \left(u_{ge} + u_{le}\right) \tag{67}$$

$$Sc_g = \frac{\mu_G}{\rho_G D_G} \tag{68}$$

Liquid-film coefficient

The liquid-side coefficient is evaluated using[40](Supplementary Equation (69)):

$$k_l = 2\sqrt{\frac{D_L U_{le}}{\pi S}} \tag{69}$$

Height of transfer units

The gas-side and liquid-side HTUs are calculated as[46](Supplementary Equation (70) and (71)):

$$H_G = \frac{\rho_G U_{gs}}{k_g a_e} \tag{70}$$

$$H_L = \frac{L'}{k_l a_e \rho_L} = \frac{(\dot{m}_L / A_{\text{col}})}{k_l a_e \rho_L} \tag{71}$$

The overall gas-phase HTU is then calculated as Supplementary Equation (72):

$$\text{HTU}_{\text{OV}} = H_G + m_{\text{eq}} H_L \tag{72}$$

Overall number of transfer units from linearized operating line

The absorber is treated as an approximately linear counter-current stage. The overall number of transfer units (NTU) is then evaluated as[47] Supplementary Equation (73):

$$\text{NTU}_{\text{OG}} = \frac{1}{A_{df}} \ln\left(\frac{A_{df} Y_u + \lambda_{cr} Y_o - m_{\text{eq}} X_o}{A_{df} Y_o + \lambda_{cr} Y_o - m_{\text{eq}} X_o}\right) \tag{73}$$

where λ_{cr} and A_{df} (Supplementary Equation (74) and (75)):

$$\lambda_{cr} = m_{\text{eq}} \frac{n_{\text{non CO}_2}}{\dot{n}_{\text{L,in}}} \tag{74}$$

$$A_{df} = 1 - \lambda_{cr} \tag{75}$$

Packed height, segmenting, and design margin

Once the column diameter and HTU–NTU relationships are established, the required packed height is obtained from Supplementary Equation (76):

$$Z = \text{HTU}_{\text{OV}} \cdot \text{NTU}_{\text{OG}} \tag{76}$$

To account for mechanical constraints and maldistribution, the packing is divided into segments of maximum length Z_{seg} (here 6 m).[48] The number of required segments and

redistributors is calculated as Supplementary Equation (77) and (78):

$$n_{\text{seg}} = \frac{Z}{Z_{\text{seg}}} \tag{77}$$

$$n_{\text{redist}} = \max(0, n_{\text{seg}} - 1) \tag{78}$$

The structural packed height (Supplementary Equation (79)) then becomes:

$$Z_{\text{total}} = Z + n_{\text{redist}} \cdot H_{\text{redist}} \tag{79}$$

where $H_{\text{redist}} = 0.5m$ is the height of each liquid redistributor.

The final design height for each route (Supplementary Equation (80)) is taken as the maximum Z_{total} over all evaluated conditions multiplied by a safety factor (here 1.2):

$$Z_{\text{design}} = 1.2 \max_{T} Z_{\text{total}}(T) \tag{80}$$

Together, the diameter D_{req} from the hydraulic constraint and the height Z_{design} fully define the absorber dimensions used in the techno-economic assessment.

Absorber thickness, weight, and cost

The wall thickness of the carbon-steel column shell is estimated using a standard mechanical-design correlation (diameter in m, thickness in m) as shown in Supplementary Equation (81):

$$t(m) = 0.003573 \times D_{\text{req}} + 0.005 \tag{81}$$

The steel density is taken as $\rho_{\text{steel}} = 7990\text{kg} \cdot \text{m}^{-3}$.

The bare steel weight of the column shell (including an allowance for stiffeners, nozzles and ellipsoidal heads) is calculated as Supplementary Equation (82):

$$W_{\text{vessel}} = \pi D_{\text{req}} \cdot Z_{\text{design}} \cdot t(m) \cdot \rho_{\text{steel}} + \frac{\pi {D_{\text{req}}}^2 \cdot t(m) \cdot \rho_{\text{steel}}}{2} \tag{82}$$

The bare-equipment cost of the column shell is then estimated from an empirical correlation[49] (Supplementary Equation (83)):

$$C_{\text{absorber}} = -10000 + {W_{\text{vessel}}}^{0.6} \times 600 \tag{83}$$

Total bare-equipment cost of the onboard system

For the onboard regeneration scheme, the absorber represents 4.58% ($F_{\text{equip,onboard}}$) of the total onboard equipment cost in the reference design (Supplementary Table 18).

Supplementary Table 18. Cost breakdown of the organic Rankine cycle-integrated onboard CO_2 capture system[42]

Equipment category	Equipment	Purchase cost (Totally: 3.24×10^6 US$)	Breakdown of cost (excluding ORC and LNG cold energy reuse)[a]
Column	Absorber	97112.52	4.58%
	Stripper	75420.72	3.55%
	Packing	28032.48	1.32%
	Trays	133488	6.29%
Heat exchanger	H1	155675.52	7.34%
	H2	162751.68	7.67%
	H3	148599.36	7.00%
	H4	185749.2	8.75%
	H5	203439.6	9.59%
	H6	150368.4	Excluded in our system
	H7	150368.4	Excluded in our system
	H8	238820.4	Excluded in our system
	Reboiler	373267.44	17.59%
Compressor and pump	C1	225309.6	10.62%
	C2	192067.2	9.05%
	P1	22161.6	1.04%
	P2	21546	Excluded in our system
	P3	43707.6	2.06%
	P4	31395.6	1.48%
	P5	22161.6	1.04%
	P6	21546	1.02%
	P7	35089.2	Excluded in our system
Turbine	Turbine	521640	Excluded in our system

Therefore, the total bare-equipment cost of all onboard equipment is obtained by scaling, as shown in Supplementary Equation (84):

$$C_{\text{equip,onboard,total}} = \frac{C_{\text{absorber}}}{4.58\%} \tag{84}$$

Installed capital expenditure and annualized cost

Inside-battery-limits (ISBL) cost

An installation factor *f* (accounting for foundations, piping, instrumentation, etc.) is applied

[a] ORC and LNG are not incorporated in our ship-based carbon capture system, thus the corresponding equipment cost should be excluded from calculation.

to obtain the inside-battery-limits (ISBL) cost[49], as shown in Supplementary Equation (85):

$$ISBL_{onboard} = C_{\text{equip,onboard,total}} \cdot f \tag{85}$$

where $f = 4$.

Fixed-capital investment (FCI)

Indirect costs (engineering, construction, and contractor's fees) are represented by a 14% increment as shown in Supplementary Equation (86):

$$FCI_{onboard} = ISBL_{onboard} \cdot (1 + 0.14) \tag{86}$$

Total capital expenditure (CAPEX)

To incorporate start-up costs, contingencies, and working capital, the FCI is converted to total CAPEX using a standard factor of 0.8 (Supplementary Equation (87)):

$$CAPEX_{onboard} = \frac{FCI_{onboard}}{0.8} \tag{87}$$

Annualized capital expenditure

The annualised $\text{CAPEX}_{\text{y,onboard}}$ is calculated using a standard capital-recovery factor (CRF), assuming a plant lifetime $t = 25$ years and a discount rate $i = 8\%$ as shown in Supplementary Equation (88):

$$CAPEX_{y,onboard} = CAPEX_{onboard} \cdot \frac{i \cdot (i+1)^t}{(i+1)^t - 1} \tag{88}$$

Specific annual equipment cost

The specific equipment cost per tonne of captured CO_2 is then calculated as Supplementary Equation (89):

$$C_{\text{equip,onboard}} = \frac{CAPEX_{y,onboard}}{m_{\text{CO}_2\text{,annual}}} \tag{89}$$

where $m_{\text{CO}_2\text{,annual}}$ is the annual mass of CO_2 captured by the ship, assuming 300 operating days per year.

The resulting specific annual equipment costs for each solvent and route are summarised in Supplementary Data 4.

Supplementary Methods 10. Fixed equipment cost for the onshore regeneration scheme

In the onshore regeneration scheme, only the absorber is installed onboard, while all regeneration, heat-exchange, compression, and conditioning equipment are located at the port-side facility. Accordingly, the fixed-equipment cost of the onshore configuration consists of two components, the onboard-side equipment cost and the onshore-side equipment cost, as shown in Supplementary Equation (90):

$$C_{\text{equip,onshore}} = C_{\text{equip,onshore,onboard-side}} + C_{\text{equip,onshore,onshore-side}} \tag{90}$$

Onboard-side equipment cost

For onshore scheme, the absorber on the ship is sized using the same procedure described in Supplementary Methods 9. Moreover, the packing inside the absorber remains essential, and its cost is explicitly included. The packing volume is calculated using Supplementary Equation (91):

$$V_{\text{Packing}} = Z_{\text{design}} \cdot \frac{\pi {D_{\text{req}}}^2}{4} \tag{91}$$

The bare-equipment cost of structured packing is then estimated using a standard unit cost[49] as shown in Supplementary Equation (92):

$$C_{\text{Packing}} = V_{\text{Packing}} \cdot 3200 \tag{92}$$

The onboard-side equipment cost is then calculated using Supplementary Equation (93):

$$C_{\text{equip,onshore,Absorber}} = C_{\text{absorber}} + C_{\text{packing}} \tag{93}$$

Conversion of the absorber equipment cost to ISBL cost, fixed-capital investment (FCI), total CAPEX, and annualized capital cost follows the same methodology described in Supplementary Methods 9. The specific onboard-side equipment cost per tonne of CO_2 captured is calculated using Supplementary Equation (94):

$$C_{\text{equip,onshore,onboard-side}} = \frac{CAPEX_{y,onshore}}{m_{\text{CO}_2\text{,annual}}} \tag{94}$$

Onshore-side equipment cost

The cost of this onshore regeneration equipment depends on the solvent system and is estimated to use published CAPEX data for land-based post-combustion capture systems. All costs are expressed per tonne of CO_2 captured.

Monoethanolamine

The cost for MEA is estimated from a land-based carbon capture process (Supplementary Table 19).[50]

Supplementary Table 19. Equipment cost of a land-based carbon capture process

Equipment	Capital cost (US$ tonne^{-1} CO_2)
Capital cost of heat exchanger	4.02
Other capital cost (excluding absorber and heat exchanger)	3.79

The resulting onshore-side equipment cost for MEA is given in Supplementary Equation (95):

$$C_{\text{equip,onshore-side,MEA}} = 7.81\ (\text{US\$ tonne}^{-1}\ \text{CO}_2) \tag{95}$$

This value serves as the baseline for scaling the onshore costs of other solvents.

Piperazine

PZ shares the same process configuration as MEA in the reference NGCC post-combustion capture plant.[51] To estimate this cost for PZ, we compute the ratio of total direct costs (TDC) excluding the absorber, as shown in Supplementary Equation (96):

$$k_{\text{PZ}} = \frac{TDC_{\text{PZ,no absorber}}}{TDC_{\text{MEA,no absorber}}} = \frac{15.40}{24.48} = 0.63 \tag{96}$$

The onshore facility cost for PZ is then calculated as Supplementary Equation (97):

$$C_{\text{equip,onshore-side,PZ}} = k_{\text{PZ}} \cdot C_{\text{equip,onshore-side,MEA}} = 4.91\ (\text{US\$ tonne}^{-1}\ \text{CO}_2) \tag{97}$$

Water-lean solvent

For the water-lean solvent NE-2, a detailed equipment-cost breakdown is not reported. However, comparative CAPEX data for the major process units of MEA and NE-2 are available.[28] We first remove the absorber and then compute the overall NE-2/MEA cost ratio for the remaining units as Supplementary Equation (98) and (99):

$$CAPEX_{\text{NE-2,no absorber}} = 77.9\ (M\$) \tag{98}$$

$$CAPEX_{\text{MEA,no absorber}} = 92.9\ (M\$) \tag{99}$$

The scaling ratio is therefore calculated as Supplementary Equation (100):

$$k_{\text{NE-2}} = \frac{CAPEX_{\text{NE-2,no absorber}}}{CAPEX_{\text{MEA,no absorber}}} = 0.84 \tag{100}$$

Thus, the result is shown in Supplementary Equation (101):

$$C_{\text{equip,onshore-side,NE-2}} = k_{\text{NE-2}} \cdot C_{\text{equip,onshore-side,MEA}} = 6.55\ (\text{US\$ tonne}^{-1}\ \text{CO}_2) \tag{101}$$

Deep eutectic solvent

For the DES solvent, no complete total annualized cost (TAC) or detailed equipment-cost breakdown is available in the literature. The equipment cost was therefore estimated based on the similarity of DES to conventional amine systems. Specifically, the regeneration heat duty of DES is comparable to that of MEA, indicating similar reboiler duty, sizing, and cost, while its low volatility resembles that of PZ, suggesting that the remaining regeneration-train equipment (e.g., stripper shell, condensers, heat exchangers, tanks, and pumps) can be reasonably scaled using the corresponding PZ-based process as a reference.[28,51]

To capture both effects, we decompose the MEA and PZ onshore equipment into reboiler and non-reboiler portions, as shown in Supplementary Equation (102) and (103):

$$f_{\mathrm{reb,PZ}} = \frac{C_{\mathrm{reb,onshore,PZ}}}{TDC_{\mathrm{PZ,no\ absorber}}} \tag{102}$$

$$f_{\mathrm{reb,MEA}} = \frac{C_{\mathrm{reb,onshore,MEA}}}{CAPEX_{\mathrm{MEA,no\ absorber}}} \tag{103}$$

The DES onshore cost is then estimated by hybrid scaling, as shown in Supplementary Equation (104):

$$\begin{aligned} C_{\mathrm{equip,onshore-side,DES}} = C_{\mathrm{equip,onshore-side,PZ}} \cdot \left(1 - f_{\mathrm{reb,PZ}}\right) \\ + C_{\mathrm{equip,onshore-side,MEA}} \cdot f_{\mathrm{reb,MEA}} \end{aligned} \tag{104}$$

Applying this scaling yields, as shown in Supplementary Equation (105):

$$C_{\mathrm{equip,onshore-side,DES}} = 6.18\ (\mathrm{US\$} \cdot \mathrm{tonne}^{-1}\ \mathrm{CO_2}) \tag{105}$$

The resulting fixed-equipment costs for all solvents under the onshore regeneration scheme are summarized in Supplementary Data 4.

Supplementary Methods 11. Transportation cost

The transportation cost for each route is estimated by averaging the historical freight rates, and then converted using Supplementary Equation (106):

$$Transportation\ cost\ (\mathrm{US\$} \cdot \mathrm{tonne}^{-1}) = \frac{Freight\ rate(US\$ \cdot FEU^{-1})}{30.48\ (tonne \cdot FEU^{-1})} \qquad (106)$$

Supplementary Table 20. Freight rates and transportation cost

Routes	Average freight rates (US$·FEU^{-1}*)	Transportation cost (US$·tonne^{-1})
FEW 3	801	26.3
FEW 6	801	26.3
TP17 Eastbound	1222.5	40.1
AE11 Westbound	1000.5	32.8
AE7 Eastbound	1000.5	32.8
AE7 Westbound	1000.5	32.8
TP17 Westbound	1222.5	40.1
TP2 Eastbound	973.5	31.9
TP2 Westbound	973.5	31.9
IA7	621.5	20.4
IA8	621.5	20.4
ME Eastbound	695.5	22.8
ME Westbound	695.5	22.8
IA1	621.5	20.4
IA88	621.5	20.4
PH5	621.5	20.4

* FEU: Forty-foot equivalent unit

Supplementary Methods 12. Solvent cost

Solvent costs arise from the initial solvent inventory required for system operation and the make-up solvent required to compensate for solvent loss during operation. The total solvent cost for the onboard and onshore regeneration schemes are calculated using Supplementary Equation (107) and (108), respectively.

$$C_{\text{solvent,onboard}} = C_{\text{initial solvent,onboard}} + C_{\text{make-up}} \quad (107)$$

$$C_{\text{solvent,onshore}} = C_{\text{initial solvent,onshore}} + C_{\text{make-up}} \quad (108)$$

Solvent price assumptions

Solvent price is an important input for estimating solvent-related costs. In this study, the adopted solvent prices are selected to reflect current industrial availability, production maturity, and anticipated scaling behavior, rather than laboratory-scale costs. The prices of MEA and PZ are obtained from previous studies.[52,53] For the water-lean solvent NE-2 and the deep eutectic solvent (DES), no established large-scale market prices are currently available. Rather than using laboratory-scale synthesis costs, which would unrealistically inflate solvent-cost impacts, we assume that upon industrial deployment their bulk production costs would be comparable to that of PZ, reflecting similar synthesis complexity and chemical functionality. This assumption is intended as a placeholder for future industrial-scale pricing, ensuring consistency across solvent comparisons while avoiding speculative cost inflation. The adopted values are summarized in Supplementary Table 21.

Supplementary Table 21. Solvent price (US$·tonne^{-1})

Solvent	Price (US$·tonne^{-1})
MEA	1250
PZ	8000
NE-2	8000
DES	8000

Initial solvent inventory cost

Unlike fixed equipment, the initial solvent inventory is a one-off consumable with no salvage value and is not financed as capital, so its cost is simply spread evenly over the plant lifetime rather than annualized with a capital-recovery factor.

For the onboard regeneration scheme, the ship must initially be charged with sufficient

solvent to fill the liquid holdup of both the absorber and stripper columns, as well as to provide an additional working inventory corresponding to the circulating solvent. The stripper volume is assumed to be identical to that of the absorber. In addition, a liquid residence time of 0.5 h in the circulation loop is included to ensure stable operation. Consistent with previous OCCS studies, the entire solvent inventory is assumed to be discarded and replaced together with the capture plant at the end of the vessel lifetime, which is taken as 25 years.[49,54] Accordingly, the initial solvent cost per tonne of CO_2 captured for onboard regeneration scheme is calculated as Supplementary Equation (109):

$$C_{\text{initial solvent,onboard}} = \frac{(\rho_L \times h_L \times A_{\text{col}} \times 2 + \dot{m}_L \times 0.5) \cdot p_{\text{solv}}}{25 \times m_{\text{CO}_2,\text{annual}}} \tag{109}$$

where ρ_L is the solvent density, h_L is liquid hold-up of absorber, $\dot{m}_L$ is the solvent mass flow rate, p_{solv} is the solvent price (US$·tonne^{-1}) and $m_{\text{CO}_2,\text{annual}}$ is the annual CO_2 captured by the ship.

For the onshore regeneration scheme, the absorber remains installed onboard, while solvent regeneration is performed at port using shore-based facilities. For each route, the initial solvent inventory is therefore set to the amount of solvent needed to operate the absorber continuously over the longest voyage segment without offloading or regeneration. Because this working solvent inventory circulates within a global shore-based regeneration network rather than being permanently assigned to a single vessel, it is not discarded at the end of ship life. Instead, we assume an annual solvent loss rate of 1% of this initial inventory. Therefore, the initial solvent cost per tonne of CO_2 captured for onshore regeneration scheme is given by Supplementary Equation (110):

$$C_{\text{initial solvent,onshore}} = \frac{m_{\text{s,max}} \times \frac{\lambda - 1}{\lambda} c_{\text{solu}} \cdot p_{\text{solv}}}{m_{\text{CO}_2,\text{annual}}} \times 1\% \tag{110}$$

where $m_{\text{s,max}}$ is OCCS load of the segment with the maximum transportation days (tonne), λ is rich-solvent weight ratio (tonne rich-solvent tonne^{-1} CO_2) and c_{solu} is the solvent concentration.

It should be noted that the initial solvent inventory cost varies with the applied load limit, because different load limits lead to different annual captured CO_2 amounts, ($m_{\text{CO}_2,\text{annual}}$), in Supplementary Equations (109) and (110). The results of initial solvent cost per tonne of CO_2 captured are summarized in the Supplementary Data 5.

Make-up solvent cost

During operation, solvents are inevitably lost through degradation, oxidation, evaporation, and leakage.[55] These losses are accounted for as a make-up solvent cost (US$ per tonne of CO_2 captured) and are estimated based on reported literature values together with solvent-specific physicochemical properties.

For MEA, pilot-plant data report a solvent loss of about 1.6 kg MEA per tonne of CO_2 captured.[55] The make-up cost is therefore calculated as Supplementary Equation (111)

$$C_{\text{make-up,MEA}} = \frac{1.6}{1000} \times p_{\text{solv,MEA}} = 2\ US\$ \cdot tonne^{-1}\ CO_2 \text{ captured} \tag{111}$$

where $p_{\text{solv,MEA}}$ is the MEA price (1250 US$·tonne^{-1}).

For PZ, owing to its lower volatility and higher oxidative stability relative to MEA, the make-up solvent cost of PZ is approximated as one half of that for MEA.[51] The make-up solvent cost of PZ is calculated using Supplementary Equation (112):

$$C_{\text{make-up,PZ}} \approx 0.5 \times C_{\text{make-up,MEA}} = 1\ US\$ \cdot tonne^{-1}\ CO_2 \text{ captured} \tag{112}$$

For NE-2, pilot-plant data comparing MEA and NE-type solvents indicate that the amine make-up cost for the NE process is about twice that of MEA.[28] The make-up solvent cost of NE-2 is therefore calculated using Supplementary Equation (113):

$$C_{\text{make-up,NE-2}} \approx 2 \times C_{\text{make-up,MEA}} = 4\ US\$ \cdot tonne^{-1}\ CO_2 \text{ captured} \tag{113}$$

For DES, no pilot-scale data on solvent loss under cyclic CO_2 absorption–desorption conditions are currently available. However, DESs are generally characterized by negligible vapor pressure and low volatility, suggesting that solvent losses due to evaporation are expected to be lower than for conventional aqueous amines. In the absence of quantitative loss data, we adopt a conservative assumption that the make-up solvent cost of DES is equal to that of MEA, as shown in Supplementary Equation (114):

$$C_{\text{make-up,DES}} = C_{\text{make-up,MEA}} = 2\ US\$ \cdot tonne^{-1}\ CO_2 \text{ captured} \tag{114}$$

The results of solvent cost per tonne of CO_2 captured for onboard and onshore regeneration scheme using different solvents are summarized in Supplementary Data 5.

Supplementary Methods 13. Capture cost for the onboard regeneration scheme

Capture cost for the onboard regeneration scheme (CC_{onboard}) is calculated by Supplementary Equation (115):

$$CC_{\text{onboard}} = \frac{C_{\text{OCCS,onboard}}}{OCR \cdot M_{\text{CO}_2}} = C_{\text{equip,onboard}} + C_{\text{regeneration,onboard}} + C_{\text{conditioning,onboard}} + C_{\text{cargo loss,onboard}} + C_{\text{solvent,onboard}} \quad (115)$$

The fixed equipment cost is calculated in Supplementary Methods 9. The cost of regeneration and conditioning are calculated in Supplementary Methods 5, and the cost of solvent is calculated in Supplementary Methods 12. The cargo loss cost requires additional treatment because it depends on the relationship between the ω_{s}, ω_{max} and ω_{limit}. In the calculation of the OCCS-load-to-ship-net-tonnage ratio, the segment capture rate (CR_s) is initially assumed to be 90%. However, this assumption may no longer hold once a load limit is applied.

Case where the maximum load ratio is below the load limit ($\boldsymbol{\omega_{\text{max}} < \omega_{\text{limit}}}$)

In this case, the entire flue gas stream from the ship is processed by the OCCS system. Therefore, the segment capture rate (CR_s) and the Overall capture rate (OCR) both remain at 90%. The corresponding CO_2 emissions values and the storage tank information are provided in Supplementary Data 1.

Case where the maximum load ratio exceeds the load limit ($\boldsymbol{\omega_{max} > \omega_{limit}}$)

At this circumstance, at least one segment's OCCS load exceeds the load limit. For segments where $\omega_s < \omega_{\text{limit}}$, the CR_s is still 90%. However, for segments where $\omega_s > \omega_{\text{limit}}$, the segment CR_s cannot reach 90% and must be recalculated. The OCCS load for such segments is constrained by the load limit and is given by Supplementary Equation (116):

$$m_{\text{s,onboard}} = \omega_{\text{limit}} \cdot net\ tonnage = m_{\text{tank}} + m_{\text{s,captured}} \quad (116)$$

As a result, the amount of CO_2 captured is reduced, leading to a smaller required tank volume. Under different ω_{limit} conditions, the tank should be pre-designed to accommodate the maximum capture requirement, which occurs at $t_{\text{s,max}}$. To achieve this, m_{tank} and $m_{\text{s,captured}}$ are determined by simultaneously solving Supplementary Equation (25), (27), (116) (117) and (118).

$$V_{\text{tank}} = V_{\text{s,captured}} \quad (117)$$

$$V_{\text{s,captured}} = \frac{\omega_{\text{limit}} \cdot net\ tonnage - m_{\text{tank}}}{\rho_{\text{CO}_2}} = \frac{m_{\text{s,captured}}}{\rho_{\text{CO}_2}} \quad (118)$$

The results for tank dimensions under different load limits are presented in Supplementary Data 1.

Recalculation of segment-level and voyage-level capture rates

Then, the segment capture rate can be recalculated using Supplementary Equation (119):

$$CR_s = \frac{m_{\mathrm{s,captured}}}{m_{\mathrm{CO_2}} \cdot t_s} \quad (119)$$

The segment capture rates CR_s for all routes are provided in Supplementary Data 1 (excel file).

Having the CR_s, the voyage-level overall capture rate (OCR) is calculated as Supplementary Equation (120):

$$OCR = \frac{\sum_s t_s \cdot m_{\mathrm{CO_2}} \cdot CR_s}{M_{\mathrm{CO_2}}} \quad (120)$$

where the total CO_2 emissions over the voyage is given by Supplementary Equation (121):

$$M_{\mathrm{CO_2}} = \sum_s t_s \cdot m_{\mathrm{CO_2}} \quad (121)$$

The results of OCR are presented in Supplementary Data 1.

Cargo loss cost

The cost associated with cargo loss is calculated at the segment level using Supplementary Equation (122):

$$C_{\mathrm{s,cargo\ loss,onboard}} = \left(Transportation\ price \cdot \frac{t_s}{\sum_s t_s}\right) \cdot m_{\mathrm{s,onboard}} \quad (122)$$

The cost of cargo loss per tonne of captured CO_2 is calculated as Supplementary Equation (123):

$$C_{\mathrm{cargo\ loss,onboard}} = \frac{\sum_s C_{\mathrm{s,cargo\ loss,onboard}}}{OCR \cdot M_{\mathrm{CO_2}}} \quad (123)$$

The results of cargo loss costs per tonne CO_2 ($C_{\mathrm{cargo\ loss,onboard}}$) using different solvents under various load limits are presented in Supplementary Data 6.

Final capture cost

Based on the calculated costs for cargo loss, solvent, fixed equipment, and regeneration and conditioning costs, the capture cost (CC_{onboard}) is then determined by Supplementary Equation (115). Supplementary Data 6 summarizes the results.

Supplementary Methods 14. Capture cost for the onshore regeneration scheme

Capture cost for the onshore regeneration scheme (CC_{onshore}) is calculated using Supplementary Equation (124):

$$CC_{\text{onshore}} = \frac{C_{\text{OCCS,onshore}}}{OCR \cdot M_{\text{CO}_2}} = C_{\text{equip,onshore}} + C_{\text{regeneration,onshore}} + C_{\text{conditioning,onshore}} + C_{\text{cargo loss,onshore}} + C_{\text{solvent,onshore}} \quad (124)$$

The fixed equipment cost is calculated in Supplementary Methods 10. The regeneration and conditioning costs are calculated in Supplementary Methods 5, and the cost of solvent is calculated in Supplementary Methods 12. Similar to the onboard regeneration scheme, different scenarios must be considered when calculating the cargo loss cost. However, the calculation is much simpler because the mass of storage tank is not included in the onshore regeneration case. Additionally, based on our calculation, ω_{max} for all routes are larger than the three selected ω_{limit}. Therefore, only the relationship between ω_s and ω_{limit} is discussed.

Case where the segment-level load ratio is below the load limit ($\omega_s < \omega_{\text{limit}}$)

In this case, the segment capture rate (CR_s) is fixed at 90%. The corresponding values of m_{CO_2} can be obtained from Supplementary Data 2.

Case where the segment-level load ratio exceeds the load limit ($\omega_s > \omega_{limit}$)

The segment capture rate CR_s is constrained by the available cargo capacity. The OCCS load is determined using Supplementary Equation (125):

$$m_{\text{s,onshore}} = \omega_{\text{limit}} \cdot net\ tonnage = m_{\text{CO}_2} \cdot CR_s \cdot \lambda \cdot t_s \quad (125)$$

The segment capture rate is then recalculated as Supplementary Equation (126):

$$CR_s = \frac{\omega_{\text{limit}} \cdot net\ tonnage}{\lambda \cdot m_{\text{CO}_2} \cdot t_s} \quad (126)$$

The voyage-level overall capture rate (OCR) and total CO_2 emissions over the voyage are calculated using the same method as the onboard scheme, as shown in Supplementary Equation (120) and (121). The resulting overall capture rates are provided in Supplementary Data 2.

Cargo loss cost

The cost of cargo loss per segment is calculated using Supplementary Equation (127):

$$C_{\text{s,cargo loss,onshore}} = \left(Transportation\ price \cdot \frac{t_s}{\sum_s t_s}\right) \cdot m_{\text{s,onshore}} \quad (127)$$

The cost of cargo loss per tonne of captured CO_2 is calculated as Supplementary Equation (128):

$$C_{\text{cargo loss,onshore}} = \frac{\sum_s C_{\text{s,cargo loss,onshore}}}{OCR \cdot M_{\text{CO}_2}} \tag{128}$$

The results for $C_{\text{cargo loss,onshore}}$ using different solvents under various load limits are presented in Supplementary Data 6.

Final capture cost

Based on the calculated costs for cargo loss, solvent, fixed equipment, regeneration and conditioning, the capture cost (CC_{onshore}) is then determined using Supplementary Equation (124). The results are summarized in Supplementary Data 6.

Supplementary Methods 15. 2030–2050 Carbon taxes and emission factors

2030–2050 Carbon tax prediction

The global carbon tax is estimated based on the global CO_2 emissions under the "Net Zero Emissions by 2050" Scenario,[56,57] by considering each region's share of emissions (Supplementary Table 22) and their corresponding carbon tax (Supplementary Table 23). This weighted approach reflects regional emission disparities and provides a tax estimate suitable for global shipping applications. As a result, the projected global carbon taxes for 2030, 2040, and 2050 are US$ 102.2, US$ 168.8, and US$ 205.9 per tonne of CO_2, respectively.

Supplementary Table 22. Global CO_2 emission ratios under the "Net Zero Emissions by 2050" scenario

Region	2030	2040	2050
Advanced economies	0.244	0.195	0.117
Emerging markets and developing economies	0.756	0.805	0.883

Supplementary Table 23. Carbon tax estimates under the "Net Zero Emissions by 2050" scenario

Region	2030	2040	2050
Advanced economies	140.0	205.0	250.0
Emerging markets and developing economies	90.0	160.0	200.0
Global (US$·tonne^{-1} CO_2)	102.2	168.8	205.9

2030–2050 Emissions-trading-systems-equivalent carbon tax prediction

To explore how geographically heterogeneous carbon-pricing policies influence OCCS economics, we construct a route-dependent emissions trading systems (ETS)-equivalent carbon tax based on port locations and the regional CO_2 tax trajectories in the IEA Net Zero Emissions by 2050 scenario.

Under the IEA Net Zero Emissions scenario, the assumed CO_2 taxes (in US$ per tonne of CO_2) for 2030–2050 are in Supplementary Table 24.

Supplementary Table 24. Regional carbon tax projections under the "Net Zero Emissions by 2050" scenario

US$ per tonne of CO_2	2030	2040	2050
Advanced economies	140	205	250
Selected emerging market and developing economies with net zero emissions pledges	90	160	200
Other emerging market and developing economies without net zero emissions pledges	25	85	180

We then use these regional taxes as input for the ETS-equivalent calculation described below.

Each port p is mapped to its corresponding region $r(p)$; the port then inherits the regional CO_2 tax in year y, as shown in Supplementary Equation (129):

$$C_p(y) = C_{r(p)}(y) \tag{129}$$

A voyage on route r is divided into segments $s = 1, \ldots, N$, each linking two consecutive ports p_s^{start} and p_s^{end}. For each year y, the segment-level ETS-equivalent carbon tax is defined as Supplementary Equation (130):

$$C_{\text{t,route,s(y)}} = \frac{C_{p_s^{\text{start}}}(y) + C_{p_s^{\text{end}}}(y)}{2} \tag{130}$$

This segment-level value is used directly in the cost calculations for both onboard and onshore regeneration schemes, by multiplying $C_{\text{t,route,s(y)}}$ with the uncaptured CO_2 emitted during segment s. These intermediate values do not appear in summary tables, as they are embedded within the full cost model.

Additionally, for comparison of carbo tax intensity across routes, we compute an aggregated route-level ETS-equivalent carbon tax as a sailing-time-weighted average of the segment-level taxes (Supplementary Equation (131)).

$$C_{\text{t,route(y)}} = \frac{\sum_{s=1}^{N} C_{\text{t,route,s(y)}} \cdot t_s}{\sum_{s=1}^{N} t_s} \tag{131}$$

where t_s is the duration (days) of segment s. The resulting route-level ETS-equivalent carbon taxes for all selected routes are reported in Supplementary Table 25.

Supplementary Table 25. Route-level emissions trading systems equivalent carbon taxes (US$ per tonne CO_2)

Routes	2030	2040	2050
IA88	76.93	140.93	206.86
PH5	78.03	145.23	198.94
IA7	41.71	104.29	185.14
ME Eastbound	71.47	136.21	200.86
ME Westbound	62.84	126.93	196.36
IA1	71.90	138.21	196.90
IA8	80.95	147.14	203.81
TP17 Westbound	74.35	137.50	207.30
FEW 6	63.55	126.59	199.73
TP17 Eastbound	128.35	193.58	240.91
FEW 3	52.13	114.57	193.07
TP2 Westbound	121.55	188.39	231.55
TP2 Eastbound	107.05	175.34	217.05
AE7 Westbound	49.15	111.65	190.74
AE7 Eastbound	51.45	114.74	190.26
AE11 Westbound	83.11	147.91	209.18

2030–2050 emission factors prediction

For onshore regeneration schemes, carbon emissions are calculated using land-based emission factors, as both regeneration and conditioning occur on land. The process involves steam for regeneration and electricity for conditioning. Supplementary Table 26 summarizes the global electricity emission factors (EEF_G) for 2030, 2040, and 2050.[56]

Supplementary Table 26. Global electricity emission factor (g CO_2 kwh^{-1})

Year	2030	2040	2050
EEF_G	270	149.5	29

The global steam emission factors (SEF_G) are estimated for 2030–2050 by weighting regional steam emission factors (Supplementary Table 27)[58] with industrial energy consumption ratios (Supplementary Table 28)[59-61].

Supplementary Table 27. Regional steam emission factors (g CO_2 kwh^{-1})

Region	SEF 2030	SEF 2040	SEF 2050
EU	204.4	107.1	9.8
US	231.4	122.4	13.3
CN	286.4	151.0	15.6

Supplementary Table 28. Proportions of industrial energy consumption in different regions

Region	Industrial energy consumption (Terajoule)	IR
EU	13030539	17.30%
US	15046051	19.97%
CN	47260869	62.73%

SEF_G is estimated as Supplementary Equation (132):

$$SEF_G = IR_{EU} \times SEF_{EU} + IR_{US} \times SEF_{US} + IR_{CN} \times SEF_{CN} \quad (132)$$

The results are shown in Supplementary Table 29.

Supplementary Table 29. Global steam emission factors (g CO_2 kwh^{-1})

Year	2030	2040	2050
SEF_G	261.2	137.7	14.1

Finally, the onshore-regeneration emission factor is calculated using Supplementary Equation (133):

$$OEF = (RE \times SEF_G + CE \times EEF_G) \times EUF \quad (133)$$

where RE is the regeneration energy consumption (GJ·tonne^{-1} CO_2), SEF_G is the global steam emission factor (g CO_2 kwh^{-1}), CE is the conditioning energy consumption (GJ·tonne^{-1} CO_2), EEF_G is the global electricity emission factor (g CO_2·kwh^{-1}), and EUF is the energy unit transfer factor with a value of 2.78×10^{-4}.

The OEF for each solvent and year scenarios are summarized in Supplementary Table 30.

Supplementary Table 30. Onshore regeneration emission factors (tonne CO_2 emitted · tonne^{-1} CO_2 captured)

Year	2030	2040	2050
MEA	0.350	0.186	0.021
PZ	0.259	0.137	0.016
NE-2	0.216	0.115	0.013
DES	0.292	0.155	0.017

Supplementary Methods 16. Total cost with carbon tax for onboard regeneration

Total cost with predicted global carbon tax

For the onboard regeneration scheme, the total cost with carbon tax is calculated using Supplementary Equation (134):

$$TCT_{\text{onboard}} = C_{\text{OCCS,onboard}} + (1 - OCR) \cdot M_{\text{CO}_2} \cdot C_t \tag{134}$$

where C_t is the carbon tax.

The corresponding results under different routes, solvents, weight limits, and carbon tax scenarios (2030, 2040, 2050) are summarized in Supplementary Data 6.

Total cost with route-specific emissions-trading-systems-equivalent carbon tax

For the onboard regeneration scheme, the total cost with a route-specific ETS-equivalent carbon tax is calculated using Supplementary Equation (135):

$$TCT_{\text{onboard}} = C_{\text{OCCS,onboard}} + \sum_{s} t_s \cdot m_{\text{CO}_2}(1 - CR_s) \cdot C_{\text{t,route,s(y)}} \tag{135}$$

where t_s is the transport time of a segment, m_{CO_2} is the daily total CO_2 emissions, CR_s is the capture rate per segment and $C_{\text{t,route,s(y)}}$ is ETS-equivalent carbon tax of the segment at a specific year.

The results of the total cost using the route-specific ETS-equivalent carbon tax are shown in Supplementary Data 7.

Supplementary Methods 17. Total cost with carbon tax for onshore regeneration

Total cost with predicted global carbon tax

The total cost with carbon tax for the onshore regeneration scheme is calculated using Supplementary Equation (136):

$$TCT_{\text{onshore}} = C_{\text{OCCS,onshore}} + (1 - OCR) \cdot M_{\text{CO}_2} \cdot C_t + OEF \cdot OCR \cdot M_{\text{CO}_2} \cdot C_t \quad (136)$$

where C_t is the carbon tax (US\$·tonne^{-1} CO_2). OEF is the onshore-regeneration emission factor (Supplementary Table 30). The values of C_t, SEF_G, and EEF_G across 2030, 2040, and 2050 are summarized in Supplementary Table 31.

Supplementary Table 31. Global carbon tax and emission factors

	2030	2040	2050
Carbon tax C_t (\$/tonne CO_2)	102.2	168.8	205.9
SEF_G (g CO_2/kwh)	261.2	137.7	14.1
EEF_G (g CO_2/kwh)	270	149.5	29

The resulting total cost for onshore regeneration under various scenarios is summarized in Supplementary Data 6.

Total cost with route-specific emissions-trading-systems-equivalent carbon tax

For the onshore regeneration scheme, the total cost with a route-specific ETS-equivalent carbon tax is calculated using Supplementary Equation (137):

$$TCT_{\text{onshore}} = C_{\text{OCCS,onshore}} + \sum_{s} t_s \cdot \left(m_{\text{CO}_2}(1 - CR_s) \cdot C_{\text{t,route,s(y)}} + m_{\text{CO}_2} \cdot CR_s \cdot C_{p_s^{\text{end}}}(y)\right) \quad (137)$$

where t_s is the transport time of a segment, m_{CO_2} is the daily total CO_2 emissions, CR_s is the capture rate per segment, $C_{\text{t,route,s(y)}}$ is ETS-equivalent carbon tax of the segment at a specific year, $C_{p_s^{\text{end}}}(y)$ is the ETS-equivalent carbon tax of the end port for the segment at a specific year.

The results of the total cost using the route-specific carbon tax are shown in the Supplementary Data 7.

Supplementary Methods 18. Unit penalty

Unit penalty is calculated by dividing the total cost with carbon tax by the total CO_2 emission, as shown in Supplementary Equation (138):

$$UP = \frac{TCT}{M_{CO_2}} \tag{138}$$

The unit penalties for the regeneration scheme possessing under various carbon tax levels and load limits are summarized in Supplementary Data 6 and 7.

Supplementary Methods 19. Flexible offloading strategy for onshore regeneration scheme

In the base-case onshore regeneration scenario, CO_2-rich solvent is assumed to be offloaded at every port for simplicity and consistency across routes. However, in practice, not all ports are expected to be equipped with solvent-handling infrastructure. To evaluate the operational feasibility of the onshore regeneration scheme under realistic port constraints, we introduce a flexible offloading strategy, in which rich-solvent offloading occurs only when required by the load limit.

This section describes the algorithm used to identify the minimum set of must-offload ports for each route and to evaluate the corresponding capture performance and economic outcomes.

Problem formulation

Each route is divided into segments $s = 1, \dots, n$, where each segment connects two consecutive ports. Let Net tonnage denotes the net cargo capacity of the ship (tonne). We define a voyage-wise load limit ω_v, representing the allowable OCCS load as a fraction of the ship's net tonnage (Supplementary Equation (139)):

$$0 \leq \omega_v \leq 30\% \tag{139}$$

The corresponding maximum allowable onboard OCCS load is calculated as Supplementary Equation (140):

$$m_{\max} = \omega_v \cdot \text{Net tonnage} \tag{140}$$

For each segment s, the segment-level CO_2 emission is denoted as $m_{\mathrm{CO_2,prop,s}}$ (Supplementary Equation (3)). The capture system operates at a column capture rate of 90%. λ is the rich-solvent weight ratio. The maximum amount of CO_2 that can be captured on segment s is therefore calculated as Supplementary Equation (141):

$$m_{\mathrm{s,max\ captured}} = m_{\mathrm{CO_2,prop,s}} \times 90\% \tag{141}$$

The corresponding maximum amount of rich solvent generated is calculated as Supplementary Equation (142):

$$m_{\mathrm{s,max\ rich-solvent}} = m_{\mathrm{s,max\ captured}} \times \lambda \tag{142}$$

Load-limit check and offloading logic

At the beginning of the voyage, the cumulative onboard inventory of rich solvent is zero, as shown in Supplementary Equation (143):

$$m_{\mathrm{0,rich-solvent}}^{\mathrm{cum}} = 0 \tag{143}$$

After CO_2 captures along segment s, the tentative cumulative onboard inventory is calculated as Supplementary Equation (144):

$$m_{\text{s,rich-solvent}}^{\text{tent}} = m_{\text{s-1,rich-solvent}}^{\text{cum}} + m_{\text{s,max rich-solvent}} \tag{144}$$

Load limit not exceeded

If the tentative cumulative onboard inventory does not exceed the maximum allowable onboard load, as shown in Supplementary Equation (145), no offloading is required at the terminal port of segment s:

$$m_{\text{s,rich-solvent}}^{\text{tent}} < m_{\text{max}} \tag{145}$$

In this case, the amount of CO_2 captured and the amount of rich solvent generated on this segment are calculated as Supplementary Equation (146) and (147), respectively:

$$m_{\text{s,captured}} = m_{\text{s,max captured}} \tag{146}$$

$$m_{\text{s,rich}} = m_{\text{s,max rich-solvent}} \tag{147}$$

The cumulative onboard inventory is then updated as Supplementary Equation (148):

$$m_{\text{s,rich-solvent}}^{\text{cum}} = m_{\text{s-1,rich-solvent}}^{\text{cum}} + m_{\text{s,rich-solvent}} \tag{148}$$

Load limit exceeded

If the condition in Supplementary Equation (145) is not satisfied, the cumulative OCCS load would exceed the load limit by the end of segment s. In this case, all accumulated rich solvent is required to be offloaded at the port at the end of this segment. Meanwhile, CO_2 capture on the current segment proceeds only until the allowable load m_{max} is reached.

Accordingly, the amount of rich solvent generated on segment s is calculated as Supplementary Equation (149):

$$m_{\text{s,rich}} = m_{\text{s,max}} - m_{\text{s-1,rich-solvent}}^{\text{cum}} \tag{149}$$

The corresponding captured CO_2 is calculated as Supplementary Equation (150):

$$m_{\text{s,captured}} = \frac{m_{\text{s,rich}}}{\lambda} \tag{150}$$

After offloading, the cumulative onboard inventory of rich solvent is reset to zero as shown in Supplementary Equation (151):

$$m_{\text{s,rich-solvent}}^{\text{cum}} = 0 \tag{151}$$

Ports at which offloading occurs are identified as the must-offload ports for the given load ratio ω_v.

Overall capture rate

The overall voyage-level CO_2 capture rate (OCR) is calculated as Supplementary Equation (152):

$$OCR = \frac{\sum_s m_{\mathrm{s,captured}}}{M_{\mathrm{CO_2}}} \tag{152}$$

Optimal load limit

For a given voyage-wise load limit ω_v, the cost metrics—Capture Cost (CC), Total Cost with Carbon Tax (TCT), and Unit Penalty (UP)—are evaluated using the same methodology described in Supplementary Methods 14, 15, 17 and 18.

The optimal voyage-wise load limit for each route is then obtained by minimizing the unit penalty as shown in Supplementary Equation (153):

$$\omega_{v,r}^{*} = arg \min_{0 \leq \omega_v \leq 30\%} UP_r^{2050}(\omega_v) \tag{153}$$

The resulting optimal load limits, must-offload port sets, and associated cost outcomes are reported in Supplementary Data 8.

Supplementary Methods 20. Parameter specifications and nomenclature

Supplementary Table 32. Parameter specifications

Parameter	Value	Unit
Aspen Plus compressor isentropic efficiency	0.8	–
Aspen Plus compressor mechanical efficiency	0.9	–
Aspen Plus compressor model	isentropic using ASME method	
Number of compressor stages	3	–
Onboard conditioning pressure	20.4	bar
Onshore conditioning pressure	120	bar
Onboard conditioning temperature	−28.9	℃
Onshore conditioning temperature	25	℃
USD exchang rate to Chinese YUAN	7.2	CNY/USD
Weight of a FEU container	30.48	tonne

Supplementary Table 33. Nomenclature

**Symbol**	**Description**
A_{col}	Column cross-sectional area (m^2)
a_e	Effective wetted area ($m^2 \cdot m^{-3}$)
A_{df}	Reduced driving-force parameter ($1-\lambda_{cr}$)
a_t	Specific surface area of 250Y packing (250 ($m^2 \cdot m^{-3}$))
B	Corrugation base width (m)
BEC	Total bare-equipment cost (US$)
BFC	Boiler fuel consumption (tonne·GJ^{-1})
C_1	Packing-specific empirical constant
C_{absorber}	Bare-equipment cost of the column shell (US$)
$CACE$	Coefficients for additional carbon emissions
$CAPEX_{\mathrm{MEA,no\ absorber}}$	Capital costs of the main equipment for MEA process (no absorber)
$CAPEX_{\mathrm{NE-2,no\ absorber}}$	Capital costs of the main equipment for NE-2 process (no absorber)
$CAPEX_{\mathrm{onboard}}$	Onboard total capital expenditure (US$)
$CAPEX_{\mathrm{y,onboard}}$	Onboard annualised capital expenditure (US$)
$CAPEX_{\mathrm{y,onshore}}$	Onshore annualised capital expenditure (US$)
CC	Capture cost (US$·$tonne^{-1}$)
C_{cap}	Amount of CO_2 captured in the absorber (kmol·h^{-1})
$C_{\mathrm{cargo\ loss}}$	Cost of cargo loss per voyage (US$)
$C_{\mathrm{cargo\ loss,onboard}}$	Cost of onboard-scheme cargo loss (US$·$tonne^{-1}$ CO_2)
$C_{\mathrm{cargo\ loss,onshore}}$	Cost of onshore-scheme cargo loss (US$·$tonne^{-1}$ CO_2)

CC_{onboard}	Capture cost for onboard regeneration scheme (US\$·tonne^{-1} CO_2)
CC_{onshore}	Capture cost for onshore regeneration scheme (US\$·tonne^{-1} CO_2)
CE	Conditioning energy (GJ·tonne^{-1} CO_2)
C_{equip}	Equipment cost per voyage (US\$)
$C_{\text{equip,onboard}}$	Capital cost of onboard OCCS equipment (US\$·tonne^{-1} CO_2)
$C_{\text{equip,onboard,total}}$	Total onboard bare-equipment cost (US\$)
$C_{\text{equip,onshore}}$	Capital cost of onshore OCCS equipment (US\$·tonne^{-1} CO_2)
$C_{\text{equip,onshore,Absorber}}$	Absorber cost of onshore OCCS equipment (US\$)
$C_{\text{equip,onshore-side,DES}}$	Equipment cost of onshore facilities with DES (US\$·tonne^{-1} CO_2)
$C_{\text{equip,onshore-side,MEA}}$	Equipment cost of onshore facilities with MEA (US\$·tonne^{-1} CO_2)
$C_{\text{equip,onshore-side,NE-2}}$	Equipment cost of onshore facilities with NE-2 (US\$·tonne^{-1} CO_2)
$C_{\text{equip,onshore-side,PZ}}$	Equipment cost of onshore facilities with PZ (US\$·tonne^{-1} CO_2)
$C_{\text{equip,onshore,onboard-side}}$	Onboard absorber cost of onshore OCCS (US\$·tonne^{-1} CO_2)
$C_{\text{equip,onshore,onshore-side}}$	Onshore facilities capital cost of onshore OCCS (US\$·tonne^{-1} CO_2)
$C_{\text{initial solvent,onboard}}$	Onboard start-up solvent cost (US\$·tonne^{-1} CO_2)
$C_{\text{initial solvent,onboard}}$	Onshore start-up solvent cost (US\$·tonne^{-1} CO_2)
$C_{\text{conditioning}}$	Cost of conditioning per voyage (US\$)
$C_{\text{conditioning,onboard}}$	Cost of onboard conditioning per tonne of CO_2 (US\$·tonne^{-1} CO_2)
$C_{\text{conditioning,onshore}}$	Cost of onshore conditioning per tonne of CO_2 (US\$·tonne^{-1} CO_2)
$C_{\text{make-up}}$	Solvent make-up cost (US\$·tonne^{-1} CO_2)
$C_{\text{make-up,DES}}$	Solvent make-up cost of DES (US\$·tonne^{-1} CO_2)
$C_{\text{make-up,MEA}}$	Solvent make-up cost of MEA (US\$·tonne^{-1} CO_2)
$C_{\text{make-up,NE-2}}$	Solvent make-up cost of NE-2 (US\$·tonne^{-1} CO_2)
$C_{\text{make-up,PZ}}$	Solvent make-up cost of PPZ (US\$·tonne^{-1} CO_2)
$C_{P,0}$	Packing -specific constant (0.292)
C_{Packing}	Bare-equipment cost of structured packing (US\$)
$C_{p,\text{DES}}$	Heat capacity of DES (kJ·kg^{-1}·K^{-1})
$C_{p_s^{\text{end}}}(y)$	Carbon tax at end port of a segment (US\$·tonne^{-1} CO_2)
$C_{p_s^{\text{start}}}(y)$	Carbon tax at start port of a segment (US\$·tonne^{-1} CO_2)
$C_p(y)$	CO_2 tax of the port in year y
$C_{\text{regeneration}}$	Cost of regeneration per voyage (US\$)
$C_{\text{regeneration,onboard}}$	Cost of onboard regeneration per tonne of CO_2 (US\$·tonne^{-1} CO_2)
$C_{\text{regeneration,onshore}}$	Cost of onshore regeneration per tonne of CO_2 (US\$·tonne^{-1} CO_2)
CR_{col}	Column capture rate (90%)
CR_s	Capture rate per segment

$C_{r(p)}(y)$	CO_2 tax at the region of the port in year y
C_s	Packing-specific constant loading (3.157)
C_{OCCS}	Cost of the OCCS system per voyage (US$)
$C_{\text{OCCS,onboard}}$	Cost of onboard OCCS system per voyage (US$)
$C_{\text{OCCS,onshore}}$	Cost of onshore OCCS system per voyage (US$)
$C_{\text{s,cargo loss,onboard}}$	Cost of onboard cargo loss per segment (US$)
$C_{\text{s,cargo loss,onshore}}$	Cost of onshore cargo loss per segment (US$)
c_{solu}	Solvent concentration (30% for MEA and PZ)
$C_{\text{solvent,onboard}}$	Total onboard solvent cost (US$·$\text{tonne}^{-1}$ CO_2)
$C_{\text{solvent,onshore}}$	Total onshore solvent cost (US$·$\text{tonne}^{-1}$ CO_2)
C_t	Carbon tax (US$·$\text{tonne}^{-1}$)
$C_{\text{t,route(y)}}$	Voyage-level ETS-equivalent carbon tax (US$·$\text{tonne}^{-1}$)
$C_{\text{t,route,s(y)}}$	Segment-level ETS-equivalent carbon tax (US$·$\text{tonne}^{-1}$)
D	Tank diameter (m)
d	Tank wall thickness (m)
$D_{CO_2,w(T)}$	Diffusivity of CO_2 in water ($m^2 \cdot s^{-1}$)
d_e	Equivalent packing particle diameter (m)
D_G	Diffusivity of CO_2 in the gas phase ($m^2 \cdot s^{-1}$)
D_L	Diffusivity of CO_2 in the liquid phase ($m^2 \cdot s^{-1}$)
D_{req}	Column diameter (m)
EEF	Electricity emission factor (g CO_2 kwh^{-1})
EEF_G	Global electricity emission factor (g CO_2 kwh^{-1})
EF	Emission factor (3.1 tonnes CO_2 tonne^{-1} fuel)
EFC	SFOC of the marine diesel generator (183 g·kwh^{-1})
EP	Electricity price (US$·$\text{GJ}^{-1}$)
EUF	Energy unit transfer factor
f	Installation factor of CAPEX
FC_{add}	Daily additional fuel consumption (tonne·day^{-1})
FCI	Fixed-capital investment (US$)
$\text{FCI}_{\text{onboard}}$	Onboard fixed-capital investment (US$)
FC_{prop}	Daily propulsion fuel consumption (tonne·day^{-1})
$F_{\text{equip,onboard}}$	Onboard equipment cost factor, as a fraction of absorber shell cost
$f_{\text{reb,MEA}}$	Onshore cost share of the MEA reboiler
$f_{\text{reb,PZ}}$	Onshore cost share of the PZ reboiler
Fr_L	Liquid Froude number based on effective liquid velocity

Fr_l	Liquid Froude number based on superficial liquid velocity
Fr_l^*	Liquid Froude number based on line liquid flux
$f_{STM,0}$	Base value of surrogate model for regeneration heat (GJ·tonne^{-1} CO_2)
g	Gravitational acceleration (9.806 m·s^{-2})
H	Tank length (m)
ΔH_{abs}	CO_2 absorption enthalpy of DES (kJ·mol^{-1})
h	Straight-edge height of the tank head (mm)
H_G	Gas-side height of a transfer unit (gas-film HTU) (m)
H_L	Liquid-side height of a transfer unit (liquid-film HTU) (m)
h_L	Liquid hold-up of column (m^3·m^{-3})
h_p	Corrugation height (m)
h_{static}	Static liquid holdup (m^3·m^{-3})
H_{redist}	Height of each liquid redistributor (0.5 m)
$\mathrm{HTU_{OV}}$	Overall gas-phase height of a transfer unit (m)
h_w	Water latent heat (kJ·mol^{-1})
i	Interest rate of annualised $CAPEX_{y,onboard}$ (8%)
ISBL	Inside-battery-limits cost (US$)
$\mathrm{ISBL_{onboard}}$	Onboard inside-battery-limits cost (US$)
IR	Proportions of industrial energy consumption
IR_{CN}	Proportion of China's industrial energy consumption
IR_{EU}	Proportion of Europe's industrial energy consumption
IR_{US}	Proportion of US's industrial energy consumption
L	Height of the liquid holdup (m)
L'	Line liquid flux in the column (kg·m^{-2}·s^{-1})
L_m	Minimum solvent flow (kmol·h^{-1})
L_o	Actual solvent molar circulation rate (kmol·h^{-1})
LP	Low-pressure steam price (US$·GJ^{-1})
K	Packing parameter
k_g	Gas-film mass-transfer coefficient
k_l	Liquid-side mass transfer coefficient
k_{PZ}	Ratio of total direct costs with PZ and MEA
k_{NE-2}	Ratio of total direct costs with NE-2 and MEA
MCR	Engine maximum continuous rating (kW)
M_{CO_2}	Total CO_2 emissions per voyage (tonne)
m_{CO_2}	Daily total CO_2 emissions (tonne·day^{-1})

$m_{CO_2,add}$	Daily additional CO_2 emissions (tonne·day^{-1})
$m_{CO_2,add,MEA}$	Daily additional CO_2 emissions using MEA (tonne·day^{-1})
$m_{CO_2,annual}$	Annual mass of CO_2 captured by the ship (tonne)
$m_{CO_2,prop}$	Daily propulsion-related CO_2 emission (tonne·day^{-1})
$m_{CO_2,prop,s}$	Propulsion-related CO_2 emission per segment (tonne)
$m_{CO_2,prop,v}$	Propulsion-related CO_2 emission per voyage (tonne)
$m_{0,rich-solvent}^{cum}$	Cumulative onboard inventory of rich solvent at the start port (tonne)
$m_{s,rich-solvent}^{cum}$	Cumulative onboard inventory of rich solvent of at this port (tonne)
$m_{s-1,rich-solvent}^{cum}$	Cumulative onboard inventory of rich solvent at the last port (tonne)
m_{eq}	Slope of the equilibrium y–x line at the lean end
$\dot{m}_G$	Total gas mass flow rate through the column (kg·h^{-1})
$\dot{m}_L$	Total liquid mass flow rate through the column (kg·h^{-1})
m_{max}	Maximum allowable onboard OCCS load (tonne)
m_s	OCCS load per segment (tonne)
$m_{s,captured}$	Captured CO_2 per segment (tonne)
$m_{s,onboard}$	OCCS load of onboard regeneration per segment (tonne)
$m_{s,onshore}$	OCCS load of onshore regeneration per segment (tonne)
$m_{s,max}$	OCCS load of the segment with the maximum transportation days (tonne)
$m_{s,max\ captured}$	Maximum amount of CO_2 captured on segment (tonne)
$m_{s,max\ rich-solvent}$	Maximum amount of rich solvent generated on segment (tonne)
$m_{s,rich}$	Amount of rich solvent generated on this segment (tonne)
m_{tank}	Weight of the storage tanks (tonne)
$m_{s,rich-solvent}^{tent}$	Tentative cumulative onboard inventory on segment (tonne)
MW_{air}	Molecular weight of air (28.5 g·mol^{-1})
MW_{CO_2}	Molecular weight of CO_2 (44 g·mol^{-1})
MW_{liq}	Effective molecular weight of the solvent (g·mol^{-1})
$n_{CO_2,in}$	Inlet CO_2 flow in flue gas (kmol·h^{-1})
$n_{CO_2,out}$	Outlet CO_2 flow in flue gas (kmol·h^{-1})
$n_{gas,in}$	Total inlet flue-gas flow (kmol·h^{-1})
$n_{gas,out}$	Total outlet flue-gas flow (kmol·h^{-1})
$\dot{n}_{L,in}$	Inlet liquid flow (kmol·h^{-1})
$n_{non\ CO_2}$	Carrier gas flow rate (air and other flue-gas species) (kmol·h^{-1})
n_{redist}	Number of required redistributors
n_{seg}	Number of required segments
NTU_{OG}	Overall number of transfer units

OCR	Overall capture rate for the voyage
OEF	Onshore-regeneration emission factor (tonne emitted tonne^{-1} captured)
P	Effective perimete of Bravo model (m^{-1})
p	Port
ΔP	Total pressure drop of column (Pa)
$P_{\mathrm{CO_2}}$	CO_2 partial pressures at the stripper top (kPa)
P_{FEED}	Feed pressure (bar) for determining regeneration heat of MEA and PZ
p_{solv}	Solvent purchase price (US$·tonne^{-1})
$p_{\mathrm{solv,MEA}}$	Solvent purchase price of MEA (US$·tonne^{-1})
P_{w}	Water vapor pressure (kPa)
Q_{lat}	Latent heat of the DES (GJ·tonne^{-1} CO_2)
Q_{reac}	Reaction heat of the DES (GJ·tonne^{-1} CO_2)
Q_{reg}	Regeneration energy of the DES (GJ·tonne^{-1} CO_2)
Q_{sens}	Sensible heat of the DES (GJ·tonne^{-1} CO_2)
RE	Regeneration heat (GJ·tonne^{-1} CO_2)
Re_g	Reynolds number of the gas stream
$Re_g{}^*$	Reynolds number based on effective gas velocity
Re_l	Reynolds number of the liquid stream
$Re_l{}^*$	Reynold number based on line liquid flux
r_{CAP}	Capture rate parameter for determining regeneration heat of MEA and PZ
$r(p)$	Region of the ports
s	Segment
S	Packing wave length (m)
Sc_g	Gas-phase Schmidt number based on the effective gas velocity
SEF	Steam emission factor (g CO_2 kwh^{-1})
SEF_{CN}	China's steam emission factor (g CO_2 kwh^{-1})
SEF_{EU}	Europe's steam emission factor (g CO_2 kwh^{-1})
SEF_G	Global steam emission factor (g CO_2 kwh^{-1})
SEF_{US}	US's steam emission factor (g CO_2 kwh^{-1})
$SFOC$	Specific fuel oil consumption (g·kwh^{-1})
TCT	Total cost with carbon tax (US$)
TCT_{onboard}	Total cost for the onboard regeneration scheme (US$)
TCT_{onshore}	Total cost for the onshore regeneration scheme (US$)
$TDC_{\mathrm{MEA,no\ absorber}}$	Total direct equipment cost of MEA process without absorber (US$)
$TDC_{\mathrm{PZ,no\ absorber}}$	Total direct equipment cost of PZ process without absorber (US$)

t	Lifetime of a ship (25 years)
$t(m)$	Wall thickness of the column shell (m)
t_s	Transportation days per segment (day)
$t_{\text{s,max}}$	Maximum transportation days per voyage (day)
U_{ge}	Effective gas velocities inside the corrugation channels ($m \cdot s^{-1}$)
U_{gs}	Superficial gas velocity ($m \cdot s^{-1}$)
UP	Unit penalty (US$·$tonne^{-1}$ CO_2)
UP_r^{2050}	Unit penalty at 2050 for each route (US$·$tonne^{-1}$ CO_2)
U_{le}	Effective liquid velocities inside the corrugation channels ($m \cdot s^{-1}$)
U_{ls}	Superficial liquid velocity ($m \cdot s^{-1}$)
$V_{CO_2,\text{max}}$	Maximum volume of the CO_2 captured (m^3)
V_{Packing}	Packing volume (m^3)
$V_{\text{s,captured}}$	Volume of the CO_2 captured per segment (m^3)
V_{tank}	Volume of the CO_2 storage tank (m^3)
We_l	Weber numbers based on line liquid flux
W_{vessel}	Bare steel weight of the column shell (kg)
x_{CO_2}	CO_2 molar fraction
X_0	Lean solvent loading at the absorber bottom ($kmol \cdot kmol^{-1}$)
$X_{u,m}$	Required rich loading ($kmol \cdot kmol^{-1}$)
Y_o	Outlet CO_2 ratios (kmol CO_2 $kmol^{-1}$ air)
Y_u	Inlet CO_2 ratios (kmol CO_2 $kmol^{-1}$ air)
Z	Theoretical packed height (HETP) (m)
Z_{design}	Design packed height (m)
Z_{seg}	Segments of maximum length of packing (6 m)
Z_{total}	Structural packed height (m)

**Greek letter**	**Description**
α	Sensitivity coefficient for determining regeneration heat of MEA and PZ
β	Weighting factor for determining regeneration heat of MEA and PZ
Γ	Liquid line load of packing ($kg \cdot m^{-1} \cdot s^{-1}$)
γ	Volumetric CO_2 absorption capacity ($mol \cdot L^{-1}$)
ε	Void fraction of 250Y packing (0.97)
η	Engine load factor
λ	Rich-solvent weight ratio (tonne rich-solvent $tonne^{-1}$ CO_2)
λ_{cr}	Capacity ratio under the slope of the equilibrium line
μ_G	Flue-gas viscosity ($Pa \cdot s$)

μ_L	Viscosity of the material in liquid phase (Pa·s)
$\mu_{\mathrm{mix(T)}}$	Viscosity of the solvent (MEA, PZ, NE-2, and DES) (Pa·s)
$\mu_{\mathrm{w(T)}}$	Viscosity of pure water (Pa·s)
o	A commonly used exponent for aqueous amine systems (0.6)
$\rho_{\mathrm{CO_2}}$	Density of liquefied CO_2 (tonne·m^{-3})
ρ_G	Flue-gas density (kg·m^{-3})
ρ_L	Density of the material in liquid phase(kg·m^{-3})
ρ_{steel}	Steel density (kg·m^{-3})
σ	Surface tension of solvent (N·m^{-1})
σ_c	Critical surface tension of packing material (0.072 N·m^{-1})
ϕ	Solvent-to-minimum ratio (40% above the minimum solvent flow)
ψ_{dry}	Resistance coefficient on the gas side
ψ_L	Resistance coefficient of two-phase flow
ω_{limit}	Load limit
ω_{max}	Maximum OCCS-load-To-ship-net-tonnage ratio across the voyage
ω_s	OCCS-load-To-ship-net-tonnage ratio for a segment
ω_v	Voyage-wise load ratio
$\omega_{v,r}^*$	Optimal voyage-wise load ratio for each route

Supplementary References

1. DNV. MAERSK ENSHI DNV Vessel Register. https://vesselregister.dnv.com/vesselregister/details/37365 (accessed 14 June 2024).
2. DNV. DNV Vessel Register. https://vesselregister.dnv.com/vesselregister (accessed 14 June 2024).
3. DNV. COSCO KOREA DNV Vessel Register. https://vesselregister.dnv.com/vesselregister/details/38731 (accessed 14 June 2024).
4. DNV. NORTHERN JAGUAR DNV Vessel Register. https://vesselregister.dnv.com/vesselregister/details/G114330 (accessed 14 June 2024).
5. DNV. MSC FEBE DNV Vessel Register. https://vesselregister.dnv.com/vesselregister/details/39598 (accessed 14 June 2024).
6. DNV. MAERSK CINCINNATI DNV Vessel Register. https://vesselregister.dnv.com/vesselregister/details/43631 (accessed 14 June 2024).
7. ABS. MAERSK HAMBURG ABS RECORD. https://absrecord.eagle.org/#/absrecord/details (accessed 14 June 2024).
8. ABS. ABS RECORD, the online database for ABS vessel records. https://absrecord.eagle.org/#/absrecord/assets (accessed 14 June 2024).
9. ABS. MAERSK TAIKUNG ABS RECORD. https://absrecord.eagle.org/#/absrecord/details (accessed 14 June 2024).
10. DNV. MSC PALOMA DNV Vessel Register. https://vesselregister.dnv.com/vesselregister/details/G113679 (accessed 14 June 2024).
11. ClassNK. ClassNK Register of Ships - M/S MAERSK QINZHOU(CNo.202421). https://www.classnk.or.jp/register/regships/one.aspx (accessed 14 June 2024).
12. ClassNK. Register of Ships | ClassNK. https://www.classnk.or.jp/register/regships/regships.aspx (accessed 14 June 2024).
13. BV. GSL VINIA BV Fleet | Marine & Offshore https://marine-offshore.bureauveritas.com/bv-fleet/#/bv-fleet (accessed 14 June 2024).
14. BV. BV Fleet | Marine & Offshore. https://marine-offshore.bureauveritas.com/bv-fleet/#/bv-fleet (accessed 14 June 2024).
15. CCS. XIN HUANG PU Register of Ships. https://www.ccs.org.cn/ccswzen/internationalShipsList?columnid=201900002000000123&imono=9310044 (accessed 13 June 2024).
16. CCS. CCS Register of Ships. https://www.ccs.org.cn/ccswzen/internationalShipsList?columnid=201900002000000

123 (accessed 14 June 2024).

17. CCS. REN JIAN 26 Register of Ships. https://www.ccs.org.cn/ccswzen/internationalShipsList?columnid=201900002000000123&imono=9450947 (accessed 13 June 2024).

18. ClassNK. ClassNK Register of Ships - M/S MAERSK NORESUND(CNo.212270) https://www.classnk.or.jp/register/regships/one.aspx (accessed 14 June 2024).

19. ClassNK. MAERSK NINGBO ABS RECORD. https://absrecord.eagle.org/#/absrecord/details (accessed 14 June 2024).

20. MAN Diesel & Turbo. Improved SFOC with G95ME-C9.6 Engines (MAN Diesel & Turbo, 2017).

21. Luo, X. & Wang, M. Study of solvent-based carbon capture for cargo ships through process modelling and simulation. *Appl. Energy* **195**, 402-413 (2017).

22. MAN Energy Solutions. Four-stroke diesel engine compliant with IMO Tier II (MAN Energy Solutions, 2017).

23. Mitsubishi Power Systems Headquarters. UE diesel engines manuals (Tokyo, 2017).

24. MARAD. Marine Carbon Capture Technology Review (MARAD, 2022).

25. McCoy, S. T. & Rubin, E. S. An engineering-economic model of pipeline transport of CO2 with application to carbon capture and storage. *Int. J. Greenh. Gas Control* **2**, 219-229 (2008).

26. Chung, W. & Lee, J. H. Input–Output Surrogate Models for Efficient Economic Evaluation of Amine Scrubbing CO_2 Capture Processes. *Ind. Eng. Chem. Res.* **59**, 18951–18964 (2020).

27. Liu, F., Qi, Z., Fang, M. & Ding, H. Pilot test of water-lean solvent of 2-(ethylamino) ethanol, 1-methyl-2-pyrrolidinone, and water for post-combustion CO_2 capture. *Chem. Eng. J.* **459**, 141634 (2023).

28. Liu, F., Qi, Z., Fang, M. & Ding, H. Pilot Test and Techno-economic Assessment on Water-lean Amine-based CO_2 Capture Process. *Proc. Chin. Soc. Electr. Eng.* **44**, 3897-3904 (2024).

29. Darwish, A. S., Alwan, R. A., Boublia, A., Lemaoui, T., Benguerba, Y., AlNashef, I. M. et al. Machine learning approach for mapping the heat capacity of deep eutectic solvents for sustainable energy applications. *Fuel* **381**, 133278 (2025).

30. Trivedi, T. J., Lee, J. H., Lee, H. J., Jeong, Y. K. & Choi, J. W. Deep eutectic solvents as attractive media for CO_2 capture. *Green Chem.* **18**, 2834-2842 (2016).

31. Ship & Bunker. Global 20 Ports Average Bunker Prices https://shipandbunker.com/prices/av/global/av-g20-global-20-ports-average (accessed 8 February 2024).

32. El Hadri, N., Quang, D. V., Rayer, A. V. & Abu-Zahra, M. R. M. *Development of Amine-blend Systems for CO_2 Post-Combustion Capture*. In Proceedings of the 4th International Gas Processing Symposium, 59-68 (Elsevier Press, Oxford, 2015).

33. Foorginezhad, S. & Ji, X. Development of monoethanolamine chloride-ethylene diamine deep eutectic solvent for ffficient carbon dioxide capture. *Sep. Purif. Technol.* **347**, 127593 (2024).

34. Amundsen, T. G., Øi, L. E. & Eimer, D. A. Density and viscosity of monoethanolamine+ water+ carbon dioxide from (25 to 80) C. *J. Chem. Eng. Data* **54**, 3096-3100 (2009).

35. Freeman, S. A. & Rochelle, G. T. Density and viscosity of aqueous (piperazine+ carbon dioxide) solutions. *J. Chem. Eng. Data* **56**, 574-581 (2011).

36. Liu, F., Liu, S., Li, N., Tang, W., Sun, S., Fan, H. et al. Mass transfer and kinetic measurement and modeling for CO_2 absorption into aqueous EAE and water-lean EAE/NMP solvents. *Chem. Eng. J.* **492**, 152346 (2024).

37. Versteeg, G. F. & Van Swaaij, W. P. Solubility and diffusivity of acid gases (carbon dioxide, nitrous oxide) in aqueous alkanolamine solutions. *J. Chem. Eng. Data* **33**, 29-34 (1988).

38. Munson, B. R., Okiishi, T. H., Huebsch, W. W. & Rothmayer, A. P. *Fundamentals of Fluid Mechanics, 7th edn* (Wiley, New York, 2013).

39. Tariq, M., Freire, M. G., Saramago, B., Coutinho, J. A., Lopes, J. N. C. & Rebelo, L. P. N. Surface tension of ionic liquids and ionic liquid solutions. *Chem. Soc. Rev.* **41**, 829-868 (2012).

40. Rocha, J. A., Bravo, J. L. & Fair, J. R. Distillation columns containing structured packings: a comprehensive model for their performance. 1. Hydraulic models. *Ind. Eng. Chem. Res.* **32**, 641-651 (1993).

41. Treybal, R. E. *Mass transfer operations* (McGraw-Hill, New York, 1980).

42. Tian, Z., Zhou, J., Zhang, Y. & Gao, W. Targeting zero energy increment for an onboard CO_2 capture system: 4E analyses and multi-objective optimization. *Energy Convers. Manage.* **301**, 117890 (2024).

43. Billet, R. & Schultes, M. Prediction of mass transfer columns with dumped and arranged packings: updated summary of the calculation method of Billet and Schultes. *Chem. Eng. Res. Des.* **77**, 498-504 (1999).

44. Rocha, J. A., Bravo, J. L. & Fair, J. R. Distillation columns containing structured packings: a comprehensive model for their performance. 2. Mass-transfer model. *Ind. Eng. Chem. Res.* **35**, 1660-1667 (1996).

45. Onda, K., Sada, E. & Takeuchi, H. Gas absorption with chemical reaction in packed columns. *J. Chem. Eng. Jpn.* **1**, 62-66 (1968).

46. Whaley, A. K., Bode, C. A., Ghosh, J. & Eldridge, R. B. HETP and pressure drop prediction for structured packing distillation columns using a neural network model. *Ind. Eng. Chem. Res.* **38**, 1736-1739 (1999).

47. Seader, J. D., Henley, E. J. & Roper, D. K. *Separation process principles* (Wiley, New York, 1998).

48. Kister, H. Z. *Distillation design* (McGraw-hill, New York, 1992).

49. Visonà, M., Bezzo, F. & d'Amore, F. Techno-economic analysis of onboard CO_2 capture for ultra-large container ships. *Chem. Eng. J.* **485**, 149982 (2024).

50. Ding, X., Chen, H., Li, J. & Zhou, T. Comparative techno-economic analysis of CO_2 capture processes using blended amines. *Carbon Capture Sci. Technol.* **9**, 100136 (2023).

51. Otitoju, O., Oko, E. & Wang, M. Technical and economic performance assessment of post-combustion carbon capture using piperazine for large scale natural gas combined cycle power plants through process simulation. *Appl. Energy* **292**, 116893 (2021).

52. Toocle. Latest price of monoethanolamine - Quotation Center - Toocle https://www.100ppi.com/mprice/plist-1-1346-1.html (accessed 14 June 2024).

53. Toocle. Latest price of piperazine anhydrous - Quotation Center - Toocle. https://www.100ppi.com/mprice/plist-1-4422-1.html (accessed 14 June 2024).

54. Yalamov, D., Georgiev, P. & Garbatov, Y. Economic Feasibility of Retrofitting an Ageing Ship to Improve the Environmental Footprint. *Appl. Sci.* **13**, 1199 (2023).

55. Morken, A. K., Pedersen, S., Kleppe, E. R., Wisthaler, A., Vernstad, K., Ullestad, Ø. et al. Degradation and emission results of amine plant operations from MEA testing at the CO_2 technology centre mongstad. *Energy Procedia* **114**, 1245-1262 (2017).

56. IEA. World Energy Outlook 2024 (IEA, 2024).

57. IEA. Net Zero by 2050 (Paris, 2021).

58. Hu, Y., Gani, R., Sundmacher, K. & Zhou, T. Assessing the future impact of 12 direct air capture technologies. *Chem. Eng. Sci.* **298**, 120423 (2024).

59. IEA. North America – Countries & Regions – Energy consumption by sector. https://www.iea.org/regions/north-america/energy-mix (accessed 25 Oct 2024).

60. IEA. Europe – Countries & Regions – Energy consumption by sector. https://www.iea.org/regions/europe/energy-mix (accessed 25 Oct 2024).

61. IEA. China - Countries & Regions - Energy consumption by sector. https://www.iea.org/countries/china/energy-mix (accessed 25 Oct 2024).
