# Supplementary material for: Rethinking solvent regeneration pathways for maritime carbon capture
Source: Nat Commun. 2026 Jun 30;17:8157. doi: 10.1038/s41467-026-74909-w (PMC13458838; doi:10.1038/s41467-026-74909-w)
Supplement: Supplementary file 2 [file 41467_2026_74909_MOESM2_ESM.pdf]

Supplementary Data 1-8 .xlsx (Excel workbook with 8 sheets)

Sheet 1: "Supplementary Data 1: Onboard regeneration (for 16 representative routes)"

Purpose: Cost and performance results for onboard regeneration across 16 representative routes.

Structure: Each row corresponds to a route–scenario combination. Columns contain inputs/outputs used in the onboard cost model.

Key fields:

- Route ID: Identifier for representative route
- Transportation days per segment (days)
- Propulsion CO_2 emissions per segment (tonne)
- Daily propulsion-related CO_2 emissions (tonne/day)
- Load limit: CO_2 load limit as % of net tonnage (e.g., 10/20/30)
- Capture results for onboard regeneration across 16 representative routes under different load limits (e.g., 10%/20%/30%)
- Daily additional CO_2 emissions (tonne/day)
- Daily total CO_2 emissions (tonne/day)
- Additional CO_2 emissions per segment (tonne)
- Total CO_2 emissions per segment (tonne)
- Daily CO_2 captured (tonne/day)
- OCCS load per segment (tonne)
- Capture rate per segment
- Capture results for onboard regeneration under "capture rate at 90%"
- Additional CO_2 emissions per segment (tonne)
- OCCS load per segment (tonne)
- OCCS-load-To-ship-net-tonnage ratio per segment
- Storage tank weight under different weight load limit for onboard regeneration scheme
- Maximum weight of CO_2 in tanks (tonne)
- Volume of ship's storage tank (m^3)
- Tank length (m)
- Tank diameter (m)
- Storage tank weight (tonne)
- Storage tank information at 90% capture rate
- Maximum CO_2 captured/voyage (tonne)

- Volume of tank (V_tank) (m^3)

- Ship's width(m)

- Tank's length(m)

- Tank diameter (m)

- Weight of the storage tank(tonne)

Sheet 2: "Supplementary Data 2: Onshore regeneration (for 16 representative routes)"

Purpose: Cost and performance results for onshore regeneration across 16 representative routes.

Structure: Each row corresponds to a route–scenario combination. Columns contain inputs/outputs used in the onshore cost model.

Key fields:

- Route ID: Identifier for representative route

- Transportation days per segment (days)

- Propulsion CO_2 emissions per segment (tonne)

- Daily propulsion-related CO_2 emissions (tonne/day)

- Load limit: CO_2 load limit as % of net tonnage (e.g., 10/20/30)

- Capture results for onshore regeneration across 16 representative routes under different load limit (e.g., 10%/20%/30%)

- CO_2 captured per segment (tonne)

- Daily total CO_2 emissions (tonne/day)

- OCCS load per segment (tonne)

- Capture rate per segment

- Capture results for onshore regeneration under "capture rate at 90%"

- OCCS load per segment (tonne)

- OCCS-load-To-ship-net-tonnage ratio per segment

Sheet 3: "Supplementary Data 3: Unit penalty (UP) across 357 Maersk routes using the DES-based onshore regeneration scheme (US$/tonne CO_2)"

Purpose: Route-level unit penalty results for the full set of 357 routes under DES-based onshore regeneration.

Structure: Each row corresponds to a route. Columns provide UP values under multiple scenarios/years/load limits.

Key fields:

- Route ID: Identifier for representative route

- Load limit: CO_2 load limit as % of net tonnage (e.g., 10/20/30)

- Unit penalty (UP): Cost per tonne of emitted CO_2 [US$/tonne CO_2]

Sheet 4: "Supplementary Data 4: Equipment cost"

Purpose: Equipment cost breakdown used in the cost model.

Key fields:

- Equipment item: Absorber
- Z_design (m)
- D_req (m)
- Packing(m^3)
- Packing cost(US$)
- Wvessel(kg)
- Total equipment cost(US$)
- CAPEX: Capital expenditure [US$]
- Equipment cost: Equipment cost per tonne of captured CO_2 [US$/tonne CO_2]

Sheet 5: "Supplementary Data 5: Solvent cost"

Purpose: Solvent inventory and make-up costs used in the model.

Key fields:

- Initial inventory: Initial solvent cost for different regeneration scheme [US$/tonne CO_2]

Notes: [This table provides a breakdown of the initial solvent cost; the make-up solvent cost is reported in Supplementary Note 12.]

Sheet 6: "Supplementary Data 6: Total cost and unit penalty"

Purpose: Consolidated outputs (total cost and UP) for solvents and scenarios.

Key fields:

- Capture CO_2 (tonne)
- Capture cost & components (US$/tonne CO_2)
- Cost of cargo (US$/tonne)
- Cost of regeneration and liquefaction (US$/tonne)
- Cost of equipment (US$/tonne)
- Cost of solvent (US$/tonne)
- Capture cost (US$/tonne)
- Total cost & components (US$) and Unit Penalty (US$/tonne)
- under different solvent (MEA/PZ/NE-2/DES) & load limit (e.g., 10%/20%/30%) & year (2030/2040/2050)
- Cargo Cost (US$)
- Regeneration and Liquefaction (US$)
- Equipment Cost (US$)
- Solvent Cost (US$)

- Tax-to-pay (US$)

- Total cost with Carbon Tax (US$)

- Unit Penalty (US$/tonne)

Sheet 7: "Supplementary Data 7: ETS-equivalent carbon tax scenarios"

Purpose: Scenario definitions for ETS-equivalent carbon tax settings used in the analysis.

Key fields:

- ETS-equivalent carbon tax for every segment

- location class code: Ports are classified into four location categories (1–4), representing EU ports, ports in developed regions, ports in developing regions, and ports in other regions, respectively.

- segment code: An index (1–16) for segments derived from all pairwise combinations of the four port categories, used to reference the carbon tax applied between each port pair.

- Carbon tax by region: Summary of ETS-equivalent carbon tax for every route under different years (US$/tonne)

- Total cost & Unit Penalty under ETS-equivalent carbon tax scenarios

- under different load limit (e.g., 10%/20%/30%)

- under different solvent (MEA/PZ/NE-2/DES)

- under different year (2030/2040/2050)

Sheet 8: "Supplementary Data 8: Flexible offloading strategy"

Purpose: Inputs/outputs related to the flexible offloading strategy under DES onshore regeneration scheme.

Key fields:

- Must-offload ports results and Cargo cost (US$) for flexible offloading strategy under DES onshore regeneration scheme

- Cost metrics for flexible offloading strategy

- Transportation price (US$/tonne)

- Optimal load limit

- Capture Rate

- Cargo cost (US$/tonne)

- Equip cost (US$/tonne)

- Solvent cost (US$/tonne)

- Regeneration Conditioning cost (US$/tonne)

- Capture cost (US$/tonne)

- Total cost (US$) & Unit Penalty (US$/tonne) under different years
