# Supplementary material for: Rethinking solvent regeneration pathways for maritime carbon capture
Source: Nat Commun. 2026 Jun 30;17:8157. doi: 10.1038/s41467-026-74909-w (PMC13458838; doi:10.1038/s41467-026-74909-w)
Supplement: Supplementary file 3 [file 41467_2026_74909_MOESM4_ESM.pdf]

nature portfolio

Peer Review File

Rethinking Solvent Regeneration Pathways for Maritime Carbon Capture

Corresponding Author: Professor Teng Zhou

Parts of this Peer Review File have been redacted as indicated to remove third-party material.

This file contains all reviewer reports in order by version, followed by all author rebuttals in order by version.

Version 0:

Reviewer comments:

Reviewer #1

(Remarks to the Author)
The concept of onshore regeneration was proposed in this paper, and the comparison between the onboard/onshore regenerations was conducted. The paper is interesting while major revisions should be made before publication.
1. In Abstract section, some description is unclear, e.g., " The onshore scheme is more cost-effective in most scenarios, … SBCC becomes economically viable in 2040 and highly advantageous by 2050, reinforcing the significant long-term potential of the proposed onshore regeneration approach.". Please detail it.
2. In Introduction, seldom previous onboard carbon capture work including simulation and experiment research was motioned, most citations (i.e., references 24-39) are just the newspaper report. It is necessary to review the progress technology, energy consumption and discuss the problem of onboard carbon capture.
3.The simulation method, software, model, parameters for CO2 capture, regeneration and liquefaction should be detailed in the manuscript, and the accuracy verification of the simulation should be provided.
4. In the reviewer's opinion, the regeneration heats for PZ, NE-2 AND DES (in Table 2) are not accurate. Their regeneration heats are ~50% lower that of MEA, it is difficult to reach so low value unless coupling with energy-saving processes. Please check it.
5. As for the total SBCC cost $CSBCC$ (USD) for both onboard and onshore regeneration schemes, please clarify the parameter of cargo loss, what's the difference between onboard and onshore regeneration scheme?
6.The utilization waste heating and cold energy is important for the onboard regeneration as described in the references 40-42, it is necessary to discuss this topic in the manuscript.
7.The conclusions should be more specific and sufficient to provide the key discovery.

Reviewer #2

(Remarks to the Author)
Overall comment: While the study discusses a very important topic in on-board carbon capture (on-board versus on-shore solvent regeneration evaluating several solvent systems), the study's methodology fails to consider several important aspects to properly design and evaluate an on-board/on-shore capture system for the different solvents considered. For example, the solvent selection argumentation is thin, and a tiny selection of performance parameters is used, while important aspects like solvent kinetics, which partly determines absorber dimensions, are completely neglected. Instead, for the CAPEX calculation, a fixed factor is applied to the total system cost correlation to determine absorber CAPEX, and this approach seems to be the same for each solvent. Additionally, there is little discussion on the selection of the regeneration energy demands of the different solvents considered in this study, while this has a high impact on the results. These are too big simplification that affect the calculations and conclusions greatly, and limits the usefulness of the study. This is reflected in the selection of the DES solvent (only based on high capacity and relatively low reboiler duty) as the optimal solvent in the final study, while the reference paper used for DES does not even seem to discuss kinetics. It is noted that a limitation on equipment costing is added to the conclusions, but this seems insufficient to account for the inaccuracy that these simplifications are giving. More detailed simulations and cost calculations for all solvent systems separately is at least required to increase the usefulness of the study.

Specific comments:
63 – This statement requires quantification, explanation and referencing

64 – Is there a reason only a steel plant is mentioned here, these CO2 concentrations are very high, already in the range where other technologies than chemical absorption become favorable. I think it would be more fair to compare the CO2 concentration towards other post-combustion applications in the 5-20 vol% range (e.g. gas-fired power systems, waste-to-energy, coal-fired)

68 – doesn't have to increase always. If enough heat can be recovered from the flue gases, the fuel penalty might be limited. Is any heat recovery analysis included in the study?

74 – Is liquefaction always required? What if the shore systems are connected to pipeline transportation? Suggesting to change liquefaction to conditioning

117 – This seems to be calculated assuming 30 wt% MEA. Should be mentioned.

178 – Absorption capacity is not the only metric that determines whether a solvent is a good candidate. Kinetics for example are very important for determination of the absorber dimensions, something this study does not cover.

245 – It is understood that using all simplifications, DES comes out as the best solvent (high capacity, good regeneration energy), but this fails to take into account a lot of aspects required for designing a capture system with this solvent. An example: what absorber height would be required to reach the CO2 capacity in the solvent considered in this study?

340 – This is only true is HFO is assumed as the fuel. Is this the case?

362 – The choice to evaluate MEA and PZ seems obvious, given the broad information basis for both solvents The selection criteria seems to be quite thin for NE-2 and DES. The NE-2 regeneration duty seems to be based on a single small pilot study. Regarding DES, at equilibrium these high loading conditions might be achieved, but is it realistic to assume this is possible on a vessel (and what absorber height would be required)? A more detailed discussion on solvent selection is required to justify selection of these solvents.

428 – In the supplementary information, it is explained that the total CAPEX is determined using a simple correlation only using the MCR and capture rate. This is a big simplification, especially if the differences between the solvents are not taken into account. It is also assumed that the absorber CAPEX is always a fixed percentage of the total costs, which is again a big simplification, especially considering the inherent different (e.g. in kinetics) between the different solvents.

445 – The cost metrics are based on the mass flow of CO2 capture captured. This does not seem to take additional emissions into account that are caused by the capture system. CO2 avoidance, taking these additional emissions into account, should be calculated for a 'real' emission reduction measure. It can be expected that regulatory frameworks will also work with CO2 avoidance instead of CO2 captured.

450 – From this text, it is not clear how CAPEX is annualized and how this is taken into account in the calculations

Reviewer #3

(Remarks to the Author)
This manuscript presents a comparative techno-economic analysis of onboard versus onshore solvent regeneration schemes for ship-based carbon capture (SBCC). The concept of onshore regeneration is original and of high relevance to maritime decarbonization, and the comparative framework across multiple routes, solvents, and projected carbon tax scenarios provides practical value. The study therefore has the potential to make a meaningful contribution to both the academic literature and policy discussions on SBCC deployment.
Nevertheless, there are several issues related to clarity, methodological robustness, and contextualization that need to be addressed to strengthen the manuscript. My detailed comments are as follows:
1、 Terminology of load limits – The manuscript uses the term "SBCC load limits" to describe capture capacity in both onboard and onshore regeneration schemes. However, the physical meaning of the load differs substantially between the two cases: onboard regeneration is constrained primarily by the weight of liquefied CO_2 and storage tanks, whereas onshore regeneration is limited by the much larger solvent inventory required to store CO_2-rich solutions. Using the same terminology for both schemes may create ambiguity for readers and obscure these fundamental differences. I suggest the authors adopt distinct or more precise terminology to differentiate the constraints for onboard and onshore regeneration—for example, specifying "CO_2 storage load limit" for onboard systems and "solvent storage load limit" for onshore systems. This would improve clarity and avoid potential misinterpretation.
2、 Load ratio interpretation – The manuscript states that "all schemes achieve relatively low load ratios, averaging 0.25". However, Figure 2b shows that for the onshore regeneration scheme, a significant number of routes exceed this average value, with some load ratios reaching as high as 0.82. This discrepancy suggests that the "average" description may obscure important differences across routes. I recommend that the authors analyze the specific cases with notably higher load ratios, discussing the operational implications and whether such routes remain feasible for onshore regeneration. This additional analysis would provide a more balanced and transparent assessment of the scheme's applicability.
3、 Low capture rate routes –The results show that the capture rate of the onshore regeneration scheme ranges widely from

20% to 82%. However, the manuscript does not discuss which specific routes fall into the lower end of this range, nor whether such low capture rates undermine the practical applicability of the scheme for those cases. To improve the relevance of the findings, I suggest the authors explicitly identify the routes where capture rates are too low to justify onshore regeneration and discuss the potential reasons (e.g., voyage duration, solvent load, ship size). This would help readers better understand the operational boundaries of the proposed technology and avoid overgeneralizing its applicability across all routes.
4、 Carbon pricing mechanisms –The manuscript provides a detailed analysis of how future carbon tax levels influence the economic viability of SBCC schemes. However, in practice, carbon pricing in the maritime sector is not limited to taxation—emissions trading systems (ETS), such as the EU ETS, are already being extended to international shipping and are expected to play an increasingly significant role. Since ETS mechanisms differ from carbon taxes in design and market dynamics (e.g., allowance trading, price volatility, regional disparities), their impacts on SBCC adoption could be materially different. I recommend that the authors at least discuss the potential influence of carbon trading schemes on SBCC economics, or provide a sensitivity analysis incorporating carbon allowance prices. This addition would broaden the policy relevance of the study and make its conclusions more robust under real-world regulatory frameworks.
5、 Solvent management strategies – In the economic evaluation, the onshore regeneration scheme is shown to require substantially larger volumes of solvent than onboard regeneration. However, the manuscript does not clarify how solvent management is envisioned in practice—whether ships would replace the CO_2-rich solvent with new solvent at each port call, or whether the same solvent would be regenerated onshore and then reloaded. These two approaches have very different implications for cost, logistics, and sustainability. Moreover, solvent cost assumptions are not explicitly discussed, even though solvent prices and availability could significantly influence the economic viability of the onshore scheme. I recommend that the authors elaborate on solvent replenishment strategies and provide at least a qualitative discussion of how solvent costs might affect the overall economic assessment.
6、 Uncertainties in solvent performance –The analysis heavily relies on performance assumptions for emerging solvents (NE-2, DES). However, the uncertainties in solvent stability, degradation, and long-term operability are only briefly acknowledged. A sensitivity analysis on solvent performance (e.g., variation in regeneration heat, degradation rates) would greatly strengthen the robustness of the conclusions.
7、 Port infrastructure and logistics –The assumption that CO_2-rich solvents can be offloaded at every port is highly idealized. In practice, solvent transfer logistics, storage infrastructure, and safety regulations may vary widely. The authors should provide a discussion of infrastructure requirements and realistic port compatibility, potentially including a scenario where offloading is only available at major hub ports.
8、 Contextualization of novelty –The concept of onshore solvent regeneration is genuinely innovative and provides a meaningful contribution to the literature on ship-based carbon capture and port-based carbon management infrastructure. The comparative analysis across multiple routes, solvents, and carbon tax scenarios also enhances the practical relevance of the study. However, the manuscript does not sufficiently emphasize how its contribution advances beyond existing techno-economic studies of SBCC. Prior research has already highlighted issues such as storage requirements and cost implications in ship-based carbon capture systems. I recommend that the authors more clearly situate their novelty in relation to this earlier work and articulate in what specific ways their analysis provides new insights—for example, through the expanded global route coverage, solvent-specific techno-economic integration, or explicit linkage to long-term carbon tax scenarios. Such clarification would help to better establish the originality and added value of this study.

Version 1:

Reviewer comments:

Reviewer #1

(Remarks to the Author)
The revised paper shows significant improvement in quality. However, the author argues that the waste heat recovery for regeneration is negligible, which diverges from most shipboard carbon capture-related studies. This viewpoint requires further in-depth validation. Additionally, LNG accounts for a substantial proportion of ocean-going vessels, and its cold energy utilization potential should not be overlooked. These factors have important implications for the conclusions of this paper and necessitate relevant discussions.

Reviewer #4

(Remarks to the Author)
The manuscript is interesting and worth publishing. The authors have already revised significantly their paper to take into account the good comments from the reviewers.
I have a few comments to add:
_ The authors use the term SBCC, while the vast majority of scientific and industry discussion talk about OCCS (onboard carbon capture and storage). please stick to OCCS...
_ the authors make it sounds like they came up with the onshore regeneration concept for OCCS. However, this is already what Value Maritime is commercialising: https://valuemaritime.com/products-services/. This should be made clearer.
_ From my understanding, PZ is a banned solvent at-sea
_ The authors presents the result per route however it is difficult to understand the results from this. One would expect that presenting the results as a function of the distance would be the way to go as it is the critical parameter behind the route.

Version 2:

Reviewer comments:

Reviewer #1

(Remarks to the Author)
The manuscript has been adequately revised and is suitable for publication

Reviewer #4

(Remarks to the Author)
My comment have been addressed in a proper manner.

Open Access This Peer Review File is licensed under a Creative Commons Attribution 4.0 International License, which permits use, sharing, adaptation, distribution and reproduction in any medium or format, as long as you give appropriate credit to the original author(s) and the source, provide a link to the Creative Commons license, and indicate if changes were made.
In cases where reviewers are anonymous, credit should be given to 'Anonymous Referee' and the source.
The images or other third party material in this Peer Review File are included in the article's Creative Commons license, unless indicated otherwise in a credit line to the material. If material is not included in the article's Creative Commons license and your intended use is not permitted by statutory regulation or exceeds the permitted use, you will need to obtain permission directly from the copyright holder.
To view a copy of this license, visit https://creativecommons.org/licenses/by/4.0/

Rethinking Maritime Carbon Capture: Onshore versus Onboard Solvent Regeneration (manuscript ID: NCOMMS-25-52243A)

Overall response to the Reviewers:

We sincerely thank the reviewers for their constructive and insightful comments, which have greatly strengthened the manuscript. All changes in the revised manuscript are highlighted in red. Below is a summary of the principal revisions made:

1. **Absorption kinetics**: In the original version, solvent performance was evaluated primarily based on equilibrium properties such as absorption capacity and regeneration heat. In the revised manuscript, solvent-dependent absorption kinetics are explicitly incorporated into our absorber design. This is achieved using standard packed-column correlations, which directly influence column sizing and propagate through CAPEX, OPEX, and capture-cost calculations. Full details of the kinetic models, parameter sources, and implementation are provided in the Supplementary Information.
2. **Solvent cost**: We now explicitly account for solvent procurement and make-up costs. Since solvent inventory requirements differ substantially between onboard and onshore regeneration schemes, their economic impact is assessed separately for each case. These contributions are quantified and integrated into the total capture cost.
3. **Route-specific carbon pricing**: In addition to the baseline carbon-tax scenarios, we introduce a route-specific, Emissions Trading System (ETS)-equivalent carbon pricing method. This addition provides more granular and policy-relevant insight into the economic viability of ship-based carbon capture (SBCC) across different shipping routes.
4. **Flexible CO_2 offloading strategy**: Originally, CO_2-rich solvents were assumed to be offloaded at every port of call. The revised manuscript analyzes a more flexible offloading strategy, where only certain ports are identified as mandatory offloading points based on route analysis, while offloading at other ports is treated as optional. This new strategy better reflects real-world port availability and operational constraints within the proposed onshore regeneration scheme.

While these revisions have changed the absolute numerical results, the qualitative trends and core conclusions of the study remain consistent with the original submission. Importantly, the newly added analyses of absorption kinetics, solvent costs, carbon pricing system, and offloading strategies further strengthen the robustness and practical relevance of our work.

Detailed point-by-point responses to each reviewer's comments are provided below.

Reviewer #1

The concept of onshore regeneration was proposed in this paper, and the comparison between the onboard/onshore regenerations was conducted. The paper is interesting while major revisions should be made before publication.

(1) In Abstract section, some description is unclear, e.g., "The onshore scheme is more cost-effective in most scenarios, …SBCC becomes economically viable in 2040 and highly advantageous by 2050, reinforcing the significant long-term potential of the proposed onshore regeneration approach.". Please detail it.

We have revised the Abstract to be more explicit (Page 1 in the revised manuscript):

"*When carbon tax is considered, onshore regeneration is generally more cost-effective across load limits of 10 – 30% in 2030 and 2050, while onboard regeneration remains competitive for a subset of routes in 2040. An extended analysis of 357 global shipping routes indicates that SBCC becomes economically viable by 2040 and broadly applicable by 2050. These conclusions are robust to alternative carbon-pricing formulations and to variations in solvent offloading strategies.*"

(2) "*In Introduction, seldom previous onboard carbon capture work including simulation and experiment research was motioned, most citations (i.e., references 24-39) are just the newspaper report. It is necessary to review the progress technology, energy consumption and discuss the problem of onboard carbon capture.*"

We appreciate the reviewer's comment. Accordingly, the Introduction has been substantially revised to include a focused review of simulation and experimental studies on onboard carbon capture, discussing the capture performance, regeneration energy, cost, and key limitations such as waste-heat availability, low CO_2 concentration in marine exhaust, fuel penalties, and cargo-capacity loss.

Page 2 in the revised manuscript:

"*Previous research has confirmed the technical viability of SBCC using chemical absorption, while also highlighting its substantial energy and cost challenges. For instance, MEA-based systems have achieved 90% CO_2 capture on cargo ships, with reported regeneration energies of 3.85 GJ·tonne^{-1} CO_2 and avoidance costs of 163 €·tonne^{-1} CO_2.[24] Another system-level optimization study has been shown to reduce capital expenditure by 19% and lower the CO_2 avoidance cost to ~236 €·tonne^{-1} CO_2.[25] Beyond MEA, advanced solvents have also been investigated. Piperazine (PZ)*

enables 90% capture on a 1280 kW vessel at a cost of ~207 €·tonne^{-1} CO_2.[26] Blended amines (MDEA-PZ) can achieve ~3.3 GJ·tonne^{-1} CO_2 for the same capture rate,[27] while an optimized MDEA-PZ configuration could increase capture efficiency to 94.7%.[28] Similarly, TEPA-MEA blends increase CO_2 loading by 7% and reduce desorption heat duty by 18.1% compared to MEA.[29] Collectively, these studies indicate that solvent selection and process design are critical factors in SBCC performance.

In parallel, many companies are actively promoting research and development in this area.[30-42] In January 2024, the EVER TOP container ship completed a trial voyage equipped with an onboard CO_2 capture system,[43] and Seabound demonstrated onboard mineralization capturing roughly 1 tonne of CO_2 per day on a 3,237 TEU vessel.[44] Such demonstrations provide important proof-of-concept.

Despite being promising, SBCC is still in its early stages[6,45]. A key limitation in many studies is the assumption that exhaust-gas waste heat can fully meet the energy demand of solvent regeneration.[24-27] However, practical analyses reveal that the recoverable exhaust heat is insufficient,[24,46,47] such that achieving deep emission reductions (70–90%) would increase a ship's total fuel consumption by 70–100% relative to a baseline vessel without carbon capture.[21] Moreover, industry practice indicates that most exhaust heat is already allocated to essential onboard services (e.g., fuel heating and auxiliary systems), leaving little residual energy for SBCC use.[48] Capture efficiency under real operating conditions is another concern. For example, the EVER TOP demonstration captures approximately 44,000 tonnes of CO_2 per year, corresponding to an estimated annual capture rate of only 27%.[49] This issue is exacerbated by the low CO_2 concentration in ship exhaust (~4.8 %),[40] which lies at the lower end of post-combustion capture applications (3–20 vol%)[50] and leads to a reduced thermodynamic driving force and higher specific energy consumption.[51] Finally, the added weight of captured CO_2 and storage tanks diminishes cargo-carrying capacity, which is mentioned by previous studies but the actual operation and economic impact have never been quantitatively evaluated.[21,24]"

(3) "*The simulation method, software, model, parameters for CO_2 capture, regeneration and liquefaction should be detailed in the manuscript, and the accuracy verification of the simulation should be provided.*"

We thank the reviewer for this important suggestion. The simulation software, model, parameters, and validation procedures have been described in detail in ***Supplementary Notes 3, 4, and 5*** now.

Given the complexity of the process modelling (regeneration, liquefaction and cost integration) and the large number of route- and solvent-specific parameters, including all details in the main text would substantially exceed length limits. Supplementary Information describes all calculation details.

(4) "*In the reviewer's opinion, the regeneration heats for PZ, NE-2 AND DES (in Table 2) are not accurate. Their regeneration heats are ~50% lower that of MEA, it is difficult to reach so low value unless coupling with energy-saving processes. Please check it.*"

We thank the reviewer for carefully examining the regeneration heats. In the revised manuscript, the regeneration heats for all solvents have been carefully re-evaluated. The updated values are summarized in the revised Table 2 (Page 21).

Table 2. Solvent Information

Solvent type	Solvent abbreviation	Regeneration heat ($GJ{\cdot}t^{-1}$ CO_2)
Amine solvent	MEA	4.38 (4.6 in original submission)
Amine solvent	PZ	3.12 (3.2 in original submission)
Water-lean solvent	NE-2	2.54 (2.2 in original submission)
Deep eutectic solvent	DES	3.57 (3.2 in original submission)

For MEA and PZ, the regeneration heats are re-estimated using a validated surrogate model specifically adapted to ship exhaust conditions (4.8 vol% CO_2). Compared with typical land-based post-combustion carbon capture, these values are slightly higher due to the lower CO_2 partial pressure in the ship flue gas. For NE-2 and DES, no comparable surrogate or ship-specific reboiler-duty models are currently available. Their regeneration heats are therefore estimated from published experimental or pilot-scale data, using explicit corrections to account for ship-exhaust conditions, as detailed in ***Supplementary Note 4***.

Although the revised regeneration heats changed the capture costs relative to our original submission, the overall trends remain unchanged.

(5) "*As for the total SBCC cost C_{SBCC} (USD) for both onboard and onshore regeneration schemes, please clarify the parameter of cargo loss, what's the difference between onboard and onshore regeneration scheme?*"

In short, cargo loss represents the economic penalty incurred when the weight of the SBCC system reduces a vessel's available cargo carrying capacity.

For the onboard regeneration scheme, the SBCC load comprises the liquefied CO_2 captured and the weight of the onboard CO_2 storage tank. Because the captured CO_2 is stored in liquid form (20.4 bar and −28.9 °C), the tank is designed to withstand cryogenic and pressurized conditions. Therefore, it is quite heavy. These weights directly reduce the available cargo capacity, and the corresponding cargo-loss penalty is explicitly included in C_{SBCC}.

For onshore regeneration, the ship only needs to store CO_2-rich solvent at near-ambient temperature and pressure, so the tank itself is lightweight. However, because the total usage of solvents is large, the cumulative mass of the rich solvent still represents a notable share of the payload. As a result, although the tank weight is negligible, the overall cargo-loss penalty for onshore regeneration remains considerable—particularly for long-distance routes or under high solvent-to-CO_2 ratios.

The detailed mathematical formulation of cargo loss for both schemes is provided in the ***Methods*** section and ***Supplementary Notes 7, 8, 11, 13 and 14.***

(6) "*The utilization waste heating and cold energy is important for the onboard regeneration as described in the references 40-42, it is necessary to discuss this topic in the manuscript.*"

We thank the reviewer for this insightful comment. Indeed, the utilization of waste heat and cold energy is often highlighted in literature 40[1] and 42[2] However, after carefully examining both the published studies and practical engineering data, we conclude that such integrations are impractical for diesel-fueled ships under real operating conditions. The reasons are summarized below:

First, cold-energy integration is not available for diesel-fueled vessels. Cold-side integration (e.g., using LNG vaporization to cool and liquefy CO_2) is feasible only on LNG-fueled vessels.[1] Diesel-fueled ships lack this cold source.

Second, the use of engine exhaust heat for solvent regeneration is often idealized in literature but severely constrained in practice. For example, Feenstra et al. report 90% CO_2 capture rate if exhaust heat fully supplies the stripper reboiler duty.[1] Luo & Wang found that the heat requirement for 90% capture rate cannot be met even using all steam generated by the waste-heat recovery system (economizer). They therefore route flue gas directly to the reboiler to provide more heat.[2] These studies all overlooked the following key operational constraints:

- On diesel- and especially HFO-fueled vessels, economizer-generated steam is essential for fuel heating (to reduce its viscosity for pumping and injection) and for boiler feedwater preheating. These functions already consume a substantial fraction of the recoverable exhaust heat, leaving limited headroom for solvent regeneration.[3]
- Even if all economizer steams are hypothetically diverted to the CO_2 capture system, its capacity is typically insufficient to meet the solvent regeneration duty.[1-5] As a result, SBCC implementations commonly require 70–100% additional fuel consumption relative to a baseline vessel without CO_2 capture.[6]
- Ships rarely operate continuously at full load. Under reduced loads or in colder climates, both exhaust temperature and mass flow decrease, further reducing the amount of recoverable heat. As a result, exhaust heat becomes intermittent and is unsuitable for providing the stable energy required for solvent regeneration.[3]

In summary, while theoretical studies (Refs. 40[1], 42[2]) demonstrate potential for heat and cold-energy integration, practical deployment on diesel-fueled vessels is severely limited by energy availability for essential ship services, capacity and space constraints, and variable operating conditions. We therefore conclude that relying on auxiliary boiler steam is currently the most technically and operationally viable approach for solvent regeneration.

To address this point, we have added some statements in the revised Introduction part (Page 3).

"*Despite being promising, SBCC is still in its early stages*[6,45]. *A key limitation in many studies is the assumption that exhaust-gas waste heat can fully meet the energy demand of solvent regeneration.*[24-27] *However, practical analyses reveal that the recoverable exhaust heat is insufficient,*[24,46,47] *such that achieving deep emission reductions (70–90%) would increase a ship's total fuel consumption by 70–100% relative to a baseline vessel without carbon capture.*[21] *Moreover, industry practice indicates that most exhaust heat is already allocated to essential onboard services (e.g., fuel heating and auxiliary systems), leaving little residual energy for SBCC use.*[48]"

(7) "*The conclusions should be more specific and sufficient to provide the key discovery.*"

We thank the reviewer for this comment. In response, we have thoroughly revised the Conclusions section to make it more specific and comprehensive (Page 17).

"Our analysis underscores that practical SBCC systems design should emphasize feasible load limits—preferably around 20% of a ship's net tonnage—rather than targeting fixed capture rates. The capture cost is evaluated through a comprehensive analysis that explicitly incorporates capital investment, solvent regeneration, CO_2 conditioning, solvent inventory and losses, as well as cargo-capacity loss. Among solvents analyzed, NE-2 emerges as optimum for onboard regeneration due to its low regeneration heat requirement, while DES characterized by superior CO_2 absorption capacity performs best in onshore regeneration scenarios.

When integrating carbon tax implications, the DES onshore regeneration scheme consistently emerges as economically advantageous for smaller vessels (TEU < 6,000) across all projected timeframes. For larger ships, the onshore scheme remains preferable under both 2030 and 2050 carbon tax scenarios, while the NE-2 onboard regeneration scheme becomes competitive for approximately half of the ships in 2040.

Unit penalty analyses further indicate that SBCC implementation remains economically unattractive at the 2030 carbon tax (US$102.2). However, rising carbon taxes progressively enhances its economic viability, with SBCC becoming completely cost-effective by 2050 (US$205.9)—a conclusion further supported by an extended analysis on additional 357 global Maersk shipping routes. Notably, carbon capture technology will continue to advance over time. While this study does not account for its learning curve, doing so would further enhance SBCC's advantages, potentially accelerating its large-scale deployment.

The robustness of the onshore regeneration scheme is confirmed under alternative strategies, including an ETS-equivalent carbon tax system and a flexible solvent offloading strategy, with both yielding results consistent with the base-case analysis.

Several limitations of this study should be acknowledged. First, although absorber sizes are determined using standard industrial packed-column design methods, the capital costs for onboard SBCC equipment are not derived from detailed process simulations. This may lead to an under- or overestimation of actual capital expenditure. Additionally, the economic evaluations fix fuel prices and freight rates and exclude investments in shore-side supporting infrastructure, which may potentially change the evaluated scheme preferences. Furthermore, uncertainties persist regarding the production cost and long-term operational stability of novel solvents, particularly NE-2 and DES. Finally, projections of future carbon tax rates and emission factors introduce further uncertainty."

Reviewer #2:

Overall comment: While the study discusses a very important topic in on-board carbon capture (on-board versus on-shore solvent regeneration evaluating several solvent systems), the study's methodology fails to consider several important aspects to properly design and evaluate an on-board/on-shore capture system for the different solvents considered. For example, the solvent selection argumentation is thin, and a tiny selection of performance parameters is used, while important aspects like solvent kinetics, which partly determines absorber dimensions, are completely neglected. Instead, for the CAPEX calculation, a fixed factor is applied to the total system cost correlation to determine absorber CAPEX, and this approach seems to be the same for each solvent. Additionally, there is little discussion on the selection of the regeneration energy demands of the different solvents considered in this study, while this has a high impact on the results. These are too big simplification that affect the calculations and conclusions greatly, and limits the usefulness of the study. This is reflected in the selection of the DES solvent (only based on high capacity and relatively low reboiler duty) as the optimal solvent in the final study, while the reference paper used for DES does not even seem to discuss kinetics. It is noted that a limitation on equipment costing is added to the conclusions, but this seems insufficient to account for the inaccuracy that these simplifications are giving. More detailed simulations and cost calculations for all solvent systems separately is at least required to increase the usefulness of the study.

Overall response: We thank the reviewer for all the insightful comments. We agree that the original manuscript relied on certain simplifications and assumptions regarding solvent selection, absorber design, CAPEX estimation, and regeneration energy calculation, which indeed require more careful justification. In the revised manuscript, these limitations have been explicitly addressed by (1) strengthening the rationale for solvent selection, (2) incorporating solvent-specific absorption kinetics into the absorber design, (3) recalculating CAPEX based on solvent- and route-dependent absorber sizing rather than fixed scaling factors, and (4) revising the regeneration heat values for all solvents using more accurate correlation models or experimental data. The updated method, revised model equations, and detailed calculations are documented in ***Supplementary Notes 4, 9, and 10***. A point-by-point response to each of the reviewer's specific concerns is provided below.

Specific comments:

(1) "*63 – This statement requires quantification, explanation and referencing*"

We thank the reviewer for this valuable comment. The original statement was based on our own estimation derived from publicly reported data of the EVER TOP project. According to the project report, approximately 44,000 tonnes of CO_2 were captured annually.[7] To concretize this value, we estimated the ship's annual CO_2 emission using the same method described in Supplementary Note 2.

$$m_{CO_2} = \eta \cdot SFOC \cdot MCR \cdot EF \cdot t = 161641.4 \; tonne \; CO_2 \; yr^{-1}$$

where the ship's main engine has an MCR of 53,250 kW and an assumed annual operation time of 300 days.

From this, the estimated capture rate is:

$$CR = \frac{44000}{161641.4} \times 100\% \approx 27\%$$

Based on this analysis, we concluded that the EVER TOP project currently achieves a limited capture rate relative to its total emissions.

Following the reviewer's suggestion, we have revised the corresponding sentence (Page 3).

"*Capture efficiency under real operating conditions is another concern. For example, the EVER TOP demonstration captures approximately 44,000 tonnes of CO_2 per year, corresponding to an estimated annual capture rate of only 27%.*[49]"

(2) "*64 – Is there a reason only a steel plant is mentioned here, these CO_2 concentrations are very high, already in the range where other technologies than chemical absorption become favorable. I think it would be more fair to compare the CO_2 concentration towards other post-combustion applications in the 5-20 vol% range (e.g. gas-fired power systems, waste-to-energy, coal-fired).*"

We thank the reviewer for this insightful comment. We agree that comparing ship exhaust solely with steel-plant flue gas, which contains unusually high CO_2 concentrations, is not representative of typical post-combustion conditions.

Following the reviewer's suggestion, we have revised the sentence to compare ship exhaust with the more relevant post-combustion concentration range (Page 3).

"This issue is exacerbated by the low CO_2 concentration in ship exhaust (~4.8 vol%),[40] which lies at the lower end of post-combustion capture applications (3–20 vol%)[50] and leads to a reduced thermodynamic driving force and higher specific energy consumption.[51]"

(3) "*68 – doesn't have to increase always. If enough heat can be recovered from the flue gases, the fuel penalty might be limited. Is any heat recovery analysis included in the study?*"

Indeed, if sufficient heat can be recovered from exhaust gases, there would be no need for additional fuel consumption. However, based on published studies and available engineering evidence, exhaust-gas waste heat is not practically available for solvent regeneration under realistic marine operating conditions, and therefore exhaust-heat integration has not been considered in this study. The rationale is as follows:

1. On diesel- and HFO-fueled vessels, waste-heat recovery system (i.e., economizer)-generated steam is already used for essential ship functions, including fuel heating (to reduce viscosity and ensure proper injection) and boiler feedwater preheating. These mandatory services consume a substantial fraction of the recoverable exhaust heat, leaving little practical margin for solvent regeneration.[3]

2. Even if all recovered heat are hypothetically diverted to the CO_2 capture system, its capacity is typically insufficient to meet the solvent regeneration duty.[1,2,4,5] As a result, SBCC implementations commonly require 70–100% additional fuel consumption relative to a baseline vessel without CO_2 capture.[6]

3. Ships rarely operate continuously at full load. Under reduced loads or in colder climates, both exhaust temperature and mass flow decrease, further reducing the amount of recoverable heat. As a result, exhaust heat becomes intermittent and is unsuitable for providing the stable energy required for solvent regeneration.

In summary, while theoretical studies may indicate potential for heat integration, practical deployment on diesel-fueled vessels is severely limited. Auxiliary boiler-generated steam is currently the most technically and operationally viable approach for providing heat for solvent regeneration.

To address this point, we have added some explanations in the revised manuscript (Page 3).

"*Despite being promising, SBCC is still in its early stages.[6,45] A key limitation in many studies is the assumption that exhaust-gas waste heat can fully meet the energy demand of solvent*

regeneration.[24-27] However, practical analyses reveal that the recoverable exhaust heat is insufficient,[24,46,47] such that achieving deep emission reductions (70–90%) would increase a ship's total fuel consumption by 70–100% relative to a baseline vessel without carbon capture.[21] Moreover, industry practice indicates that most exhaust heat is already allocated to essential onboard services (e.g., fuel heating and auxiliary systems), leaving little residual energy for SBCC use.[48]"

(4) "*74 – Is liquefaction always required? What if the shore systems are connected to pipeline transportation? Suggesting to change liquefaction to conditioning.*"

We thank the reviewer for this insightful suggestion. We agree that liquefaction is not always required and that conditioning is more suitable to describe the preparation of captured CO_2 for downstream handling. Accordingly, the terminology has been revised throughout the manuscript.

For onboard regeneration, CO_2 is conditioned to a liquid state stored in tanks.[1] This provides a high storage density and is also compatible with port-side offloading systems.[8] In this study, a representative onboard conditioning condition of 20.4 bar and −28.9 °C is adopted,[9] showing a highest storage density and corresponding to an energy demand of 0.71 GJ $tonne^{-1}$ CO_2, as detailed in ***Supplementary Note 3***.

For onshore regeneration, CO_2 conditioning depends on the subsequent transport mode. For large-scale continuous transport, pipeline transmission is preferred. In this case, CO_2 is conditioned to a liquid or supercritical state.[10] In our revised manuscript, CO_2 is compressed to 120 bar and 25 °C, where it is a compressed liquid suitable for pipeline transport.[10] The corresponding conditioning energy cost is calculated as 0.43 GJ $tonne^{-1}$ CO_2 ***(Supplementary Note 3)***.

Although both cases result in liquid-phase CO_2, the underlying processes differ. Liquefaction refers specifically to low-temperature phase change, whereas conditioning generally encompasses compression, cooling, and phase control required to prepare CO_2 for its subsequent transport. We therefore adopt conditioning as the terminology throughout the revision.

(5) "*117 – This seems to be calculated assuming 30 wt% MEA. Should be mentioned.*"

Thank you for pointing out this oversight. We agree that the calculation is based on 30 wt% aqueous MEA, and we have revised the text accordingly.

(6) "*178 – Absorption capacity is not the only metric that determines whether a solvent is a good candidate. Kinetics for example are very important for determination of the absorber dimensions, something this study does not cover.*"

We appreciate the reviewer's emphasis on the critical role of solvent kinetics. In the revised manuscript, this limitation has been explicitly addressed. The absorber sizing is now performed using a rate-based packed-column design, in which solvent-specific kinetic and transport properties are incorporated through established mass transfer correlations. Specifically, liquid- and gas-phase mass transfer coefficients, effective interfacial area, and height-of-transfer-unit (HTU/NTU) relationships are evaluated as functions of solvent viscosity, diffusivity, surface tension, and operating conditions. These calculations are combined with hydraulic constraints, pressure-drop limits, and practical packing segmentation, consistent with industrial absorber design practice.

This revised approach directly captures differences in mass-transfer kinetics and transport behavior among solvents and replaces the previous capacity-based simplification. The complete methodology is described in ***Supplementary Note 9***, and the resulting solvent- and route-specific absorber dimensions are provided in ***Supplementary Data 4***.

(7) "*245 – It is understood that using all simplifications, DES comes out as the best solvent (high capacity, good regeneration energy), but this fails to take into account a lot of aspects required for designing a capture system with this solvent. An example: what absorber height would be required to reach the CO_2 capacity in the solvent considered in this study?*"

We appreciate the reviewer's question regarding the practical feasibility of DES operation and the associated absorber dimensions.

In the revised manuscript, the performance of DES is more comprehensively assessed using standard rate-based packed-column design procedures. These calculations explicitly incorporate solvent-specific physical and transport properties, gas-liquid mass transfer correlations, route-dependent flue-gas flow rates, and practical design constraints including hydraulic limits, pressure-drop constraints, and packing segmentation.

For the DES evaluated in this study, the required absorber heights to achieve the target capture performance are calculated to lie in the range of approximately 10–16.5 meters, depending on route conditions and load limits. These values fall well within the range commonly reported for large-scale industrial CO_2 absorbers,[1,2,11] demonstrating the technical feasibility of DES-based SBCC.

The complete sizing methodology and route-specific absorber heights are provided in ***Supplementary Note 9*** and ***Supplementary Data 4***, ensuring transparency and reproducibility.

(8) "*340 – This is only true if HFO is assumed as the fuel. Is this the case?*"

The value of approximately 3.1 tonnes of CO_2 produced per tonne of fuel consumed is not specific to heavy fuel oil (HFO); rather, it is a general stoichiometric property of hydrocarbon fuels commonly used in marine diesel engines. To illustrate this, we provide below the combustion equations and CO_2-yield calculations for two different fuel types.[9]

1. #1 light diesel fuel (approximated as $C_{12}H_{26}$, MW = 170 g/mol)

Combustion reaction:

$$C_{12}H_{26} + 18.5O_2 \rightarrow 12CO_2 + 13H_2O$$

Weight of CO_2 produced after combusting 1 mole fuel:

$$12 \times 44 = 528\ \ \mathrm{g}$$

CO_2-to-Fuel mass ratio:

$$\frac{528\ \ \mathrm{g}}{170\ \ \mathrm{g}} = 3.10$$

2. Heavy Fuel Oil (approximated as $C_{14}H_{30}$, MW = 198 g/mol)

Combustion reaction:

$$C_{14}H_{30} + 22.5O_2 \rightarrow 14CO_2 + 15H_2O$$

Weight of CO_2 produced after combusting 1 mole fuel:

$$14 \times 44 = 616\ \ \mathrm{g}$$

CO_2-to-Fuel mass ratio:

$$\frac{616\ \ \mathrm{g}}{198\ \ \mathrm{g}} = 3.11$$

As shown above, for both #1 light diesel and heavy fuel oil, complete combustion yields approximately 3.1 kg of CO_2 per kg of fuel. Therefore, the emission factor used in our study is a general and widely accepted approximation for conventional marine hydrocarbon fuels (e.g., HFO, MDO, MGO), not an assumption restricted to HFO alone.

(9) "*362 – The choice to evaluate MEA and PZ seems obvious, given the broad information basis for both solvents. The selection criteria seems to be quite thin for NE-2 and DES. The NE-2 regeneration duty seems to be based on a single small pilot study. Regarding DES, at equilibrium these high loading conditions might be achieved, but is it realistic to assume this is possible on a vessel (and what absorber height would be required)? A more detailed discussion on solvent selection is required to justify selection of these solvents.*"

We thank the reviewer for this constructive comment regarding solvent selection and practical feasibility. We agree that the inclusion of NE-2 and DES requires clearer justification, particularly concerning data robustness and shipboard operability. In response, we have substantially expanded the discussion on solvent selection in the revised manuscript to address these specific concerns.

The reason for evaluating MEA and PZ is obvious since they are well established solvents. The rationale for including NE-2 and DES arises from the unique constraints of ship-based carbon capture (SBCC).

Unlike land-based systems, **onboard regeneration** relies primarily on fuel-derived energy rather than low-cost steam or waste heat (as discussed in Comment 2). Consequently, regeneration energy becomes a dominant factor for both operating cost and life-cycle emissions. Therefore, solvents with a reduced regeneration heat are of particular interest. NE-2 is a representative water-lean solvent that has demonstrated an exceptionally low regeneration energy of about 2.2 $GJ \cdot t^{-1}$ CO_2 in pilot-scale testing.[12] We fully acknowledge that its regeneration data is based on limited pilot studies and this solvent is not commercially mature. However, it is deliberately used as a lower-bound reference to illustrate the potential regeneration-energy reduction. To ensure consistency with ship-exhaust conditions characterized by lower CO_2 partial pressure, we have adjusted the reported regeneration duty using MEA as a benchmark, yielding an estimated regeneration heat of 2.54 $GJ \cdot t^{-1}$ CO_2 for NE-2, which is used in our revised manuscript ***(Supplementary Note 4)***.

In contrast, the primary motivation for including a deep eutectic solvent (DES) is not minimal regeneration energy, but its exceptionally high CO_2 absorption capacity. This property is critical for the proposed **onshore regeneration** scheme, where CO_2-rich solvent must be stored onboard over multiple voyage segments before offloading, making solvent weight and absorption capacity key design preferences. DESs have recently emerged as promising alternatives to ionic liquids, offering high CO_2 solubility while avoiding drawbacks, such as poor kinetics, high cost, and excessive

viscosity.[13] Several DES systems based on protonated amines and hydrogen-bond donors have been reported to possess very high CO_2 uptakes under ambient conditions.[14] In this study, we select a aqueous [MEACl][EDA] DES with a reported CO_2 capture capacity high as 32.15 wt%.[15] Crucially, we do not assume instantaneous attainment of equilibrium under shipboard conditions. As the reviewer rightly noted, absorber dimensions were determined following standard industrial packed-column design practice. The resulting absorber dimensions fall within the typical range reported for large-scale industrial CO_2 absorbers. Details are provided in ***Supplementary Note 9 and Data 4***.

In summary, NE-2 and DES are selected as representative solvents from two distinct classes that bound the performance space most relevant to SBCC. NE-2 illustrates the lower limit of regeneration energy achievable with water-lean solvents, while DES represents a high-absorption-capacity solvent suited for onshore regeneration.

We added some explanations on solvent selection (Page 21 in the revision):

"*We evaluated four solvents with distinct physicochemical properties to span a broad and most relevant performance space for ship-based carbon capture applications. Monoethanolamine (30 wt% MEA) is included due to its extensive industrial deployment.*[56] *Piperazine (PZ) is selected as a representative second-generation aqueous amine, offering faster kinetics and a lower reported regeneration heat compared to MEA.*[57] *Beyond conventional aqueous amines, advanced solvent classes were considered to reflect the specific constraints of SBCC. Water-lean solvents have attracted increasing interest due to their reduced regeneration duty arising from lower water content and higher cyclic CO_2 capacity. In this context, the water-lean solvent NE-2 composed of 2-(ethylamino)ethanol (EMEA), 1-methyl-2-pyrrolidinone (NMP), and water has demonstrated a regeneration heat as low as 2.2 GJ tonne^{-1} CO_2 in pilot-scale studies, and is therefore included as a representative low-regeneration-energy solvent.*[58] *Notably, to keep consistency with ship-exhaust conditions, the regeneration heat of NE-2 is adjusted in this study, as detailed in Supplementary Note 4. Deep eutectic solvents (DESs) are considered as a distinct solvent class characterized by high mass absorption capacity, which can be potentially beneficial for our proposed onshore regeneration strategy involving long-time solvent storage prior to offloading. In this study, 60 wt% aqueous [MEA·Cl]:[EDA] DES (1:6) (denoted as DES) is selected as a representative system, exhibiting a reported CO_2 mass loading of 32.15 wt%.*[59]

(10) "*428 – In the supplementary information, it is explained that the total CAPEX is determined using a simple correlation only using the MCR and capture rate. This is a big simplification, especially if the differences between the solvents are not taken into account. It is also assumed that the absorber CAPEX is always a fixed percentage of the total costs, which is again a big simplification, especially considering the inherent different (e.g. in kinetics) between the different solvents.*"

We thank the reviewer for raising this important concern. We agree that the original formulation did not sufficiently account for solvent effects, particularly in absorber design.

In the revised manuscript, this limitation has been explicitly addressed. CAPEX is no longer determined solely from correlations based on the maximum continuous rating (MCR) and capture rate. Instead, as detailed in ***Supplementary Note 9***, absorber dimensions are now derived from route- and solvent-specific packed-column design calculations that explicitly incorporate gas-liquid mass transfer performance, hydraulic constraints, and pressure-drop limits. We completely removed the previous uniform absorber design and fully captured differences in solvent characteristics.

For the onboard regeneration scheme, the absorber cost derived from the above customized design method is used as the scaling basis for the remaining onboard equipment. We retain this top-down scaling approach for the rest of the capture system because a fully bottom-up cost estimation of all onboard capture subsystems, including compressors, heat exchangers, piping, and instrumentation, would require comprehensive solvent-specific datasets encompassing intrinsic reaction kinetics, phase equilibrium models, and complete thermophysical property packages. Such datasets are currently unavailable for emerging solvents such as NE-2 and DES. Nevertheless, since the absorber is the dominant and most solvent-sensitive unit, this approach ensures that solvent-dependent performances are consistently propagated into the overall CAPEX.

For onshore regeneration, the onboard absorber is sized using the same design method. In addition, packing cost is calculated explicitly for each case ***(Supplementary Note 10)***, ensuring that the onboard portion of the CAPEX is fully differentiated across solvents. The remaining onshore regeneration facilities are estimated using land-based reference plants with solvent-specific scaling, resulting in a more physically grounded and differentiated CAPEX estimation compared to the original submission.

Overall, the revised methodology replaces the previous uniform-cost assumption with a solvent- and route-sensitive absorber design, while maintaining a transparent, reproducible, and system-level costing framework appropriate for comparative techno-economic assessment.

(11) "*445 – The cost metrics are based on the mass flow of CO_2 capture captured. This does not seem to take additional emissions into account that are caused by the capture system. CO_2 avoidance, taking these additional emissions into account, should be calculated for a 'real' emission reduction measure. It can be expected that regulatory frameworks will also work with CO_2 avoidance instead of CO_2 captured.*"

We thank the reviewer for raising this important point. We fully agree that additional emissions generated by the capture system must be accounted when assessing the net climate benefit of SBCC. We want to highlight that our calculation already incorporates these additional emissions for both the onboard and onshore regeneration schemes, although the emission accounting methods slightly differ.

For onboard regeneration, all additional emissions from solvent regeneration, CO_2 compression, and liquefaction or conditioning occur on the vessel. These emissions increase the ship's total CO_2 emissions (***Supplementary Note 5***) and are explicitly included in all three cost metrics.

For onshore regeneration, additional emissions from regeneration and conditioning occur onshore (NOT on the ship), outside the ship's emission accounting boundary. According to the current maritime MRV (Monitoring, Reporting and Verification) practices, these emissions should not been included in the ship emission.[16] Even though we do not account for this additional emission in ship emission, the associated energy and all processing costs of onshore regeneration and conditioning are included in the calculation of total capture cost. Moreover, our Total Cost with Carbon Tax (TCT) and Unit Penalty (UP) fully incorporate the cost arising from these additional onshore emissions using the corresponding carbon tax or price. Detailed formulations are given in the Methods section and ***Supplementary Notes 5, 14, 15, and 17***.

In summary, additional emissions arising from SBCC—whether onboard or onshore—are fully incorporated in the cost calculations. The cost metrics are derived on a CO_2-avoidance basis, consistent with the current maritime MRV regulatory frameworks.

(12) "*450 – From this text, it is not clear how CAPEX is annualized and how this is taken into account in the calculations.*"

In the original submission, CAPEX was expressed directly on a per-tonne-of-CO_2-captured basis and therefore did not require explicit annualization. However, in the revised manuscript, we have substantially updated the CAPEX methodology to reflect solvent- and route-specific absorber design,

resulting in distinct capital costs that must be annualized to ensure a consistent techno-economic comparison across cases.

CAPEX is now annualized using a standard chemical-engineering capital-recovery approach. The fixed capital investment (FCI) is converted to an equivalent annual cost using a capital-recovery factor that accounts for the assumed plant lifetime and discount rate. This annualized CAPEX is then combined with operating costs and normalized by the annual amount of CO_2 captured to obtain the specific capture cost.

The complete annualization procedure, including all parameters (lifetime and discount rate) as well as model equations, is provided in ***Supplementary Note 9, Section 12***. The entire calculation workflow has also been clarified to ensure full transparency and reproducibility.

Reviewer #3

This manuscript presents a comparative techno-economic analysis of onboard versus onshore solvent regeneration schemes for ship-based carbon capture (SBCC). The concept of onshore regeneration is original and of high relevance to maritime decarbonization, and the comparative framework across multiple routes, solvents, and projected carbon tax scenarios provides practical value. The study therefore has the potential to make a meaningful contribution to both the academic literature and policy discussions on SBCC deployment.

We thank the reviewer for this thoughtful and encouraging assessment. We greatly appreciate your recognition of the novelty and practical significance of our study.

Nevertheless, there are several issues related to clarity, methodological robustness, and contextualization that need to be addressed to strengthen the manuscript. My detailed comments are as follows:

(1) "*Terminology of load limits – The manuscript uses the term "SBCC load limits" to describe capture capacity in both onboard and onshore regeneration schemes. However, the physical meaning of the load differs substantially between the two cases: onboard regeneration is constrained primarily by the weight of liquefied CO_2 and storage tanks, whereas onshore regeneration is limited by the much larger solvent inventory required to store CO_2-rich solutions. Using the same terminology for both schemes may create ambiguity for readers and obscure these fundamental differences. I suggest the authors adopt distinct or more precise terminology to differentiate the constraints for onboard and onshore regeneration—for example, specifying "CO_2 storage load limit" for onboard systems and "solvent storage load limit" for onshore systems. This would improve clarity and avoid potential misinterpretation.*"

We agree with the reviewer that the physical meaning of the load limit differs between the two regeneration schemes. We have therefore revised the terminology throughout the manuscript. When discussing each scheme individually, we now use the terms "CO_2 storage load limit" for onboard regeneration and "rich-solvent storage load limit" for onshore regeneration. For figures and analyses that apply to both schemes, we retain the generic term "SBCC load limit". This adjustment ensures precise technical communication while maintaining consistent notation across comparative analyses.

(2) "*Load ratio interpretation – The manuscript states that "all schemes achieve relatively low load ratios, averaging 0.25". However, Figure 2b shows that for the onshore regeneration scheme, a significant number of routes exceed this average value, with some load ratios reaching as high as 0.82. This discrepancy suggests that the "average" description may obscure important differences across routes. I recommend that the authors analyze the specific cases with notably higher load ratios, discussing the operational implications and whether such routes remain feasible for onshore regeneration. This additional analysis would provide a more balanced and transparent assessment of the scheme's applicability.*"

We appreciate the reviewer's insightful observation. We agree that a route-averaged load ratio can obscure substantial heterogeneity across individual routes, particularly for the onshore regeneration scheme where certain routes exhibit markedly higher load ratios.

Accordingly, we have revised the manuscript to explicitly highlight the large route-to-route variation and explain the physical drivers behind elevated load ratios. The revised text clarifies that high average load ratios are usually found for ships with small net tonnage and long inter-port transportation times under the onshore regeneration scheme, conditions that lead to a greater accumulation of rich solvent on the ship. We further emphasize that this averaged metric serves solely as a heuristic indicator to guide the selection of representative load-limit values, rather than as a direct feasibility criterion for any specific route.

Page 7 in the revised manuscript:

"*Subsequently, the average SBCC load-to-ship-net-tonnage ratio (ω) at a 90% capture rate is calculated using Eq. 7 and shown in Fig. 2b. This route-averaged ratio varies substantially across routes, reflecting differences in solvent property and regeneration scheme, ship net tonnage, and inter-port transportation time. For example, the ME Eastbound route using NE-2 onshore scheme exhibits an average ratio as high as 0.92, largely attributable to a relatively small vessel (4,250 TEU) and a long average transportation time (5.8 days), which leads to large rich-solvent accumulation on the ship. In contrast, routes such as AE11 westbound, operated by larger vessels (24,034 TEU) with comparable sailing durations (4.5 days), exhibit much lower load ratios. While this average ratio does not reflect any physical reality, as captured CO_2 cannot be redistributed across voyage segments, it serves as a valuable indicator for selecting an appropriate load limit. Overall, most routes cluster at relatively low*

load limits, averaging around 0.25, which aligns with IMO reports indicating that ships typically operate at about 70% of their cargo capacity.[53] *Based on these observations, setting an SBCC load limit of 20% is reasonable to maximize CO_2 capture rate without significantly reducing cargo capacity. For a comprehensive analysis, we additionally evaluate scenarios with 10% and 30% load limits.*"

(3) "*Low capture rate routes –The results show that the capture rate of the onshore regeneration scheme ranges widely from 20% to 82%. However, the manuscript does not discuss which specific routes fall into the lower end of this range, nor whether such low capture rates undermine the practical applicability of the scheme for those cases. To improve the relevance of the findings, I suggest the authors explicitly identify the routes where capture rates are too low to justify onshore regeneration and discuss the potential reasons (e.g., voyage duration, solvent load, ship size). This would help readers better understand the operational boundaries of the proposed technology and avoid overgeneralizing its applicability across all routes.*"

We appreciate this comment highlighting the importance to interpret the low capture rate in a route-specific context. The route exhibiting the lowest capture rates across all four solvents is the ME Eastbound route, for which the onshore regeneration scheme achieves capture rates of about 16%–39%, depending on the solvent (Note: the lower bound of 20% reported in our original submission has been updated to 16%). This route also exhibits the highest average SBCC load ratio (Fig. 2b).

The low capture performance on this route arises from two combined factors: (i) the small vessel size (4,250 TEU), which limits allowable onboard rich-solvent inventory under a fixed load limit; and (ii) the long inter-port transportation time, which leads to substantial CO_2 generation (accumulated in the rich-solvent) before offloading is possible. As a result, the solvent storage limit is reached early in the voyage, forcing capture to be suspended for the majority of the ship sailing time.

These findings indicate that onshore regeneration could yield an extremely low overall capture rate using certain solvents, and that routes operated by small ships and long voyage segments represent the lower operational boundary of applicability. Importantly, such cases are explicitly identified in our analysis and are not obscured by route-averaged metrics.

We have clarified these points in the revised manuscript to prevent overgeneralization and to better delineate the operational window of the onshore regeneration scheme.

Page 9 in the revised manuscript:

"*Capture rates at the lower end of the distribution occur on routes characterized by small vessel sizes and long inter-port transportation times (e.g., the ME Eastbound route discussed in Fig. 2b), which also tend to exhibit elevated average SBCC load ratios, causing the solvent storage limit to be reached early in the voyage segment.*"

(4) "*Carbon pricing mechanisms –The manuscript provides a detailed analysis of how future carbon tax levels influence the economic viability of SBCC schemes. However, in practice, carbon pricing in the maritime sector is not limited to taxation—emissions trading systems (ETS), such as the EU ETS, are already being extended to international shipping and are expected to play an increasingly significant role. Since ETS mechanisms differ from carbon taxes in design and market dynamics (e.g., allowance trading, price volatility, regional disparities), their impacts on SBCC adoption could be materially different. I recommend that the authors at least discuss the potential influence of carbon trading schemes on SBCC economics, or provide a sensitivity analysis incorporating carbon allowance prices. This addition would broaden the policy relevance of the study and make its conclusions more robust under real-world regulatory frameworks.*"

We thank the reviewer for this insightful comment. Carbon pricing is the primary driver of SBCC, and we fully agree that emissions trading systems (ETS)-based regulations are becoming increasingly relevant for maritime emissions.

The EU ETS represents the most established framework that can be applied to international shipping.[17] Notably, unlike other industrial sectors, shipping does not receive free allowance allocation, requiring operators to purchase allowances to cover their emissions.[17] Although ETS prices differ conceptually from carbon taxes, both ultimately reflect the marginal cost of emitting one tonne of CO_2. Outside the EU, however, only a very limited number of ETS are relevant to maritime emissions (e.g., the Shanghai Pilot ETS and New Zealand ETS).[18] Compared to the EU ETS, these emerging systems differ substantially in scope, stringency, and price formation. Considering the realities mentioned above, projecting a credible global ETS allowance price is highly uncertain and not well suited for long-term techno-economic analysis.[18]

To capture ETS-style incentives without relying on uncertain allowance-price forecasts, we introduced a route-specific ETS-equivalent CO_2 pricing system based on region-dependent CO_2 price trajectories reported by the IEA under the net-zero-emission pathway. Specifically, each port is assigned an ETS-equivalent CO_2 price according to its geographic region. Each voyage segment

inherits the mean price of its departure and arrival ports. Finally, a sailing-time-weighted average is calculated to determine a single ETS-equivalent CO_2 price for each complete route. This approach aligns with the voyage-based allocation logic employed in the EU ETS,[19] while enabling that geographically varied carbon-pricing regimes are consistently reflected for both onboard and onshore regeneration schemes. Under the onshore scheme, emissions generated in port are priced according to the regional CO_2 price applicable at that port.

The detailed calculation procedure is provided in ***Supplementary Note 15***. Extended Data Fig. 3 summarizes the results using the new route-specific CO_2 prices. While absolute values differ from those obtained previously under the uniform carbon-tax assumption, the primary techno-economic trends remain unchanged, confirming the robustness of our conclusions. For better comparison, the study in our original submission using the uniform carbon-tax framework is retained.

Page 24 in the revised manuscript:

"***Carbon tax.*** *Two different carbon taxes are used in this paper. First, a global carbon tax level is calculated using a weighted average based on each region's share of global emissions (Supplementary Table 22) and their respective carbon tax rates (Supplementary Table 23). This approach captures regional disparities in emissions, yielding an estimate tailored to the global shipping industry. As a result, the projected carbon tax rates for 2030, 2040, and 2050 are US$102.2, US$168.8, and US$205.9, respectively. Second, to account for geographically varied carbon pricing regimes, including emissions trading systems (ETS), we introduce a route-specific ETS-equivalent carbon tax system. Specifically, each port is assigned with a* CO_2 *price taken from the IEA 2050-Net-Zero-Emission scenario according to its geographic location. Segment-level ETS-equivalent carbon taxes are then derived based on the carbon prices of the two connected ports, and these values are incorporated directly into the route-level total cost calculations for both onboard and onshore regeneration schemes. Meanwhile, a sailing-time-weighted average of all segment taxes is computed to obtain a single route-level ETS-equivalent carbon tax (Supplementary Table 25), which is used for comparison with unit penalty to judge the economic viability of SBCC for each route. Detailed calculations are presented in Supplementary Note 15.*"

Page 12 in the revised manuscript:

"*To assess policy relevance under realistic regulatory conditions, we further evaluate the cost metrics under a route-specific ETS-equivalent carbon tax system. The resulting trends (Extended Data Fig. 3) closely mirror those obtained under the former uniform carbon-tax scenario, demonstrating the robustness of our conclusions across different carbon-pricing frameworks.*"

Page 34 in the revised manuscript:

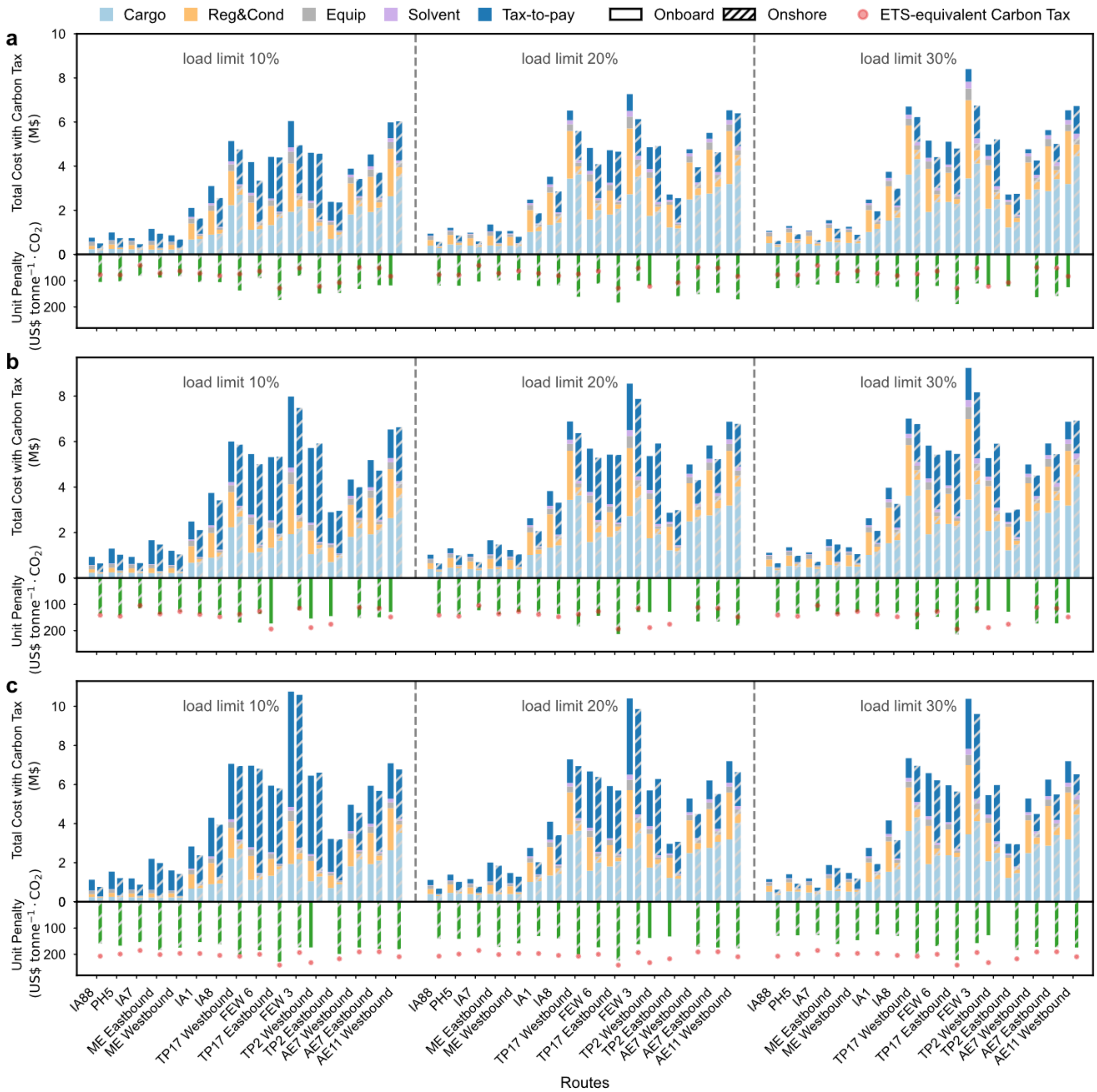

Extended Data Fig. 1 | Total cost of SBCC and unit penalty across 16 routes under route-specific ETS-equivalent carbon tax scenarios. *Subfigures (**a–c**) present results for the policy years 2030, 2040 and 2050, respectively. Within every subfigure, the upper chart shows the total cost with carbon tax (TCT) at three SBCC load-limit constraints—10%, 20% and 30%. Stacked bars partition the TCT into cargo-loss cost (light blue), regeneration plus conditioning cost (orange), fixed equipment cost (grey), solvent cost (purple) and carbon-tax expenditure (dark blue). Solid bars correspond to the onboard-regeneration scheme using NE-2, whereas diagonally hatched bars represent the onshore-regeneration scheme using DES as the solvent. Routes are ordered by vessel sizes. The lower chart in each subfigure compares the ETS-equivalent carbon-tax value (red dot) with the unit penalty imposed by the more economical regeneration scheme.*

(5) "*Solvent management strategies – In the economic evaluation, the onshore regeneration scheme is shown to require substantially larger volumes of solvent than onboard regeneration. However, the manuscript does not clarify how solvent management is envisioned in practice—whether ships would replace the CO_2-rich solvent with new solvent at each port call, or whether the same solvent would be regenerated onshore and then reloaded. These two approaches have very different implications for cost, logistics, and sustainability. Moreover, solvent cost assumptions are not explicitly discussed, even though solvent prices and availability could significantly influence the economic viability of the onshore scheme. I recommend that the authors elaborate on solvent replenishment strategies and provide at least a qualitative discussion of how solvent costs might affect the overall economic assessment.*"

We thank the reviewer for recommending that we clarify solvent management strategies and their cost calculations.

Solvent management strategy

For the onshore regeneration scheme, we assume that at each port the ship offloads CO_2-rich solvent and simultaneously receives an equivalent volume of lean solvent. Operationally, this is equivalent to regenerating the same solvent onshore and returning it to the ship, but without waiting for regeneration. This exchange-based strategy also avoids repeated purchases of fresh solvent at each port. Under this configuration, some routes require a large initial solvent inventory. To provide a realistic estimate of solvent cost, we assume that each ship initially purchases enough solvent to satisfy the CO_2 capture demand of its longest transportation segment between two adjacent ports.

For onboard regeneration, solvent is continuously cycled between the absorber and regenerator, making its initial inventory much smaller than that required for onshore regeneration. In our revised manuscript (***Supplementary Note 12***), solvent inventory is quantified from column holdup and specified liquid residence time.

Solvent cost

Consistent with the above strategy, we have added solvent cost into the economic assessment in the revised manuscript for both schemes through two components:

1. initial solvent inventory, which is substantial for the onshore regeneration scheme but minimal for the onboard scheme due to the closed absorption-desorption cycle;
2. make-up solvent required to compensate for operational losses due to degradation, oxidation, evaporation, and leakage.

Solvent prices and loss rates are discussed, and detailed calculation procedures are provided in ***Supplementary Note 12***. Notably, while solvent inventory requirements differ markedly between the two schemes, solvent-related costs contribute only a modest fraction of total capture cost compared to energy consumption and cargo-loss penalties (see Figure 5). As a result, the main conclusions of the study remain unchanged.

We have clarified solvent cost calculations in the revised manuscript (Page 23).

"***Solvent Cost.*** *Solvent cost is an important component of the economic assessment for SBCC. In this study, the cost is composed of two parts: (i) the initial solvent inventory required for system operation, and (ii) make-up solvent to account for degradation and losses during operation. All related assumptions, pricing data, and calculation procedures are provided in Supplementary Note 12.*"

Page 24 in the revised manuscript:

"***Cost for onboard and onshore regeneration schemes.*** *The total SBCC cost* C_{SBCC} *(US$) for both onboard and onshore regeneration schemes include several key components: fixed equipment, regeneration, conditioning, solvent and cargo loss.*

$$C_{SBCC} = C_{equip} + C_{regeneration} + C_{conditioning} + C_{solvent} + C_{cargo\ loss} \quad (1)$$

(6) "*Uncertainties in solvent performance – The analysis heavily relies on performance assumptions for emerging solvents (NE-2, DES). However, the uncertainties in solvent stability, degradation, and long-term operability are only briefly acknowledged. A sensitivity analysis on solvent performance (e.g., variation in regeneration heat, degradation rates) would greatly strengthen the robustness of the conclusions.*"

We thank the reviewer for raising the importance of performance uncertainties associated with the emerging solvents NE-2 and DES. We agree that their stability and degradation behavior are less established than MEA and PZ.

In our economic model, we explicitly account for solvent degradation and losses by including a recurring make-up cost for each solvent. This cost represents the additional solvent required to compensate for degradation and leakage during continuous operation, and it is added to the one-time, purchase cost of the initial solvent inventory. For each solvent, the make-up rate is set according to the best available literature report (expressed as "how much solvent make-up is required for capturing one tonne of CO_2"). The detailed calculations are provided in ***Supplementary Note 12***. We find that solvent make-up contributes only a small fraction of the total capture cost compared to energy consumption, carbon tax, and cargo-loss penalties. As a result, uncertainties in solvent degradation rates have a limited impact on our primary conclusions.

Regeneration heat is a more influential factor. In the revised manuscript, we have re-evaluated and updated the regeneration-heat values for NE-2 and DES to more realistically reflect the low CO_2 concentration in ship-exhaust gases (NE-2: 2.2 → 2.54 GJ t^{-1} CO_2; DES: 3.2 → 3.57 GJ t^{-1} CO_2; ***Supplementary Note 4***). These adjustments result in increased energy consumption and capture costs, but they do not change the relative ranking of the solvents or the preferred regeneration scheme.

(7) "*Port infrastructure and logistics –The assumption that CO_2-rich solvents can be offloaded at every port is highly idealized. In practice, solvent transfer logistics, storage infrastructure, and safety regulations may vary widely. The authors should provide a discussion of infrastructure requirements and realistic port compatibility, potentially including a scenario where offloading is only available at major hub ports.*"

We thank the reviewer for raising this important concern. We agree that, in practice, not all ports will be equipped with solvent regenerator. Although our main analysis assumed offloading at every port for modelling consistency, this does not imply that onshore regeneration would require offloading at every port in a real-world deployment.

To address the reviewer's concern, we have conducted an additional analysis to demonstrate that the onshore regeneration scheme remains competitive even when only a subset of ports is available for solvent offloading. In operation, rich solvent accumulates onboard as the ship sails. If the allowed load limit is not reached on a segment, no offloading is required and the rich solvent continues to accumulate. Once the rich-solvent load reaches the load limit, the capture system is shut down and offloading becomes necessary at the next port of call, which we identify as a "must-offload port".

In this new analysis, the load limit is treated as a decision variable rather than a fixed parameter. We reformulate the unit penalty as a function of the load limit by integrating the full set of cost and mass-balance equations under the accumulation-offloading logic described above. The optimal load limit is then obtained by solving the optimization problem to minimize the unit penalty. The ports where load limits are reached define the minimal set of must-offload ports required for a specific route.

This additional analysis demonstrates that the onshore regeneration scheme does not require solvent offloading at every port. It remains feasible and economically viable under realistic port-infrastructure constraints. A complete description of the calculation procedure and the resulting optimal offloading patterns is provided in ***Supplementary Note 19***.

Page 14 in the revised manuscript:

"***Flexible solvent offloading for DES onshore regeneration scheme***

Based on the preceding analysis, the DES-based onshore regeneration scheme emerges as the most cost-effective option under most conditions. In practice, however, solvent offloading infrastructure may not be available at every port. To account for this constraint, we introduce another flexible offloading strategy, in which a subset of "must-offload" ports is identified for each route by minimizing its unit penalty (Fig. 6a; Supplementary Note 19). As shown, most routes can reduce offloading frequency substantially, whereas TP17 Westbound and Eastbound remain exceptions. These routes feature high freight rates and consequently need more frequent rich-solvent offloading to save cargo-loss cost.

As shown in Fig. 6b, SBCC remains uneconomical in 2030, consistent with the baseline results where rich solvent can be offloaded at every port. In 2040, only one route achieves a unit penalty below the corresponding carbon tax, in contrast to the multiple routes identified under the baseline scenario (Fig. 5b). This performance decline stems mainly from the increased onboard accumulation of CO_2-rich solvent, which results in a higher cargo-loss penalty and reduces the effective capture rate. In 2050, all routes become economically viable, indicating that the DES-based onshore regeneration scheme remains promising even when offloading infrastructure is only available at a limited number of ports."

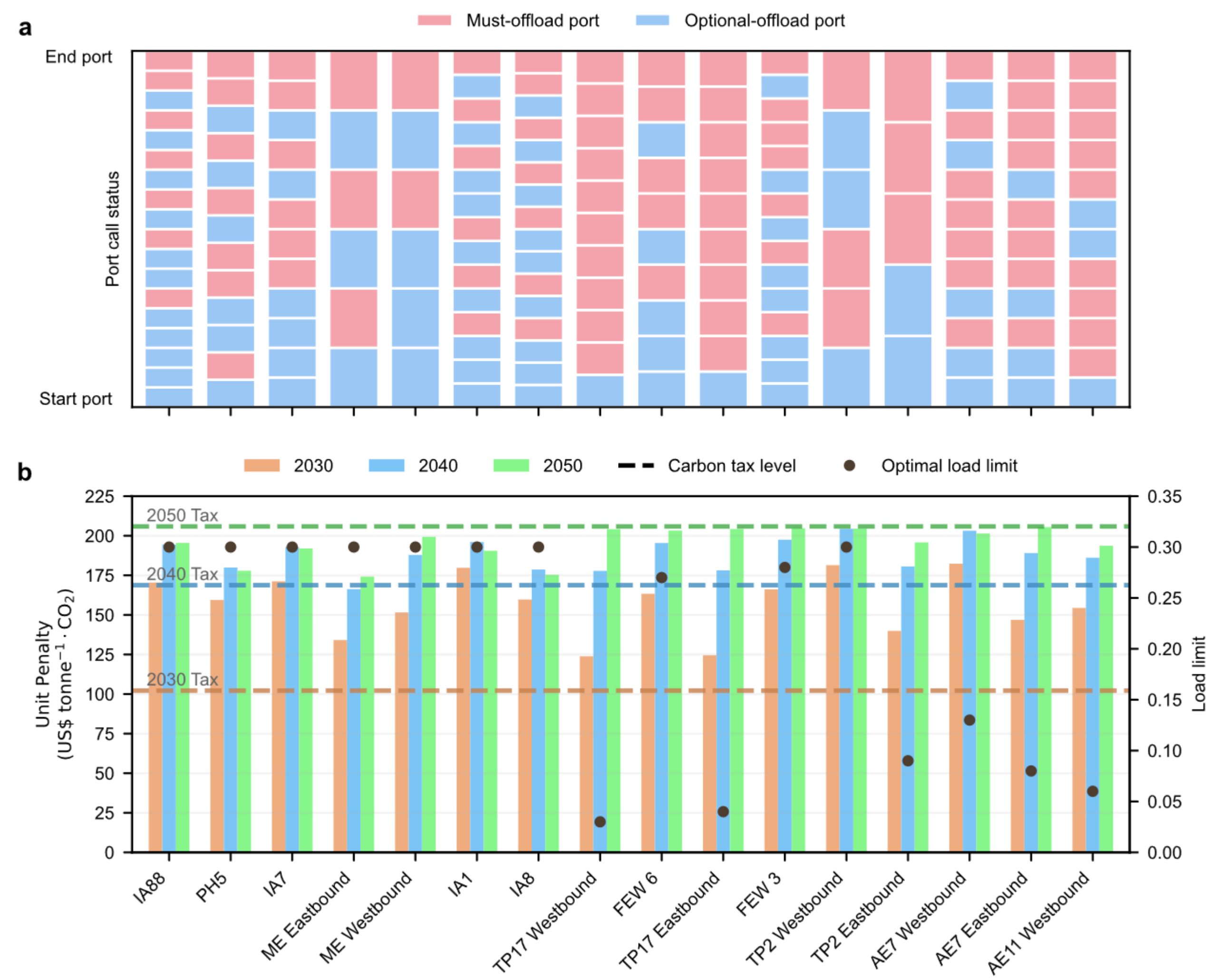

Fig. 6 | Flexible rich-solvent offloading results for the DES-based onshore regeneration scheme. ***a***, *Route-specific port offloading status under the flexible offloading strategy, where ports are classified as must-offload (red) or optional-offload (blue) based on minimization of the unit penalty.* ***b***, *Unit penalty under the flexible offloading strategy for different routes in 2030, 2040, and 2050. Bars indicate the unit penalty, dashed horizontal lines denote carbon tax, and black dots represent the corresponding optimal SBCC load limit.*

(8) "*Contextualization of novelty –The concept of onshore solvent regeneration is genuinely innovative and provides a meaningful contribution to the literature on ship-based carbon capture and port-based carbon management infrastructure. The comparative analysis across multiple routes, solvents, and carbon tax scenarios also enhances the practical relevance of the study. However, the manuscript does not sufficiently emphasize how its contribution advances beyond existing techno economic studies of SBCC. Prior research has already highlighted issues such as storage requirements and cost*

implications in ship-based carbon capture systems. I recommend that the authors more clearly situate their novelty in relation to this earlier work and articulate in what specific ways their analysis provides new insights—for example, through the expanded global route coverage, solvent specific techno-economic integration, or explicit linkage to long-term carbon tax scenarios. Such clarification would help to better establish the originality and added value of this study."

We sincerely thank the reviewer for acknowledging the novelty of our proposed onshore solvent regeneration concept and for the constructive suggestions. In response, we have revised the manuscript to more explicitly summarize the originality and added value of this work within the existing literature.

In the **Introduction**, we now provide a more structured overview of prior SBCC techno-economic studies and **explicitly outline their primary limitations**. Specifically, earlier work often (i) assumes near-complete utilization of exhaust-gas waste heat, (ii) discusses storage requirements and cargo loss only qualitatively without integrating them into cost calculations, (iii) neglects the additional CO_2 emissions induced by SBCC operation itself, and (iv) does not assess uncaptured emissions within regulatory carbon-tax frameworks. **We now clearly state that our analysis addresses these gaps** by simultaneously incorporating cargo-capacity loss, solvent initial inventory and continuous make-up costs, regeneration energy and CO_2 conditioning costs, as well as carbon-tax penalties into a unified economic model.

Beyond refining individual cost components, this study extends the scope of SBCC techno-economic assessment in three significant directions: (i) linkage to long-term carbon-tax trajectories, including both uniform global tax and region-specific ETS-equivalent pricing; and (ii) expansion from single-route case studies to a large-scale analysis covering 357 global container-shipping routes, enabling the identification of system-level trends that cannot be observed in single-vessel analyses. **These methodological extensions are now explicitly highlighted in the Introduction.**

Accordingly, the **Abstract** and **Conclusions have been revised** to more clearly emphasize how the proposed onshore regeneration scheme, combined with solvent- and route-specific analysis, provides new insights into the practical feasibility and long-term role of SBCC under evolving carbon-pricing regulations.

We believe these revisions clearly summarize the novelty of the work relative to previous studies and demonstrate its unique contribution in advancing SBCC techno-economic analysis.

Reference

1. Feenstra, M. *et al.* Ship-based carbon capture onboard of diesel or LNG-fuelled ships. *International Journal of Greenhouse Gas Control* **85**, 1-10 (2019).

2. Luo, X. & Wang, M. Study of solvent-based carbon capture for cargo ships through process modelling and simulation. *Applied Energy* **195**, 402-413 (2017).

3. Eric Schreiber, K. M. *Evaluation of Integrating Amine‐Based Onboard Carbon Capture and Storage on a Commercial Tanker* MARAD. https://www.maritime.dot.gov/innovation/meta/meta-aptamus-occs-report-2025 (2025).

4. Einbu, A. *et al.* Energy assessments of onboard CO_2 capture from ship engines by MEA-based post combustion capture system with flue gas heat integration. *International Journal of Greenhouse Gas Control* **113**, 103526 (2022).

5. Cho, M., Seo, Y., Park, E., Chang, D. & Han, S. Process design and energy assessment of an onboard carbon capture system with boilers or heat pumps for additional steam generation. *Ships and Offshore Structures* **19**, 1309-1322 (2024).

6. Tavakoli, S. *et al.* Exploring the technical feasibility of carbon capture onboard ships. *Journal of Cleaner Production* **452**, 142032 (2024).

7. The World's First Full Process Onboard Carbon Capture and Storage (OCCS) system Case Sharing Seminar. *eworldship Press Release* (2024).https://www.eworldship.com/html/2024/Manufacturer_0522/203344.html (accessed 14 June 2025).

8. IEAGHG. *The Status and Challenges of CO_2 Shipping Infrastructures.* IEAGHG. https://ieaghg.org/publications/the-status-and-challenges-of-co2-shipping-infrastructures/ (2020).

9. *Marine Carbon Capture Technology Review.* United States Department of Transportation, Maritime Administration. https://www.maritime.dot.gov/innovation/meta/feasibility-shipboard-carbon-capture-and-storage-ccs-technology (2022).

10. McCoy, S. T. & Rubin, E. S. An engineering-economic model of pipeline transport of CO_2 with application to carbon capture and storage. *International journal of greenhouse gas control* **2**, 219-229 (2008).

11. Wang, Z. *et al.* Study of ship-based carbon capture optimization considering multiple evaluation factors and main engine loads. *Journal of Cleaner Production* **478**, 143996 (2024).

12. Liu, F., Qi, Z., Fang, M. & Ding, H. Pilot test of water-lean solvent of 2-(ethylamino) ethanol, 1-methyl-2-pyrrolidinone, and water for post-combustion CO_2 capture. *Chemical Engineering Journal* **459** (2023).

13. Trivedi, T. J., Lee, J. H., Lee, H. J., Jeong, Y. K. & Choi, J. W. Deep eutectic solvents as attractive media for CO_2 capture. *Green Chemistry* **18**, 2834-2842 (2016).

14. Ju, J., Choi, D., Cho, S., Yoo, Y. & Kang, D. Absorption characteristics and rheological properties of quaternized polyamine-based deep eutectic solvents for high performance CO_2 capture. *Chemical Engineering Journal* **496**, 153922 (2024).

15. Foorginezhad, S. & Ji, X. Development of monoethanolamine chloride-ethylene diamine deep eutectic solvent for fffficient carbon dioxide capture. *Separation and Purification Technology* **347**, 127593 (2024).

16. European Union. Commission Delegated Regulation (EU) 2016/2071 of 22 September 2016 amending Regulation (EU) 2015/757 as regards the methods for monitoring carbon dioxide emissions and the rules for monitoring other relevant information.*Official Journal of the European Union* **L 320**, 1-6 (2016)

17. European Union. Directive (EU) 2023/959 amending Directive 2003/87/EC establishing a system for greenhouse gas emission allowance trading within the Union.*Official Journal of the European Union* **L 130**, 134 – 202 (2023)

18. Partnership, I. C. A. *Emissions Trading Worldwide: Status Report 2025.* International Carbon Action Partnership. https://icapcarbonaction.com/en/publications/emissions-trading-worldwide-icap-status-report-2025 (2025).

19. Commission, E. *The EU ETS and MRV Maritime: General guidance for shipping companies.* European Commission, Directorate-General for Climate Action. https://climate.ec.europa.eu/eu-action/eu-emissions-trading-system-eu. (2025).

Second Revision

Rethinking Maritime Carbon Capture: Onshore versus Onboard Solvent Regeneration
(Manuscript ID: NCOMMS-25-52243B)

To the Reviewers:

We sincerely thank the reviewers for their careful evaluation of our manuscript and for the additional constructive comments. In this revision, we have addressed all comments. Detailed point-by-point responses to each comment are provided below. All changes in the manuscript have been highlighted in red.

Reviewer #1

The revised paper shows significant improvement in quality. However, the author argues that the waste heat recovery for regeneration is negligible, which diverges from most shipboard carbon capture-related studies. This viewpoint requires further in-depth validation. Additionally, LNG accounts for a substantial proportion of ocean-going vessels, and its cold energy utilization potential should not be overlooked. These factors have important implications for the conclusions of this paper and necessitate relevant discussions.

We thank the reviewer for this thoughtful comment. We agree that our previous wording regarding waste-heat utilization for solvent regeneration required clearer qualification, and we also agree that the potential utilization of cold-energy in LNG-fueled vessels deserves discussion. In response, we have revised the manuscript and expanded the discussion as follows.

(1) Waste-heat recovery for solvent regeneration

The key issue is not whether waste heat can or cannot be used for solvent regeneration, but whether, under realistic ship operation, **any waste heat remains** after being used for other mandatory onboard heating demands. Modern ocean-going ships are extensively energy-integrated in response to IMO energy-efficiency and carbon-intensity

requirements.[1,2] In such ships, **waste heat recovery units (WHRU)** are already a mature and widely applied onboard technology.[3] **Figure 1** provides a representative Grassmann diagram of a ship energy system, illustrating that the recovered heat from engine exhaust gas is already embedded in a broad and highly integrated energy utilization network.[4] **As shown in the blue dashed box, this recovered heat is primarily used to satisfy various onboard heating demands**, including fuel heating, hoteling-related heat demand, and boiler-feedwater preheating. In other words, **it is not a heat source that can be freely used for solvent regeneration.**

PUBLISHED DATA FIGURE REDACTED

Figure 1. Grassmann diagram of the ship energy flow. Adapted from Ref. [4].

To further illustrate this operational constraint, **Figure 2** presents a more focused comparison between WHRU steam supply and onboard steam demand for an 82,000 dwt bulk carrier. Based on the steam balance, the onboard service-steam demand is estimated to be 849 kg h^{-1} under standard ISO conditions and 1,256 kg h^{-1} under cold conditions. However, the WHRU can meet this demand only under limited operating conditions; most of the time, additional steam must be supplied by auxiliary oil-fired boilers.[5] These energy balances indicate that the waste heat available for solvent regeneration is usually very limited, highly uncertain, and in many cases negligible.

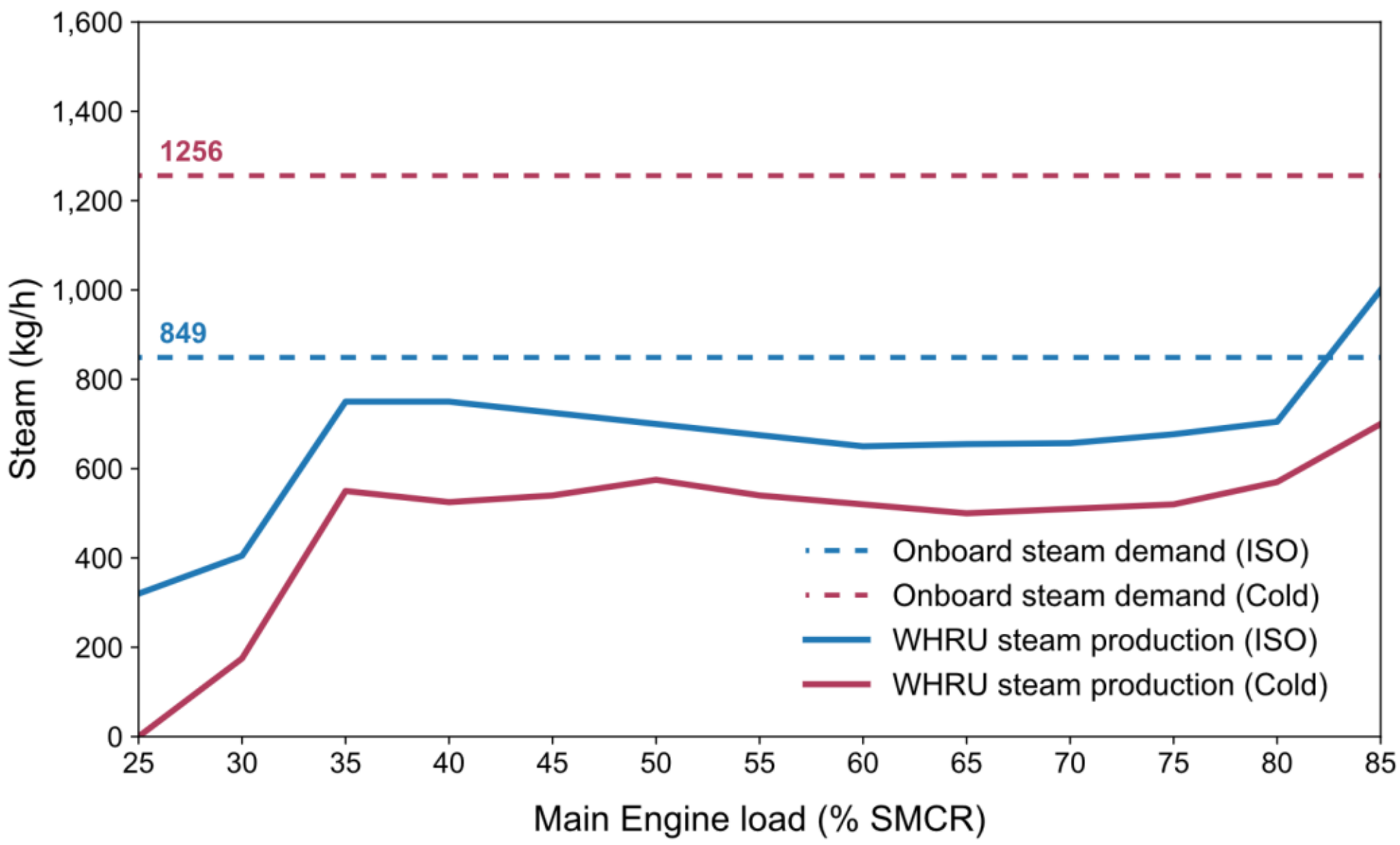

Figure 2. Comparison of WHRU steam production and onboard steam demand under ISO and cold operating conditions (data from Ref. [5])

Despite the above conclusion, one may still want to use the recovered heat to regenerate solvent. **However, reallocating recovered heat to solvent regeneration can lead to a shortage in service-steam supply**, which must then be compensated by additional fuel consumption, leading to an increase in carbon emissions. This operational constraint is not always explicitly discussed in existing SBCC studies.[6-9] As the reviewer pointed out, **many shipboard carbon capture studies ignore the onboard heating demands and assume that the recovered heat can be freely used for solvent regeneration. Unfortunately, this assumption is generally not valid in practice.**

Recent industrial and academic assessments further support the above viewpoint. The META program evaluated the integration of an amine-based onboard carbon capture system on a commercial tanker and reported that, when operating on HFO, the ship's existing WHRU capacity was already fully utilized by onboard heating demand, leaving limited residual heat for solvent regeneration.[10] Consistent conclusions were also reported by Tavakoli et al.[11] who showed that running the carbon capture units requires additional boiler operation, leading to a substantial increase in fuel consumption—about 70% – 100% relative to a baseline vessel without carbon capture.

Based on the above analysis, we have refined the manuscript to clearly state that the issue is not whether the onboard waste heat exists, but that the waste heat remaining available for solvent regeneration is, in many cases, negligible once mandatory onboard heating demands (e.g., fuel heating and hoteling-related heating) are satisfied.

Page 3 in the revised manuscript:

"*Despite being promising, OCCS is still in its early stages.*[6,45] *A key limitation in many studies is the assumption that exhaust-gas waste heat can be made available for solvent regeneration.*[24-27,46,47] *In practice, unfortunately, most recovered exhaust heat on ocean-going vessels is already committed to mandatory onboard heating demands, such as fuel heating and hoteling-related heating, leaving only limited residual heat for OCCS use.*[48,49] *Therefore, additional fuel must be consumed to support solvent regeneration. For example, a recent study on a commercial tanker reported that the additional fuel required to supply OCCS utilities would increase the ship's CO_2 emissions by 43%.*[50] *Tavakoli et al. further showed that to achieve 70–90% emission reductions can increase the total fuel consumption by 70－100% relative to a baseline vessel without carbon capture.*[21]"

(2) LNG cold-energy utilization

We appreciate the comment regarding LNG and the potential utilization of cryogenic cold energy. However, we want to emphasize that **our study is limited to ships using conventional oil-based fossil fuel, not LNG-fueled ships**. In fact, conventional-fuel ships accounted for 98.8% of the global fleet in 2022, while alternative-fuel ships (including LNG ships) represented only 1.2%.[12]

We have clarified this scope explicitly in the revised manuscript. Future work will extend the present framework to alternative fuel ships as their deployment increases. Alternative fuels include two types: carbon-containing fuels (e.g., LNG and methanol) where onboard CCS may remain relevant, and carbon-free fuels (e.g., hydrogen and ammonia) where CCS is not applicable.

Page 2 in the revised manuscript:

"*Green shipping using alternative fuels (e.g., LNG and green methanol) can help decarbonize the maritime industry.*[8-14] *However, compared to conventional fuels, their lower energy density and, in many cases, higher costs currently make them less competitive.*[5,15] *Conventional oil-based fossil fuels still power 98.8% of the active fleet and account for 78.9% of new ship orders.*[16]"

Page 4 in the revised manuscript:

"*In this study, we evaluate the technical feasibility and economic viability of onshore and onboard regeneration schemes for 16 selected Maersk container shipping routes, each represented by the most frequently operating vessel class using conventional marine fuels.*[55]"

Reviewer #4

The manuscript is interesting and worth publishing. The authors have already revised significantly their paper to take into account the good comments from the reviewers. I have a few comments to add.

We thank the reviewer for the positive evaluation. All the comments have been carefully addressed in the revised manuscript.

(1) The authors use the term SBCC, while the vast majority of scientific and industry discussion talk about OCCS (onboard carbon capture and storage). please stick to OCCS.

We thank the reviewer for this suggestion. In response, we have revised this terminology throughout the manuscript.

(2) The authors make it sounds like they came up with the onshore regeneration concept for OCCS. However, this is already what Value Maritime is commercialising: https://valuemaritime.com/products-services/. This should be made clearer.

We thank the reviewer for highlighting the work of Value Maritime. We fully agree that ongoing industrial developments should be acknowledged clearly in the manuscript.

However, we would like to point out that when this study was initiated in late 2023, we reviewed the existing OCCS literature and publicly available information on commercial developments. At that time, Value Maritime's system was presented primarily through the "CO_2 Battery" concept, without indicating that the storage medium was CO_2-rich amine solvent.[13,14]

We provide the above clarification simply to avoid any misunderstanding that the present study adopted an existing industrial idea and presented it as our own. We have therefore revised the manuscript to acknowledge industrial developments such as Value Maritime's system and to clarify that the contribution of this work lies in the quantitative techno-economic evaluation rather than in claiming conceptual novelty.

Page 4 in the revised manuscript:

"Similar concepts have also been explored in industrial practice, such as Value Maritime's Filtree system.[54]"

(3) *From my understanding, PZ is a banned solvent at-sea?*

Thank you for raising this important issue. We agree that the suitability of piperazine (PZ) for OCCS requires clarification. After reviewing the current OCCS guidance and classification-society documents, we did not identify any explicit international regulation that bans PZ for onboard carbon capture use.

We fully agree that some publications have raised concerns about the environmental suitability of PZ, particularly its poor biodegradability in the marine environment. For example, Ros et al.[15] excluded PZ and PZ-derived blends from their OCCS solvent screening on this basis. These concerns, however, relate primarily to the consequences of potential environmental release rather than to a categorical prohibition of onboard use. Under similar screening criteria, other amine solvents considered widely for OCCS use in the literature, such as MDEA and AMP, could raise the same concern.[16,17]

According to industrial practice, whether an amine is acceptable for OCCS use is typically based on a comprehensive risk assessment including solvent containment, system integration, and operational safety.[18] If the identified risks can be reduced to an acceptable level through formal safety assessments such as HAZID and HAZOP, the system can be considered acceptable.[10] Major classification societies, including DNV, Lloyd's Register, and ABS, generally apply this risk-based assessment framework.[19-21]

Importantly, as stated in our manuscript, PZ is included only as an example of the second-generation amine solvent, representing solvents with favorable absorption kinetics and moderate regeneration energy. In our results, PZ is not the preferred option. NE-2 performs better in the onboard-regeneration case, while DES is preferred in the onshore-regeneration case. Retaining PZ therefore serves only a benchmarking purpose and does not alter the main conclusions of this study.

(4) *The authors presents the result per route however it is difficult to understand the results from this. One would expect that presenting the results as a function of the distance would be the way to go as it is the critical parameter behind the route.*

We thank the reviewer for this suggestion and agree that voyage distance is an important route descriptor that influences the economic performance of OCCS systems. Accordingly, **we re-examined the route-level results as a function of sailing distance** (**Figure 3**). This alternative presentation shows **an overall tendency for total capture cost to increase with route distance**, which is expected because longer voyages are generally associated with

greater fuel consumption and higher CO_2 emissions. However, **the relationship is not monotonic, and distance alone does not explain the route-level results.** In other words, routes with similar sailing distances can still exhibit quite different costs. For example, routes PH5, TP2 W, and IA88 (highlighted in red box) have similar sailing distances of around 15,000 km but show substantially different capture costs. This difference arises because TP2 W is served by a much larger vessel (14,000 TEU) than PH5 (2,546 TEU) and IA88 (2,086 TEU), resulting in a substantially higher emission and capture cost. In addition, the longest voyage segment in TP2 W is 20 days, whereas the longest legs in PH5 and IA88 are only 6 and 5 days, respectively. Under the same load-limit constraints, TP2 W therefore incurs higher cargo-loss penalties, while its longer sailing duration causes a larger fraction of CO_2 to remain uncaptured, resulting in higher carbon-tax payments.

From the above analysis, it is clear that the techno-economic performance of each route is governed by multiple factors. In addition to distance, other influencing parameters include ship size, cargo capacity, carbon price, inter-port voyage duration, and load limits.

Because this distance-based analysis uses the same dataset as Figure 5, which is already presented in ascending order of TEU and discussed in detail, we chose not to add another separate figure. **Instead, to address the reviewer's concern, we provide an online interactive visualization tool** (https://occs-cost-explorer.onrender.com/) **in the revision, allowing readers to quickly examine the capture costs as functions of sailing distance and all the other influencing parameters.** We believe this provides a more intuitive way to present the multidimensional nature of the results.

Page 12 in the revised manuscript:

"*Given the multidimensional nature of the cost evaluation, an online interactive visualization tool is provided to allow readers to quickly examine the capture cost and TCT results under specified parameters, such as sailing distance and transportation time.*"

Data availability

"*An interactive visualization tool is publicly available at: https://occs-cost-explorer.onrender.com/.*"

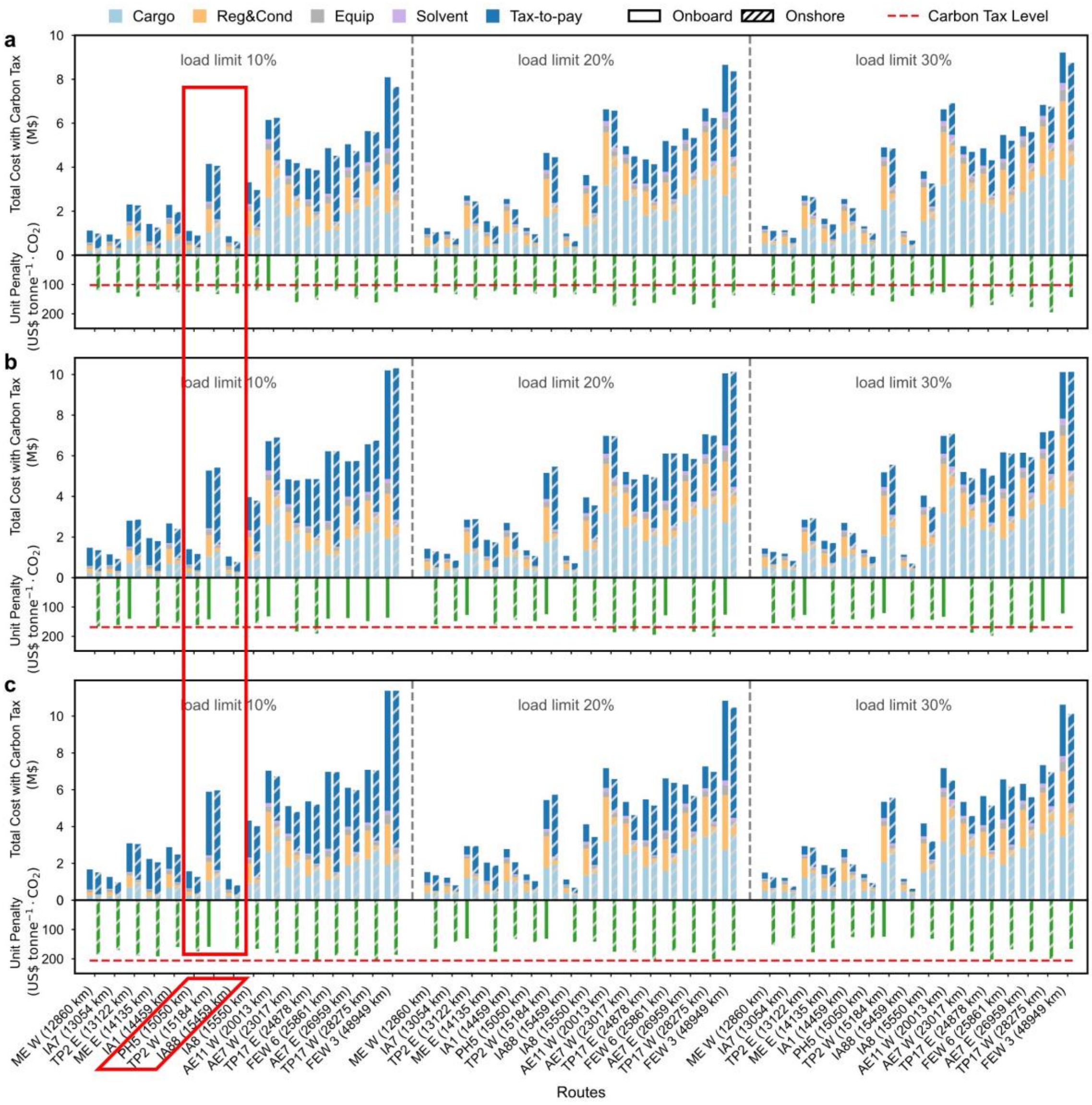

Figure 3. Total cost of OCCS and unit penalty across 16 routes under future carbon-tax scenarios. Subfigures (a–c) present results for the policy years 2030, 2040 and 2050, respectively. Within every subfigure, the upper chart shows the total cost with carbon tax (TCT) at three OCCS load limit constraints—10%, 20% and 30%. Stacked bars partition the TCT into cargo loss cost (light blue), regeneration plus conditioning cost (orange), fixed equipment cost (grey), solvent cost (purple) and carbon tax expenditure (dark blue). Solid bars correspond to the onboard regeneration scheme using NE-2, whereas diagonally hatched bars represent the onshore regeneration scheme using DES as the solvent. Routes are ordered by voyage distance from shortest to longest. The lower chart in each subfigure compares the prevailing carbon tax value (red dash line) with the unit penalty imposed by the more economical regeneration scheme.

References

1. IMO. *2014 Guidelines on the method of calculation of the attained energy efficiency design index (EEDI) for new ships. Resolution MEPC.245(66)*. (2014).

2. IMO. *Initial IMO strategy on reduction of GHG emissions from ships.Resolution MEPC.304(72)*. (2018).

3. IMO. *IMO Train the Trainer (TTT) Course on Energy Efficient Ship Operation: Module 4 – Ship Board Energy Management*. (2016).

4. Baldi, F., Johnson, H., Gabrielii, C. & Andersson, K. Energy and exergy analysis of ship energy systems – the case study of a chemical tanker. *Int. J. Thermodyn.* **18**, 82-93 (2015).

5. MAN Energy Solutions. *Economiser energy control for increased service steam production.* (2022).

6. Feenstra, M., Monteiro, J., Van Den Akker, J. T., Abu-Zahra, M. R. M., Gilling, E. & Goetheer, E. Ship-based carbon capture onboard of diesel or LNG-fuelled ships. *Int. J. Greenh. Gas Control* **85**, 1-10 (2019).

7. Long, N. V. D., Lee, D. Y., Kwag, C., Lee, Y. M., Lee, S. W., Hessel, V. et al. Improvement of marine carbon capture onboard diesel fueled ships. *Chem. Eng. Process.: Process Intensif.* **168**, 108535 (2021).

8. Oh, J., Kim, D., Roussanaly, S., Anantharaman, R. & Lim, Y. Optimal capacity design of amine-based onboard CO_2 capture systems under variable marine engine loads. *Chem. Eng. J.* **483**, 149136 (2024).

9. Einbu, A., Pettersen, T., Morud, J., Tobiesen, A., Jayarathna, C., Skagestad, R. et al. Energy assessments of onboard CO_2 capture from ship engines by MEA-based post combustion capture system with flue gas heat integration. *Int. J. Greenh. Gas Control* **113**, 103526 (2022).

10. MARAD and OSG Ship Management, Inc. *Evaluation of Integrating Amine‑Based Onboard Carbon Capture and Storage on a Commercial Tanker* (2025).

11. Tavakoli, S., Gamlem, G. M., Kim, D., Roussanaly, S., Anantharaman, R., Yum, K. K. et al. Exploring the technical feasibility of carbon capture onboard ships. *J. Clean. Prod.* **452**, 142032 (2024).

12. UNCTAD. *Review of Maritime Transport 2023*. (2023).

13. Wu, J., Zhang, Y., Jiang, M., Cao, X. E. & Zhang, Z. Onboard carbon capture, utilization, and storage. *Cell Rep. Phys. Sci.* **7**, 103034 (2026).

14. Value Maritime. *VALUE MARITIME announces installation of the world's first onboard CO_2 capture and storage unit on an operational vessel*. https://web.archive.org/web/20251019050300/https://valuemaritime.com/news/value-maritime-announces-co2-capture-unit-2/ (accessed Oct 19 2025).

e, V., Van Den Akker, J. T., Vredeveldt, A. W., Linders, M. J. G. et al. Advancements in ship-based carbon capture technology on board of LNG-fuelled ships. *Int. J. Greenh. Gas Control* **114**, 103575 (2022).

16. Eide-Haugmo, I., Brakstad, O. G., Hoff, K. A., da Silva, E. F. & Svendsen, H. F. Marine biodegradability and ecotoxicity of solvents for CO_2-capture of natural gas. *Int. J. Greenh. Gas Control* **9**, 184-192 (2012).

17. The Bellona Foundation. *Amines used in CO_2 capture-health and environmental impacts*. (2009).

18. Maritime Technologies Forum. *Safe onboard carbon capture and storage*. (2025).

19. DNV. *Rules for classification: Ships. Part 6 Additional class notations, Chapter 7 Environmental protection and pollution control*. (2025).

20. Lloyd’s Register. *Rules and Regulations for the Classification of Ships, Notice No. 1, 2 & 3 , Part 5 Main and AuxiliaryMachinery , Chapter 24 Emissions Abatement Plant for CombustionMachinery and other Machinery and Equipment, Section 13 Carbonemissions abatement plant*. (2024).

21. ABS. *Requirements for Onboard Carbon Capture and Storage*. (2023).
